# Enhancing the Equilibrium of Dynamic Thia-Michael Reactions through Heterocyclic Design

**DOI:** 10.1021/jacs.3c03643

**Published:** 2023-06-23

**Authors:** Alex E. Crolais, Neil D. Dolinski, Nicholas R. Boynton, Julia M. Radhakrishnan, Scott A. Snyder, Stuart J. Rowan

**Affiliations:** † Department of Chemistry, University of Chicago, Chicago, Illinois 60637, United States; ‡ Pritzker School of Molecular Engineering, University of Chicago, Chicago, Illinois 60637, United States

## Abstract

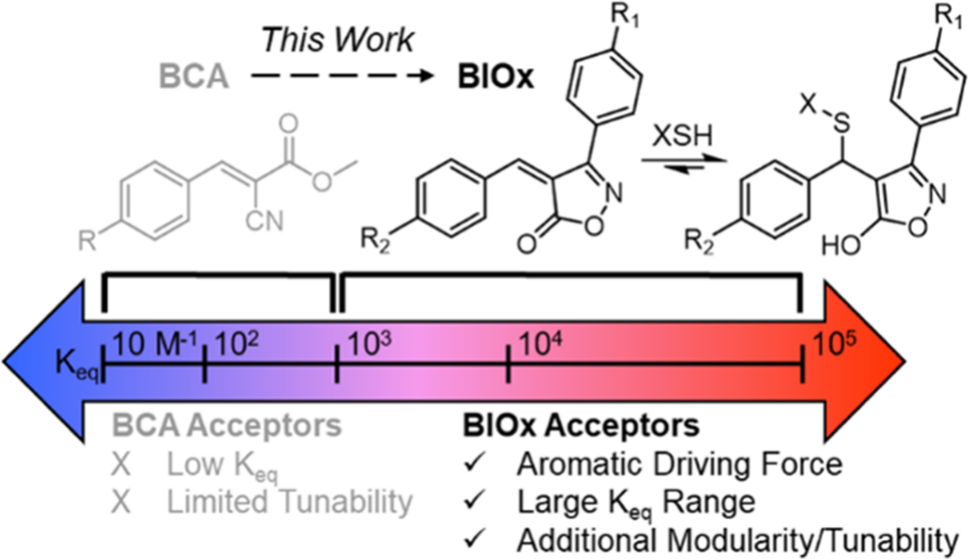

Although the catalyst-free dynamic thia-Michael (tM) reaction has been leveraged for a range of significant applications in materials science and pharmaceutical development, exploiting its full potential has been limited by relatively low equilibrium constants. To address this shortcoming, a new series of catalyst-free, room-temperature dynamic thia-Michael acceptors bearing an isoxazolone motif were developed and utilized to access both dynamic covalent networks and linear polymers. By leveraging the generation of aromaticity upon thiol addition and tuning the electronic-withdrawing/donating nature of the acceptor at two different sites, a wide range of equilibrium constants (*K*_eq_ ∼1000 to ∼100,000 M^–1^) were obtained, constituting a 2 orders of magnitude increase compared to their noncyclic benzalcyanoacetate analogues. Integration into a ditopic isoxazolone-based Michael acceptor allowed access to both bulk dynamic networks and linear polymers; these materials not only exhibited tailorable thermomechanical properties based on thia-Michael acceptor composition, but the higher *K*_eq_ tM bonds resulted in more mechanically robust materials relative to past designs. Furthermore, solution-state formation of linear polymers was achieved thanks to the increased *K*_eq_ of the isoxazolone-based acceptors.

## Introduction

The reversible and stimulus-responsive nature of dynamic covalent chemistries (DCC)^1,2^ have long been exploited to access polymeric materials for applications ranging from drug delivery systems^3,4^ and adhesives^5,6^ to recyclable^7^ and smart/adaptive materials.^8,9^ Perhaps the most fundamental design parameter of such structurally dynamic polymeric materials is the choice of dynamic covalent bond incorporated into the system. Indeed, this feature determines the underlying properties of the material by modulating the exchange rate(s), equilibrium constant, catalyst requirements, and/or the identity of the external stimuli that may induce a response.^10,11^ The variety of currently available dynamic bonds covers a wide swath of bonding partners, thereby facilitating a variety of synthetic installation approaches, with noteworthy examples including reversible Diels–Alder reactions,^12−16^ alkyl urea exchange,^17,18^ transesterification,^19,20^ disulfide and diselenide exchange,^21−23^ and many more.^24−28^ However, as the scope of potential applications for DCC broadens, there remains a pressing need for the development of new, reversible covalent bonds. In particular, catalyst-free dynamic chemistries are attractive as they avoid the potential issues of catalyst-leaching and corresponding changes over time. Additionally, DCCs that operate under ambient conditions also have likely advantages in enabling enhanced exchange under typical operating conditions, although generally at the cost of the thermomechanical properties of the material (without reinforcement^29^), an issue which has presumably limited their study in materials applications to date.^30^

**Figure 1:**
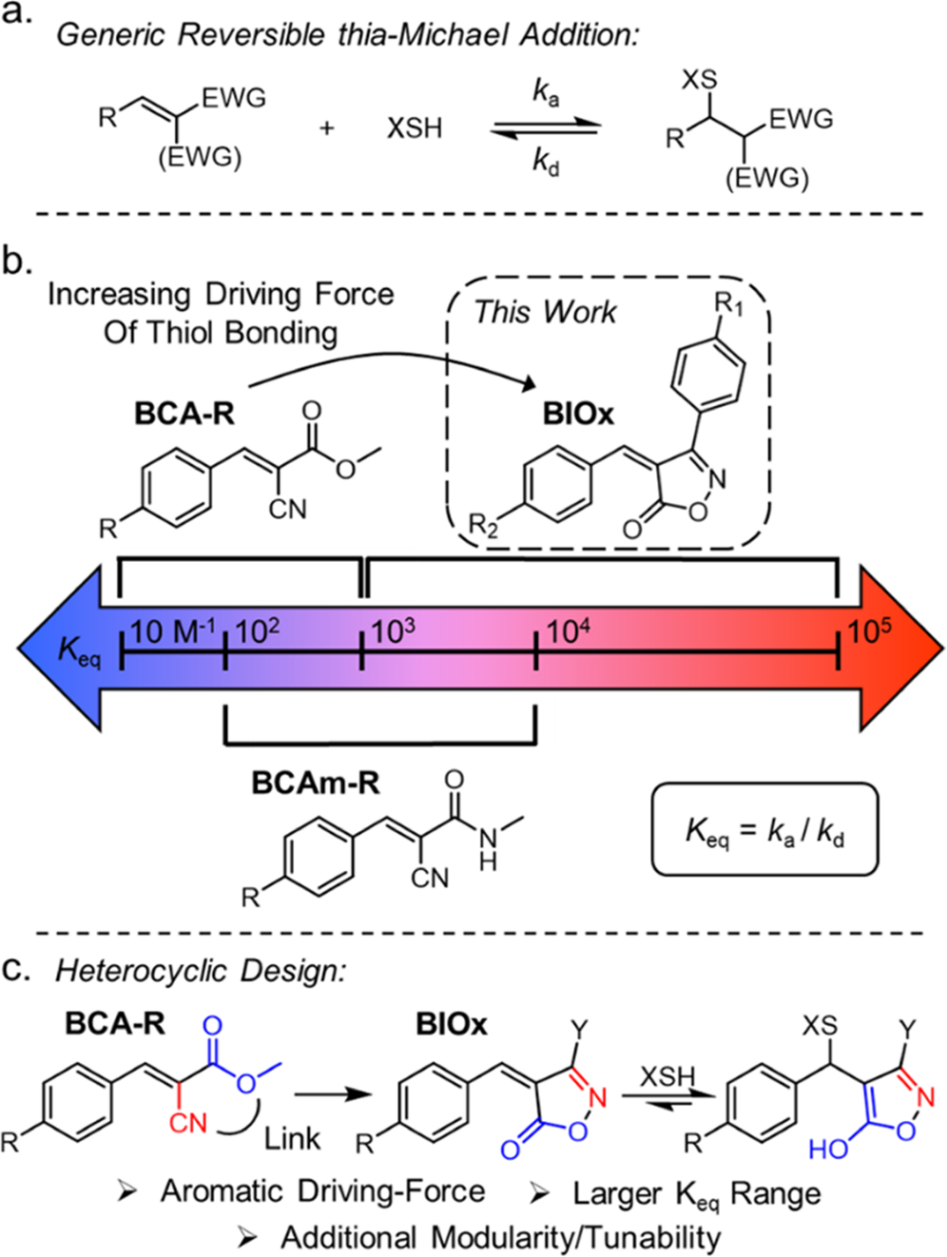
(a) General reaction scheme of dynamic thia-Michael reactions. Electron-withdrawing group in parentheses is not necessary for addition or reversibility. (b) Equilibrium constant (*K*_eq_) ranges of the addition of thiols to benzalcyanoacetate (BCA), benzalcyanoacetamide (BCAm), and 3-substituted-4-benzalideneisoxazol-5(4H)one (BIOx) Michael acceptors. (c) Design of the proposed BIOx acceptors inspired by the BCA moiety in which the addition of a thiol yields aromatic 5-hydroxy isoxazole derivatives, which offer larger *K*_eq_ range and increased tunability.

Thia-Michael (tM) chemistry, the conjugate addition of a thiol to an activated alkene under basic conditions, has been employed extensively in the synthesis and (post)functionalization of polymers.^31^ More recently, tM reactions have been explored as versatile, tunable dynamic bonding motifs. Notable examples include the use of thiol-acrylate^32^ and thiol-maleimide^33^ crosslinkers in self-healing materials. However, such dynamic tM materials require the presence of a base or elevated temperatures (90 °C) for exchange to occur. One interesting aspect of the tM reaction is its dynamic behavior can be tailored by the substituents on the activated alkene (Figure 1a), which has led to the continued exploration of tM bonds in dynamic networks.^34−36^ In particular, the benzalcyanoacetate (BCA) Michael acceptor, which has two electron-withdrawing groups, has allowed access to an interesting new class of dynamic systems.^37^ Pioneering work into the BCA acceptor motif by the Taunton and Anslyn groups has demonstrated that in a polar solvent, these acceptors undergo thiol exchange at room temperature and in the absence of any catalyst.^38,39^ Critically, varying the electron-donating/withdrawing properties of the β-phenyl ring has been shown to control the equilibrium constant, *K*_eq_, of the thiol addition significantly (from ∼10 to ∼1000 M^–1^).^39,40^ These BCA acceptors have since been successfully incorporated into mechanically robust dynamic covalent network (DCN) films with shape memory behavior when prepared with tetra-functional thiols^40^ or into multipurpose adhesives^41^ upon mixing with poly(mercaptopropyl methyl)siloxane (PMMS). Importantly, the properties of such DCNs were shown to be readily tuned through the choice of substitution on the β-phenyl ring of the acceptor. However, despite their promising performance, their low *K*_eq_ range has limited the use of BCAs to solid-state networks, where the materials are reinforced by an emergent dynamic reaction-induced phase separation (DRIPS).^40^

While much of the focus in tunable tM dynamic bonds has revolved around manipulating the electronics of the β-phenyl ring, there are other handles to manipulate the *K*_eq_. Any electron-withdrawing group (in the carbon acid precursor) directly attached to the double bond also has a significant effect on the thiol addition. For instance, it has been shown that replacing the ester in the BCA with an amide moiety results in a benzalcyanoacetamide (BCAm) Michael acceptor with enhanced thiol bonding, resulting in an ∼10× enhancement in *K*_eq_ (∼100 to ∼10,000 M^–1^) relative to BCAs.^39^ The stronger bonding of BCAm acceptors has given access to a wider range of dynamic materials such as hydrogels,^42,43^ stress-adaptive dense suspensions,^44^ and other dynamic covalent networks.^45^ Nevertheless, while these advances have already shown great promise in further manipulating the range of dynamic response in tM materials, the *K*_eq_ values are significantly lower than commonly employed supramolecular motifs used to access supramolecular polymers in solution (and in bulk).^46^ Further increasing the *K*_eq_ range of dynamic tM acceptors (Figure 1b) would continue to expand their range of applications, particularly in allowing access to linear polymers with significant degrees of polymerization and potential uses in biological systems where efficacy at a low concentration is a requisite.^38^

Reported herein are studies aimed at developing catalyst-free, room-temperature dynamic thia-Michael bonds with significantly enhanced equilibrium constants (up to ∼100,000 M^–1^) along with initial explorations for their use in dynamic covalent polymers. Inspiration from the general structure of the BCA acceptor led to the exploration of new heterocyclic dynamic tM acceptors, where an isoxazolone (Figure 1c) is the cyclic analogue of methyl cyanoacetate (essentially linking the cyano and ester groups) and, as such, an imine now serves as the second withdrawing group instead of the cyano moiety. While such 3-substituted-4-benzylideneisoxazol-5(4H)-ones (BIOx) have been previously studied as dyes and for applications in α-propargylations, their efficacy as Michael acceptors, to the best of our knowledge, has never been investigated.^47,48^ We hypothesized that the isoxazolone ring could offer two major advantages over its noncyclic analogue: (1) upon thiol addition to the BIOx acceptor, the resulting enol tautomer would yield an aromatic 5-hydroxy isoxazole moiety that could serve as a significant driving force for thiol addition and (2) a greater range of properties and equilibrium constants might be achieved given that these BIOx acceptors have an additional tuning handle through the “Y” position off of the imine linkage.

## Results and Discussion

**Figure 2:**
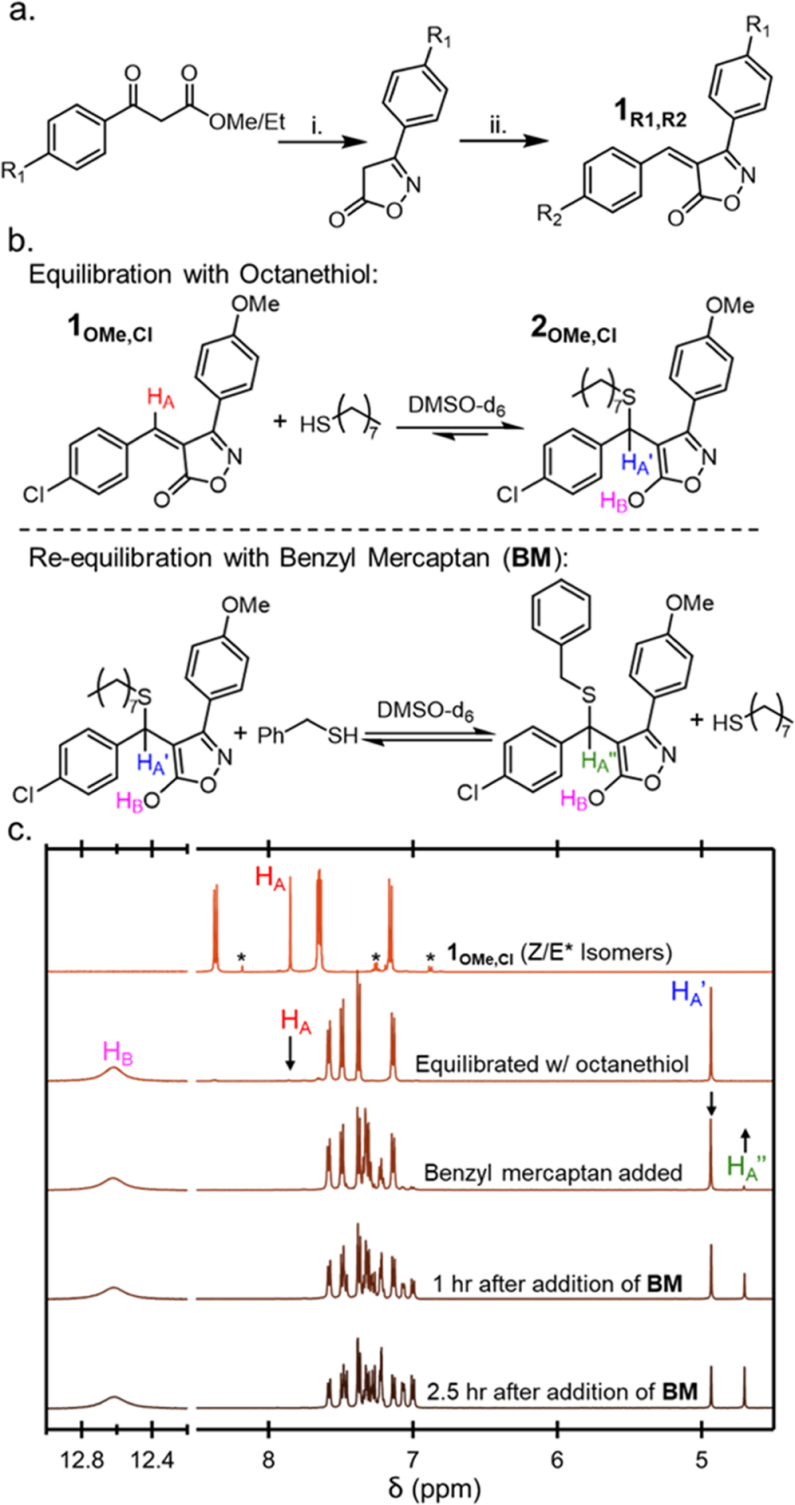
(a) Synthesis of **1**_**R1**,**R2**_ acceptors: (i) ketoester, hydroxylamine hydrochloride, NaOAc, ethanol, 50 °C, 93–99%; (ii) isoxazolone, benzaldehyde, piperidine, isopropanol, 50 °C, 25–90%. (b) Addition of nucleophile and (c) ^1^H NMR spectrum of **1**_**OMe**,**Cl**_, after equilibration with octanethiol and during second equilibration with benzyl mercaptan (BM). The broad peak at 12.6 ppm is magnified by 5× for clearer visualization.

As electronic substituents have been shown to have large effects on BCA tM acceptors, it was important to synthesize BIOx Michael acceptors with both the benzal moiety and a similarly substituted phenyl ring at the 3-position of the heterocycle (defined earlier as the “Y” position). The synthesis of the small-molecule BIOx acceptors could be accomplished in just two steps: (1) a ring closure between an appropriate aryl ketoester and hydroxylamine to yield the 3-substituted isoxazol-5(4H)-one and (2) a subsequent reaction with a substituted benzaldehyde via Knoevenagel condensation (Figure 2a). These materials were formed predominantly as the Z-isomer and could be synthesized in high yield (up to 90%).^48^ In total, a series of 12 of these small-molecule BIOx acceptors (**1**_**R1**,**R2**_) were prepared, where R_1_ and R_2_ are the *para*-substituents on the aromatic groups at the 3- and 4-positions of the isoxazol-5(4H)-one, respectively.

Initial small-molecule studies were performed to explore the reaction of a thiol with the BIOx acceptor **1**_**OMe**,**Cl**_. First, a solution of **1**_**OMe**,**Cl**_ (25 mM) was allowed to equilibrate with one equivalent of 1-octanethiol in DMSO-*d*_6_. Within minutes of the thiol addition, a clear colorimetric shift from yellow to colorless was observed. Under ^1^H NMR monitoring, nearly complete conversion of the olefin peak, H_A_, to **2**_**OMe**,**Cl**_, H_A_′ (Figure 2b) was readily identified. The emergence of a broad downfield peak, H_B_, at ∼12.5 ppm (along with the observation that H_A_′ is a lone singlet) confirmed the hypothesis that the aromatic enol product was formed upon thiol addition. These initial results demonstrated that BIOx acceptors allow the catalyst-free addition of a thiol in DMSO; however, it did not demonstrate that these tM adducts are dynamic at room temperature. To explore this element, one equivalent of benzyl mercaptan was then added to the equilibrated 1-octanethiol adduct solution and the resulting reaction was monitored *via* NMR. As shown in Figure 2b, shortly after the addition of the second thiol, a new peak, H_A_″, began to appear as peak H_A_′ diminished, indicating thiol exchange was occurring in solution at room temperature. After 2.5 h, the two peaks reached equal intensity and reached equilibrium, confirming that these BIOx tM adducts are indeed dynamic at room temperature.

**Figure 3:**
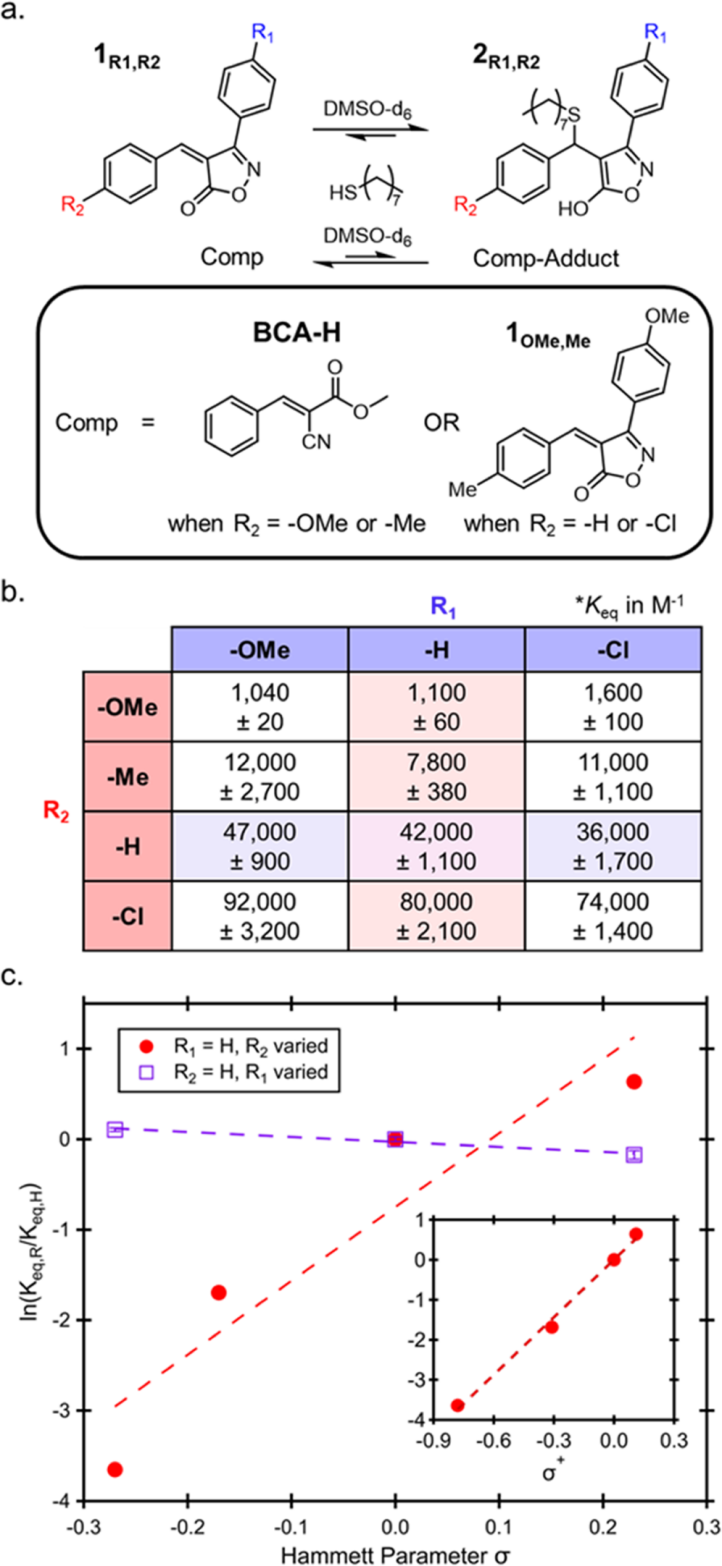
(a) Relevant equilibrium of competition *K*_eq_ study, (b) table of *K*_eq_ values of **1**_**R1**,**R2**_ tM acceptors, and (c) Hammett plot of **1**_**R1**,**H**_ (open square) and **1**_**H**,**R2**_ (red circle) sweeps (note that error bars are covered by the data points). The inset in (c) contains the **1**_**H**,**R2**_ sweep plotted using σ^+^, linearizing the data.

A signature feature of BCA acceptors is the ability to tune their *K*_eq_ through electronic modifications, as such an understanding of the impact of electronic modifications at the R_1_ and R_2_ positions is of interest for BIOx acceptors. To this end, the suite of **1**_**R1**,**R2**_ acceptors, whose R_1_ and R_2_ substituents range from electron-donating (-OMe; σ_para_ = -0.27) to electron-withdrawing (-Cl; σ_para_ = 0.23), had their equilibrium constants determined.^49^ In order to accurately determine the equilibrium constants of **1**_**R1**,**R2**_, competition bonding experiments were performed on all acceptors (Figure 3a,b, see the SI for full details of the experimental setup and data interpretation, Figures S1 and S2). Excitingly, the isoxazolone-based tM acceptors displayed a range of *K*_eq_ values significantly higher (∼100×) than any previous BCA acceptor, with the highest *K*_eq_ (ca. 9.2 ± 0.3 × 10^4^ M^–1^) being observed for the thiol addition to **1**_**OMe**,**Cl**_. Furthermore, consistent with previously measured dynamic Michael acceptors,^39^ the Hammett plot of the *K*_eq_ of the BIOx acceptors (Figure 3c) shows a positive trend (with a notable bend) with regard to the R_2_ range from 1,100 M^–1^ (**1**_**H**,**OMe**_) to 80,000 M^–1^ (**1**_**H**,**Cl**_); this result highlights the impact of the electronic nature of the *β*-phenyl ring on the equilibrium. It is worthy of note that plotting *K*_eq_ against σ^+^ Hammett parameters (Figure 3c inset) linearizes the plot, indicating a buildup of positive charge on the β-position in the transition state of the thia-Michael reaction. The importance of charge in the transition state can also be seen through changing solvent (polarity), with a significant slowdown in exchange measured when equilibration is carried out in CDCl_3_ (Figure S4). Interestingly, the electronic nature of the R_1_ substituent also had an effect on the thiol addition, albeit not as significant on account of R_1_ being further removed from the β-position, leading to an ∼25% reduction in *K*_eq_ from **1**_**OMe**,**H**_ to **1**_**Cl**,**H**_. While there does appear to be a degree of interactivity between R_1_ and R_2_ positions, resulting in a shift from positive to negative correlations of R_1_ with increasing R_2_ withdrawing character, changes in *K*_eq_ through manipulating R_1_ never exceeded ±2×. This finding implies that a combination of an electron-donating R_1_ position and an electron-withdrawing R_2_ position would lead to the highest *K*_eq_ values observed with **1**_**OMe**,**Cl**_.

With an understanding of how varied substituents impact the *K*_eq_ of the BIOx acceptors, a series of ditopic BIOx-bearing monomers were then prepared. As the R_2_ position was shown to have the largest impact on the *K*_eq_ value, ditopic monomers linked to the BIOx moieties through the R_1_ position were prepared. As the highest *K*_eq_’s are observed with R_1_ being an electron-donating group, ether bonds were chosen to attach the BIOx units. The synthesis of the requisite monomers was accomplished by generating two aryl ketoesters linked by triethylene glycol in the R_1_ position; these compounds were then converted to the ditopic acceptor through a procedure similar to Figure 2a (see the SI for full scheme and synthetic details).^50−52^ The synthesized ditopic BIOx monomers are referred to as **3**_**R**_, with R referring to the substituent at the R_2_ position.

**Figure 4:**
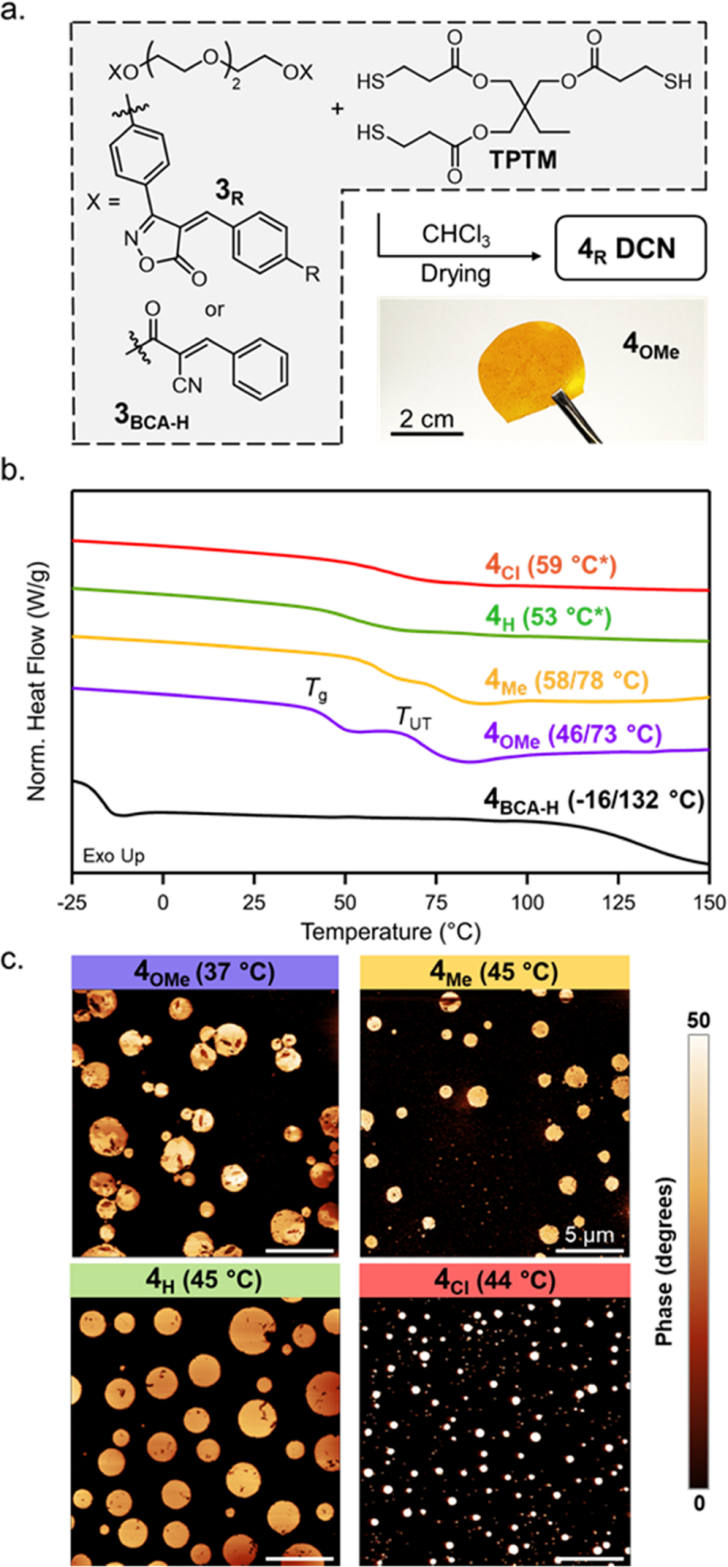
(a) Chemical structures used in the synthesis of bulk dynamic network and a representative photo of a **4_OMe_** network after pressing. (b) Differential scanning calorimetry (DSC) curves for **4**_**Cl**_ (red), **4**_**H**_ (green), **4**_**Me**_ (orange), **4**_**OMe**_ (blue), and **4**_**BCA-H**_ (black). Values in parentheses represent the midpoints of *T*_g_ and *T*_UT_, or the combined transition. (c) Atomic force microscopy (AFM) phase images of BIOx tri-thiol networks. Values in parentheses represent the temperature that the AFM was taken to maximize contrast.

With a series of ditopic monomers **3**_**R**_ in hand, a set of dynamic covalent networks (DCN) were prepared with a tri-thiol crosslinker trimethylolpropane tris(3-mercaptopropionate) (TPTM). To prepare the networks, the desired **3**_**R**_ species was dissolved in CHCl_3_ along with an equimolar (thiol/BIOx) amount of TPTM. The networks were then solution-cast and thoroughly dried to afford DCN films, **4**_**R**_ (Figure 4a). Complete removal of the CHCl_3_ was confirmed through thermogravimetric analysis (TGA) (Figure S5). As a first assessment of the thermal properties of these **4**_**R**_ networks, differential scanning calorimetry (DSC) was performed (Figure 4b). In agreement with previously reported BCA-based networks,^40^ both the **4**_**OMe**_ and **4**_**Me**_ curves display two clear thermal transitions, consistent with the presence of multiple phases, and suggest that these two DCNs undergo a similar dynamic reaction-induced phase separation (DRIPS) process to the BCA networks.^40^ The lower transition is attributed to the glass transition (*T*_g_) of the continuous “soft” phase, while the higher transition is associated with the “hard” phase domains (*T*_UT_). Interestingly, the higher *K*_eq_ materials (**4**_**H**_ and **4**_**Cl**_) displayed a singular broad transition, indicating that either there is no DRIPS process or that the *T*_g_ and *T*_UT_ have begun to converge. The convolution of *T*_g_ and *T*_UT_ was confirmed through modulated DSC of **4**_**H**_, which showed two transitions in the nonreversing heat flow curve (Figure S6). To further verify the phase-separated nature of these materials, atomic force microscopy (AFM) was carried out near *T*_g_ for each **4**_**R**_ network (Figure 4c), and in all cases, a phase-separated morphology was found. Similar to previous examples of DRIPS,^40^ the underlying equilibrium constants do not obviously correlate with the observed morphologies, indicating that additional factors (such as sterics, solubility, etc.) strongly contribute to the DRIPS process. While DRIPS is key to the robust properties of BCA-based networks, it was hypothesized that the enhanced equilibrium of BIOx-based networks would give access to thermomechanically robust materials properties above their relatively low *T*_UT_.

**Figure 5:**
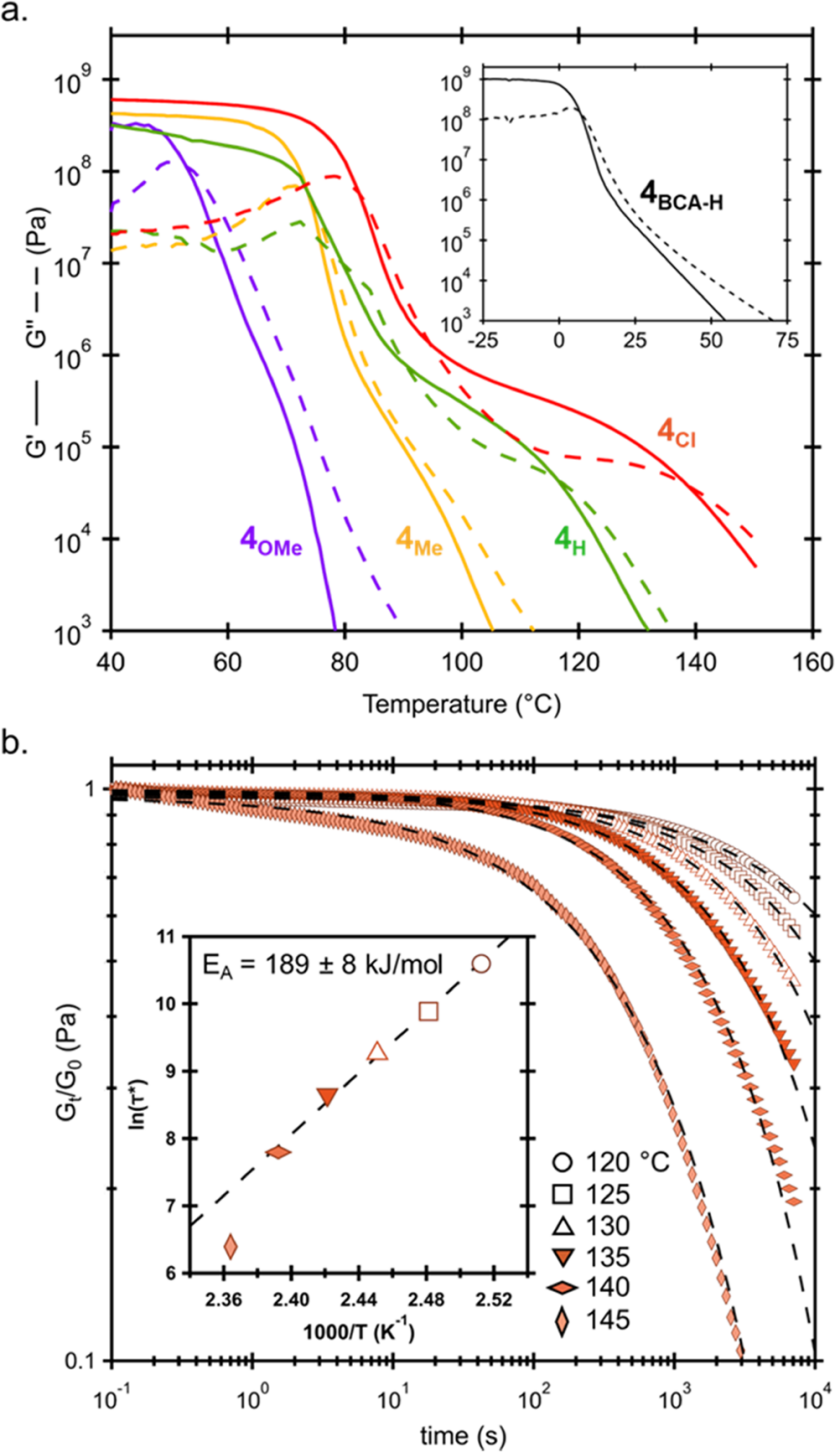
(a) Shear rheology (temperature ramp rate 3 °C/min, frequency = 1 Hz, parallel-plate geometry) for **4**_**Cl**_ (red), **4**_**H**_ (green), **4**_**Me**_ (orange), **4**_**OMe**_ (blue), and **4**_**BCA-H**_ (black) and (b) stress relaxation (3% strain) curves for **4**_**Cl**_ at 120 °C (open circle), 125 °C (open square), 130 °C (open triangle), 135 °C (orange triangle), 140 °C (horizontal diamond), and 145 °C (vertical diamond). Open plots indicate τ* extrapolated from the curve.

To further probe the effects of the equilibrium constant on the thermomechanical properties of the networks, small-amplitude oscillatory shear (SAOS) rheology studies were carried out (Figure 5a). In agreement with the trends in thiol affinity, the increased electron-withdrawing character of the key substituents was found to result in higher glass-transition temperatures (as defined by the peak in tanδ), ranging from ca. 65 to 85 °C from **4**_**OMe**_ to **4**_**Cl**_. The high *T*_g_ values of the **4**_**R**_ networks are in stark contrast to an analogous BCA-based network, whose *T*_g_ was found to only be ∼10 °C. Interestingly, the electron-rich crosslinkers (**4**_**OMe**_ and **4**_**Me**_), whose *K*_eq_ values are similar to the highest available BCA/BCAm acceptors, were found to freely flow shortly after rising above *T*_g_, due to insufficient bonding to maintain percolation at elevated temperatures (Figure 5a). However, further increases in the *K*_eq_ were found to lead to more robust behavior at higher temperatures, with both **4**_**H**_ and **4**_**Cl**_ displaying rubbery plateaus, with **4**_**Cl**_ having the highest modulus (extent of crosslinking) and largest range spanning ca. 100–140 °C. Importantly, all networks were found to readily undergo reprocessing through melt pressing conditions, with limited to no changes in thermal transitions, optical clarity, or thermomechanical properties across reprocessing steps (Figures S8–S10). It is worthy of note that while these materials can be reprocessed, it was found that extended exposure to temperatures ∼150 °C induces irreversible crosslinking events (Figure S11).

Given the extended plateau behavior of **4**_**Cl**_, additional stress relaxation experiments were carried out (Figure 5b). At all measured temperatures, the material was shown to relax and the resulting data is well described by a stretched exponential fit (eq 1).
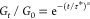
1This function is employed to describe a distribution of relaxation rates throughout the network and allowed for the determination of τ* for networks that did not relax beyond *G_t_*/*G*_0_ = e^–1^ (denoted by open symbols). The relaxation was found to be Arrhenius up to 140 °C, before deviating at higher temperatures, a feature observed before in dissociative networks.^53^ Further analysis of the stress relaxation data indicates that the apparent viscosity of the network drops precipitously above 140 °C (Figure S14), consistent with a significant decrease in network connectivity. Impressively, the extracted activation energy from the Arrhenius portion was found to be 189 kJ/mol, indicating the relaxation process (related to *k*_d_) is highly dependent on temperature.^54^ To further examine this effect, creep experiments were performed on **4**_**Cl**_ at various temperatures (Figure S15). In agreement with the results from the stress relaxation experiment, the rate of creep was highly dependent on temperature, with an ∼250× increase in the steady-state rate of creep over a 20 °C range (0.01 to 2.55 s^–1^ for 90 and 110 °C, respectively).

**Figure 6:**
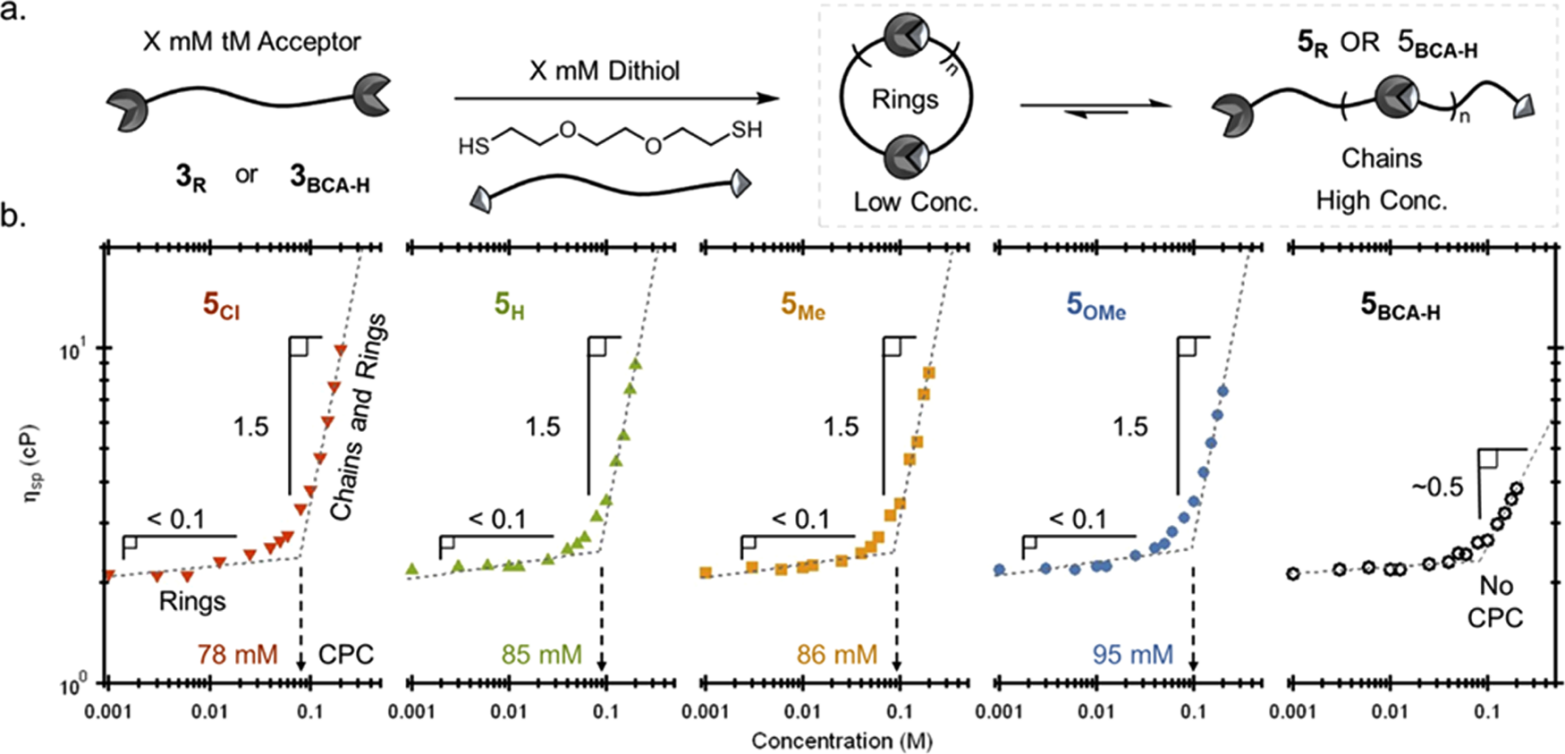
(a) Synthesis of **5**_**R**_ and **5**_**BCA-H**_ linear polymers and (b) log–log plots of zero shear viscosity versus concentration of **5**_**Cl**_ (red triangle), **5**_**H**_ (green triangle), **5**_**Me**_ (orange square), **5**_**OMe**_ (blue circle), and **5**_**BCA-H**_ (open circle).

While the ability to widely tune the bulk network properties through electronic modifications is an attractive feature of BIOx acceptors, the high *K*_eq_ values of the synthesized acceptors are not a requirement. To highlight the utility of these enhanced tM acceptors, their use in solution polymerization with bis-thiol monomers, where molecular weight is strongly controlled by *K*_eq_, was investigated. Thus, solutions of **3**_**R**_ and 2,2′-(ethylenedioxy)diethanethiol of varying concentrations ranging from 1 mM to 200 mM were prepared in dry DMSO and allowed to equilibrate overnight before solution viscometry measurements were carried out to follow the polymerization reaction (Figure 6a). The zero shear specific viscosities (η_sp_) were extrapolated from shear rate sweeps (see representative viscosity vs shear rate plot for **5**_**H**_, Figure S16) and were plotted against the concentration to reveal different regimes of the polymerization (Figure 6b). Log–log plots of η_sp_ vs concentration provide structural insights into the solution polymers, allowing for the determination of the critical polymerization concentrations (CPC) for the ditopic acceptors, where linear polymer formation begins to dominate over the formation of oligomeric cyclic structures.^46,55^ Here, the transition to a slope of ∼1.5 is indicative of the formation of linear chains, which all four BIOx crosslinkers were able to achieve. In agreement with the trend in their *K*_eq_ values, the CPCs of **5**_**Cl**_, **5**_**H**_, **5**_**Me**_, and **5**_**OMe**_ were found to be 78, 85, 86, and 95 mM (corresponding to extents of association of ∼99, 99, 98, and 93%), respectively. Additionally, it was also noted that **5**_**Cl**_ had the highest viscosity at 200 mM, with descending values moving to **5**_**OMe**_, implying higher overall degrees of polymerization. As expected, increasing the temperature was found to increase the CPC; in the case of **5**_**H**_ measured at 50 °C, the CPC was found to increase to 99 mM (Figure S17), corresponding to an expected extent of association of ∼96%.

To emphasize the importance of the enhanced *K*_eq_ values, this experiment was also performed using an analogous BCA crosslinker, which did not prove capable of forming linear polymers in the same concentration regime (max slope ∼0.5, Figure 6b).

## Conclusions

In summary, a new class of isoxazolone-based tM acceptors have been readily synthesized and shown to be a versatile new class of room-temperature, tunable, and catalyst-free dynamic motifs. This heterocyclic electrophile design takes advantage of aromatization upon thiol addition, resulting in an enhanced *K*_eq_ range up to ∼10^5^ M^–1^. Electronic manipulation of both the R_1_ and R_2_ positions results in predictable equilibrium constant changes, with R_2_ having a significantly stronger effect. Incorporating these acceptors into linear polymers and bulk networks allows for an impressive range of tunable mechanical properties that are accessible from a polymer containing room-temperature dynamic bonds. The high tunability of the tM acceptor combined with the ability to access high equilibrium constants offer a route to access mechanical robust stimuli-responsive materials that are adaptive at room temperature; such studies are the subject of current investigations and will be reported in due course.

## Supporting Information Available

Synthetic procedures, material characterization including NMR experiments, TGA, DSC, shear rheology, AFM, and viscometry measurements (PDF)
