# Supplementary material for: Enhancing the Equilibrium of Dynamic Thia-Michael Reactions through Heterocyclic Design
Source: J Am Chem Soc. 2023 Jun 23;145(26):14427–34. doi: 10.1021/jacs.3c03643 (PMC10326877; doi:10.1021/jacs.3c03643)
Supplement: Supplementary file 1 — ja3c03643_si_001.pdf [file ja3c03643_si_001.pdf]

Supporting Information

Enhancing the equilibrium of dynamic thia-Michael reactions through heterocyclic design

Alex E. Crolais[1,†], Neil D. Dolinski[2,†], Nicholas R. Boynton[2], Julia M. Radhakrishnan[1], Scott A. Snyder[1,*], Stuart J. Rowan[1,2*]

[1]Department of Chemistry, University of Chicago, Chicago, IL 60637, USA

[2]Pritzker School of Molecular Engineering, University of Chicago, Chicago, IL 60637, USA

Table of Contents

1. EXPERIMENTAL DETAILS **S3**
A. Materials S3
B. Instrumentation S3
C. Synthesis S5
D. Sample preparation S13
2. CHARACTERIZATION **S14**
A. NMR Studies S14
B. Thermal Properties S19
C. Atomic Force Microscopy S20
D. Reprocessing Studies S21
E. Stress Relaxation S23
F. Creep Measurements S25
G. Solution Viscometry S26
REFERENCES **S27**
SELECTED SPECTRA **S28**

1. EXPERIMENTAL DETAILS

1A. Materials

Methyl (4-chlorobenzoyl)acetate (>90%), hydroxylamine hydrochloride (98%), p-anisaldehyde (98%), p-tolualdehyde (97%), benzaldehyde (99%), triethylene glycol (99%), triphenylphosphine (Ph_3P, ≥99%), imidazole (99%), iodine (≥99.8%), sodium hydride (60% dispersion in mineral oil), diethyl carbonate (99%), piperidine (99%), and 2,2'-(ethylenedioxy)ditethanethiol (95%) were purchased from Sigma Aldrich. 3-(4-Methoxyphenyl)-3-oxo-propionic acid methyl ester (97%) was purchased from Oakwood Chemical. Ethyl benzoylacetate (>95%) and 4-chlorobenzaldehyde (>97%) were purchased from TCI Chemicals. Sodium acetate (≥99%) and potassium carbonate (≥99%) were purchased from Fisher Scientific. 4-hydroxyacetophenone (99%) was purchased from Alfa Aesar. All solvents were purchased from Fisher and all deuterated solvents were purchased from Oakwood. All chemicals were used as received unless otherwise noted. All dynamic tM films were tested within a week of preparation and stored below T_g prior to measurement.

1B. Instrumentation

Nuclear Magnetic Resonance (NMR) – NMR spectroscopy was performed using either a 400 MHz Bruker Avance III HD; 9.4 Tesla NMR or a 500 MHz Bruker Avance III HD; 11.7 Tesla NMR.

High-Resolution Mass Spectrometry (HRMS) – High-resolution mass spectra were recorded on Agilent 6244 Tof-MS using ESI (Electrospray Ionization) at the University of Chicago Mass Spectroscopy Core Facility.

Thermogravimetric Analysis (TGA) – Thermogravimetric analysis was performed using a TA Instruments Discovery Thermogravimetric Analyzer in the Soft Matter Characterization Facility at the University of Chicago. Tests were conducted using a ramp rate of 20 °C/min starting from operating temperature (ca. 30 °C) to 600 °C.

Differential Scanning Calorimetry (DSC) – DSC was performed using a TA Instruments Discovery 2500 Differential Scanning Calorimeter in the Soft Matter Characterization Facility at the University of Chicago. Samples were prepared in aluminum hermetic pans purchased from TA Instruments and were hermetically sealed. Dynamic networks underwent a heat-cool-heat cycle (190 °C/-80 °C/ 190 °C) run at 10 °C/min.

Shear Rheology – Rheology was performed using a TA Instruments ARES-G2 shear rheometer with Forced Convection oven (20 °C – 500 °C) attached to an Air Chiller System (-120 °C – 20 °C) and running TA Trios Software in the Soft Matter Characterization Facility at the University of Chicago. An 8 mm parallel plate was used for all tests.

Samples were loaded onto the plates, heated to 150 °C and pressed with an axial compression force of 0.01 N until geometry gap was stable. Samples were allowed to cool until excess material could be removed using a razor blade. Note: prolonged exposure to 150 °C conditions can result in the onset of irreversible side reactions that can impact mechanical properties, see Figure S9.

$\mathbf{3_R}$ samples were held at 150 °C for 1 minute before being cooled to 30 °C at a rate of 3 °C/min. Conditioning options were set to 0.01 N axial compression force and strain adjustments were

disabled. Axial compression force was changed to 1 N a few degrees higher than the samples T_g during the cooling sweep. Samples were then heated back to 150 °C, reverting the axial force back to 0.01 N at the transition temperature. The first cooling was used for reported measurements but were in close agreement with the heating sweep.

Stress Relaxation – For **4**$_{\mathbf{Cl}}$, stress relaxation experiments were performed at various temperatures within its rubbery plateau region (120, 125, 130, 135, 140, 145, and 150 °C). The sample was loaded in the same way as described in the temperature sweep experiment. Conditioning options were set to 6 N axial compression force and strain adjustments were disabled. A 3% strain was then applied, and the stress was monitored over 2 hours for each temperature.

Creep Testing – Creep tests were performed using a TA Instruments Discovery HR-30 shear rheometer in the Soft Matter Characterization Facility at the University of Chicago. Measurements were taken at three temperatures (90, 95, and 100 °C). Samples were loaded onto the rheometer at 150 °C and then cooled to their testing temperature. Excess material was removed with a razor blade. Axial compression force was set to 1 N and 3000 Pa of shear stress was applied to the sample.

Solution Viscometry – Solution viscometry was performed using an Anton Paar MCR301 rheometer with a 2 degree 25 mm cone-plate geometry using a gap size of 0.106 mm.

For each ditopic tM crosslinker (**3**$_{\mathbf{R}}$), a 200 mM solution in DMSO treated with molecular sieves was prepared in a 1 dram glass vial to which 2,2′-(ethylenedioxy)diethanethiol (1 equiv.) was added. A sequence of serial dilutions was then performed to make solutions at 150, 100, 50, 25, 12.5, 10, 6, 3, and 1 mM. Solutions were then allowed to equilibrate overnight. Using a 2 degree 25 mm cone-plate geometry, viscosity was measured across a sweep of shear rates (0.1 to 500 1/s). All samples were found to have a nearly flat response, allowing for extrapolation to zero-shear viscosity.

Atomic Force Microscopy (AFM) – AFM measurements were taken using an Asylum Research Cypher ES AFM with BlueDrive in the University of Chicago's Materials Preparation and Measurement Laboratory as part of the NSF Materials Research Science and Engineering Center (MRSEC). Scans were conducted in tapping mode using a NanoWorld Arrow™ Ultra High Frequency (UHF) AFM probe.

1C. Synthesis

General Procedure A: *Synthesis of Isoxazolones*

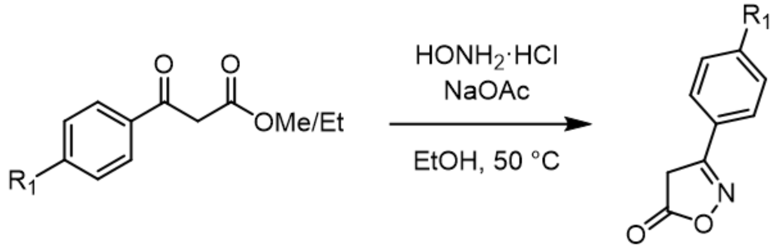

$HONH_2$·HCl (2 equiv.) and NaOAc (2 equiv.) were added to a round bottom flash charged with EtOH and allowed to stir for 5 minutes. After, the β-ketoester (1 equiv.) was added. The reaction was heated to 50 °C and allowed to stir overnight. The solution was then concentrated under reduced pressure and dissolved in CH_2Cl_2. The organic phase was then washed with a concentrated brine solution. The aqueous layer was then extracted with CH_2Cl_2 (x2) and the combined organic phases were dried using Na_2SO_4, filtered and concentrated under reduced pressure. The product was used in the following step without further purification.[1]

3-(4-methoxyphenyl)isoxazole-5(4H)-one

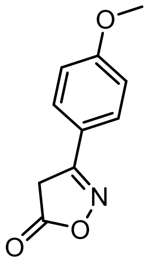

3-(4-methoxyphenyl)isoxazol-5(4H)-one was prepared according to procedure **A.** 150 mg of ketoester was used and 119 mg of product was obtained as a pale pink solid (92% yield). Spectral data matched reported literature data.[2] ^{1}H NMR (400 MHz, $CDCl_3$) δ 7.63 – 7.59 (m, 2H), 7.00 – 6.95 (m, 2H), 3.87 (s, 3H), 3.78 (s, 2H).

3-phenylisoxazol-5(4H)-one

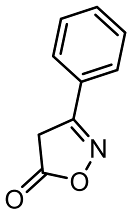

3-phenylisoxazol-5(4H)-one was prepared according to procedure **A.** 150 mg of ketoester was used and 125 mg of product was obtained as a pale pink solid (99% yield). Spectral data matched reported literature data.[3] ^{1}H NMR (400 MHz, $CDCl_3$) δ 7.72 – 7.65 (m, 2H), 7.57 – 7.45 (m, 3H), 3.81 (s, 2H).

3-(4-chlorophenyl)isoxazole-5(4H)-one

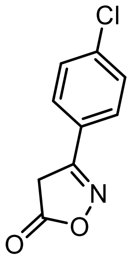

3-(4-chlorophenyl)isoxazol-5(4H)-one was prepared according to procedure **A.** 5 g of ketoester was used and 4.27 g of product was obtained as a pale pink solid (93% yield). Spectral data matched reported literature data.[4] ^{1}H NMR (500 MHz, $CDCl_3$) δ 7.66 – 7.59 (m, 2H), 7.50 – 7.43 (m, 2H), 3.79 (s, 2H).

General Procedure B: *Knoevenagel Condensations*

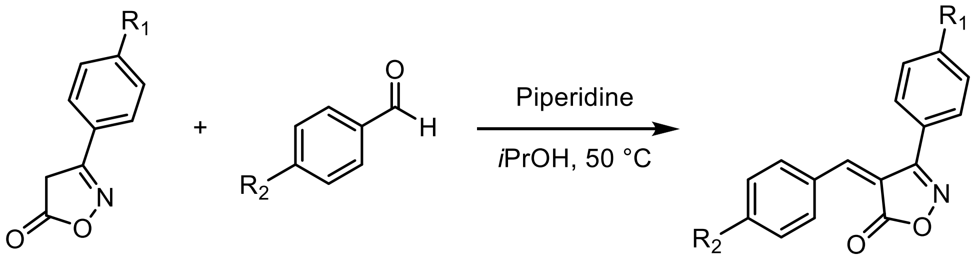

A round bottom was charged with isoxazolone (1 equiv.), aldehyde (1.2 equiv.) and *i*PrOH (0.5 M in respect to isoxazolone) and stirred. Then, piperidine (5 µL/mmol isoxazolone) was added. The solution was heated to 50 °C and monitored by TLC until completion (*ca.* 3 – 5 hr). Products precipitated out as a bright to pale yellow solid and were isolated by filtration and washed with a cold 1:1 *i*PrOH/H_2O mixture.[1]

(Z)-4-(4-methoxybenzylidene)-3-(4-methoxyphenyl)isoxazol-5(4H)-one ($\mathbf{1}_{\mathbf{OMe,OMe}}$)

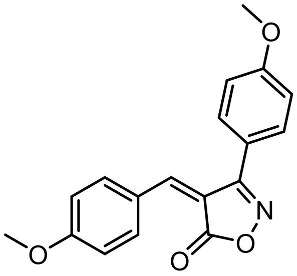

$\mathbf{1}_{\mathbf{OMe,OMe}}$ was prepared according to general procedure **B**. 150 mg of isoxazolone was used and 218 mg of product was obtained as a yellow solid (90% yield). ^{1}H NMR (500 MHz, CDCl3) δ 8.44 – 8.37 (m, 2H), 7.54 (s, 1H), 7.52 (d, J = 3.3 Hz, 2H), 7.10 – 7.04 (m, 2H), 7.03 – 6.97 (m, 2H), 3.92 (s, 3H), 3.89 (s, 3H).^{13}C NMR (101 MHz, $CDCl_3$) δ 169.20, 164.84, 164.05, 161.74, 152.11, 137.30, 130.32, 126.10, 120.09, 115.79, 114.82, 114.73, 55.85, 55.60. HRMS (ESI) calcd for $C_{18}H_{15}NO_4$ $[M + Na]^+$ *m/z* 332.0893, found 332.0890.

(Z)-4-(4-methylbenzylidene)-3-(4-methoxyphenyl)isoxazol-5(4H)-one ($\mathbf{1}_{\mathbf{OMe,Me}}$)

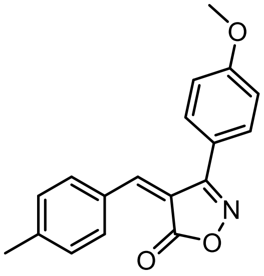

$\mathbf{1}_{\mathbf{OMe,Me}}$ was prepared according to general procedure **B**. 150 mg of isoxazolone was used and 186 mg of product was obtained as a yellow solid (81% yield). ^{1}H NMR (500 MHz, CDCl3) δ 8.24 (d, J = 8.1 Hz, 2H), 7.57 (s, 1H), 7.55 – 7.51 (m, 2H), 7.31 (d, J = 8.0 Hz, 2H), 7.09 – 7.04 (m, 2H), 3.89 (d, J = 0.9 Hz, 3H), 2.44 (s, 3H). ^{13}C NMR (101 MHz, $CDCl_3$) δ 168.60, 163.84, 161.82, 152.73, 145.93, 134.40, 130.29, 130.17, 129.93, 119.82, 117.89, 114.85, 55.60, 22.20. HRMS (ESI) calcd for $C_{18}H_{15}NO_4$ $[M + Na]^+$ *m/z* 316.0944, found 316.0942.

(Z)-4-benzylidene-3-(4-methoxyphenyl)isoxazol-5(4H)-one ($\mathbf{1}_{\mathbf{OMe,H}}$)

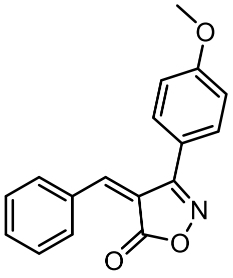

$\mathbf{1}_{\mathbf{OMe,H}}$ was prepared according to general procedure **B**. 150 mg of isoxazolone was used and 170 mg of product was obtained as a yellow solid (78% yield). Spectral data matched reported literature data.[1] ^{1}H NMR (400 MHz, $CDCl_3$) δ 8.36 – 8.28 (m, 2H), 7.64 – 7.47 (m, 6H), 7.12 – 7.04 (m, 2H), 3.90 (s, 3H).

(Z)-4-(4-chlorobenzylidene)-3-(4-methoxyphenyl)isoxazol-5(4H)-one (**1**$_{\mathbf{OMe,Cl}}$)

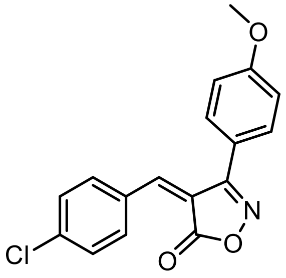

1$_{\mathbf{OMe,Cl}}$ was prepared according to general procedure **B**. 150 mg of isoxazolone was used and 134 mg of product was obtained as a yellow solid (55% yield). ^{1}H NMR (500 MHz, $CDCl_3$) δ 8.28 (d, *J* = 8.6 Hz, 2H), 7.56 – 7.51 (m, 3H), 7.48 (d, *J* = 8.7 Hz, 2H), 7.08 (d, *J* = 8.8 Hz, 2H), 3.90 (s, 4H). ^{13}C NMR (101 MHz, $CDCl_3$) δ 168.23, 163.59, 162.00, 150.86, 140.66, 135.25, 130.95, 130.28, 129.49, 119.64, 119.47, 114.97, 55.64. HRMS (ESI) calcd for $C_{18}H_{15}NO_4$ $[M + H]^+$ *m/z* 314.0578, found 314.0631.

(Z)-4-(4-methoxybenzylidene)-3-phenylisoxazol-5(4H)-one (**1**$_{\mathbf{H,OMe}}$)

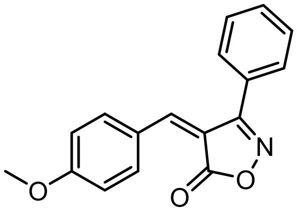

1$_{\mathbf{H,OMe}}$ was prepared according to general procedure **B**. 50 mg of isoxazolone was used and 41 mg of product was obtained as a yellow solid (47% yield). Spectral data matched reported literature data.[5] ^{1}H NMR (400 MHz, $CDCl_3$) δ 8.46 – 8.38 (m, 2H), 7.64 – 7.48 (m, 6H), 7.04 – 6.96 (m, 2H), 3.92 (s, 3H).

(Z)-4-(4-methylbenzylidene)-3-phenylisoxazol-5(4H)-one (**1**$_{\mathbf{H,Me}}$)

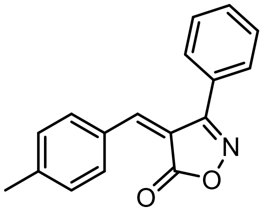

1$_{\mathbf{H,Me}}$ was prepared according to general procedure **B**. 50 mg of isoxazolone was used and 48 mg of product was obtained as a yellow solid (58% yield). Spectral data matched reported literature data.[6] ^{1}H NMR (400 MHz, $CDCl_3$) δ 8.30 – 8.22 (m, 2H), 7.65 – 7.52 (m, 6H), 7.36 – 7.28 (m, 2H), 2.45 (s, 3H).

(Z)-4-benzylidene-3-phenylisoxazol-5(4H)-one (**1**$_{\mathbf{H,H}}$)

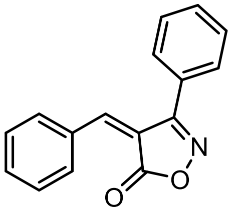

1$_{\mathbf{H,H}}$ was prepared according to general procedure **B**. 50 mg of isoxazolone was used and 33 mg of product obtained as a yellow solid (42% yield). Spectral data matched reported literature data.[1] ^{1}H NMR (400 MHz, $CDCl_3$) δ 8.37 – 8.29 (m, 2H), 7.65 – 7.48 (m, 9H).

(Z)-4-(4-chlorobenzylidene)-3-phenylisoxazol-5(4H)-one (**1**$_{\mathbf{H,Cl}}$)

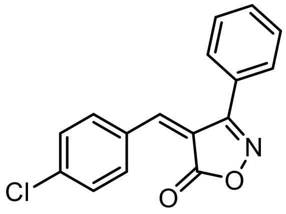

(**1**$_{\mathbf{H,Cl}}$) was prepared according to general procedure **B**. 150 mg of isoxazolone was used and 106 mg of product obtained as a yellow solid (40% yield). Spectral data matched reported literature data.[1] ^{1}H NMR (400 MHz, $CDCl_3$) δ 8.33 – 8.25 (m, 2H), 7.66 – 7.42 (m, 8H).

(Z)-4-(4-methoxybenzylidene)-3-(4-chlorophenyl)isoxazol-5(4H)-one (**1$_{\mathbf{Cl,OMe}}$**)

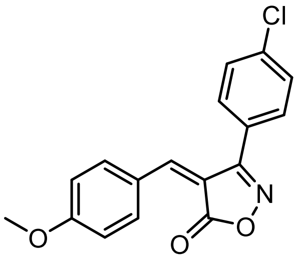

1$_{\mathbf{Cl,OMe}}$ was prepared according to general procedure **B**. 50 mg of isoxazolone was used and 59 mg of product obtained as a yellow solid (51% yield). ^{1}H NMR (400 MHz, $CDCl_3$) δ 8.45 – 8.38 (m, 2H), 7.54 (s, 4H), 7.47 (s, 1H), 7.05 – 6.98 (m, 2H), 3.93 (s, 3H). ^{13}C NMR (101 MHz, $CDCl_3$) δ 168.93, 165.17, 163.54, 152.13, 137.51, 137.33, 130.23, 129.74, 126.38, 126.00, 115.20, 114.87, 55.92. HRMS (ESI) calcd for $C_{18}H_{15}NO_4$ [M + H]$^+$ *m/z* 314.0578, found 314.0583.

(Z)-4-(4-methylbenzylidene)-3-(4-chlorophenyl)isoxazol-5(4H)-one (**1$_{\mathbf{Cl,Me}}$**)

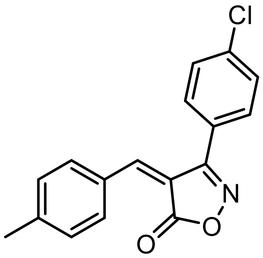

1$_{\mathbf{Cl,Me}}$ was prepared according to general procedure **B**. 100 mg of isoxazolone was used and 149 mg of product obtained as a yellow solid (60% yield). ^{1}H NMR (400 MHz, $CDCl_3$) δ 8.29 – 8.21 (m, 2H), 7.54 (m, 5H), 7.32 (d, *J* = 8.1 Hz, 2H), 2.45 (s, 3H). ^{13}C NMR (101 MHz, $CDCl_3$) δ 168.29, 163.34, 152.82, 146.41, 137.46, 134.55, 130.18, 130.05, 129.77, 126.11, 117.33, 22.26. HRMS (ESI) calcd for $C_{18}H_{15}NO_4$ [M + H]$^+$ *m/z* 298.0629, found 298.0654.

(Z)-4-benzylidene-3-(4-chlorophenyl)isoxazol-5(4H)-one (**1$_{\mathbf{Cl,H}}$**)

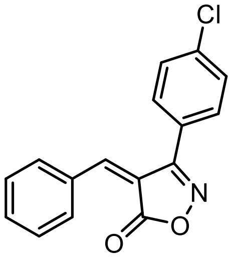

1$_{\mathbf{Cl,H}}$ was prepared according to general procedure **B**. 150 mg of isoxazolone was used and 356 mg of product was obtained as a yellow solid (89% yield). ^{1}H NMR (500 MHz, $CDCl_3$) δ 8.34 – 8.29 (m, 2H), 7.63 – 7.49 (m, 8H). ^{13}C NMR (101 MHz, $CDCl_3$) δ 167.92, 163.20, 152.85, 137.58, 134.53, 134.18, 132.37, 130.16, 129.82, 129.18, 125.92, 118.60. HRMS (ESI) calcd for $C_{18}H_{15}NO_4$ [M + H]$^+$ *m/z* 284.0473, found 284.0469.

(Z)-4-(4-chlorobenzylidene)-3-(4-chlorophenyl)isoxazol-5(4H)-one (**1$_{\mathbf{Cl,Cl}}$**)

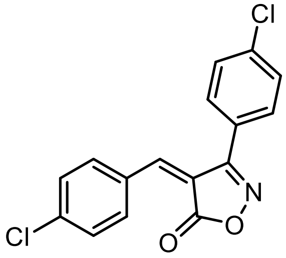

1$_{\mathbf{Cl,Cl}}$ was prepared according to general procedure **B**. 196 mg of isoxazolone was used and 80 mg of product was obtained as a yellow solid (25% yield). ^{1}H NMR (500 MHz, $CDCl_3$) δ 8.28 (d, *J* = 8.6 Hz, 2H), 7.55 (mf, 4H), 7.52 – 7.46 (m, 3H). ^{13}C NMR (101 MHz, $CDCl_3$) δ 167.88, 163.08, 151.00, 141.05, 137.68, 135.35, 130.77, 130.12, 129.86, 129.57, 125.70, 118.97. HRMS (ESI) calcd for $C_{18}H_{15}NO_4$ [M + H]$^+$ *m/z* 318.0083, found 318.0089.

Methyl-(E)-2-cyano-3-phenylacrylate (**BCA-H**)

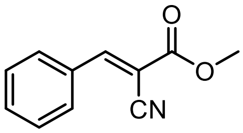

BCA-H was prepared according to a previously reported procedure.[7]

Synthesis of Ditopic Michael Acceptors

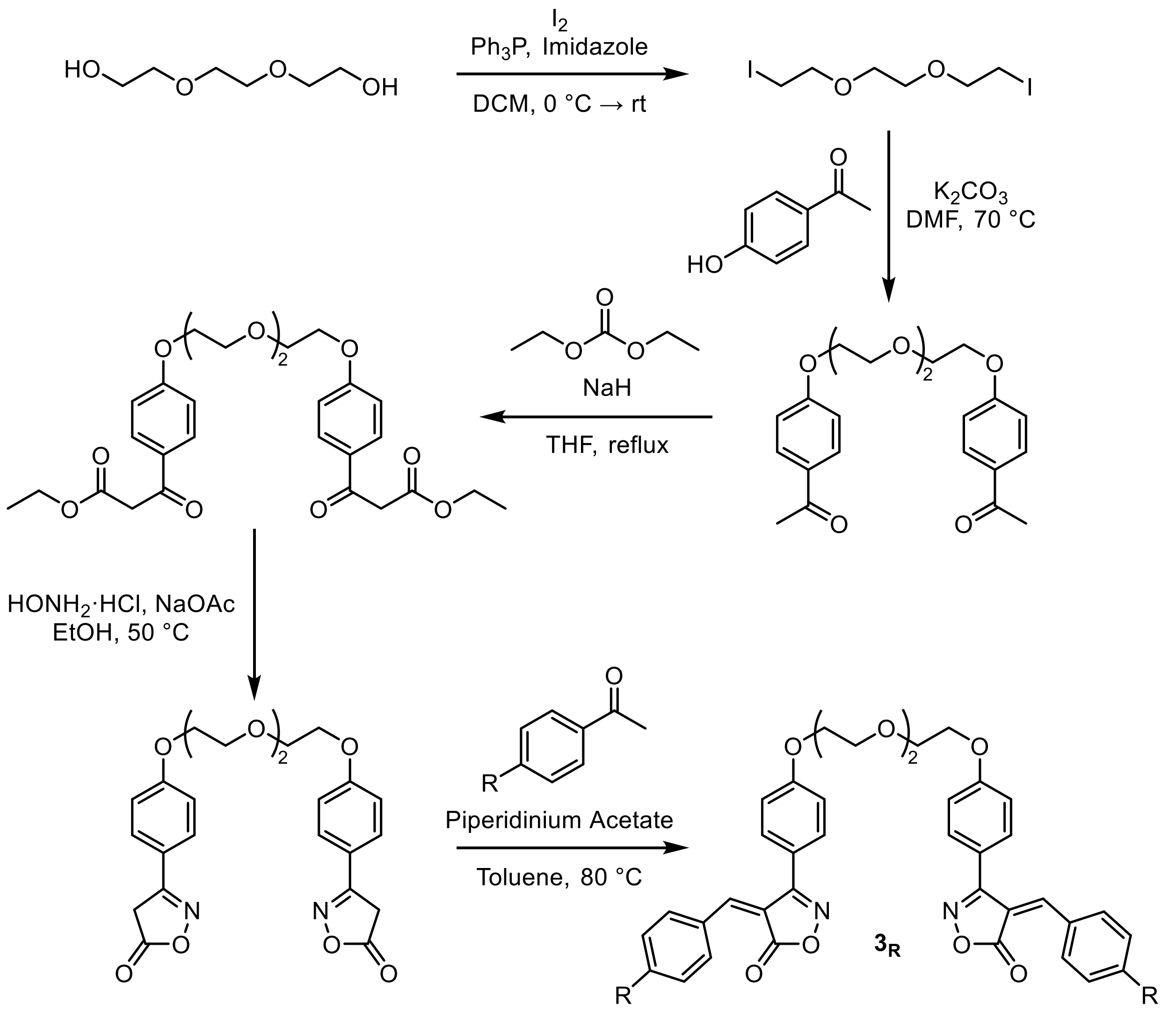

Synthesis of 1,2-bis(2-iodoethoxy)ethane:

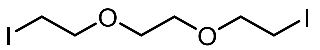

2,2'-(ethylenedioxy)diethanol (1 equiv.), triphenylphosphine (2.4 equiv) and imidazole (3 equiv.) were added to a round bottom flask charged with anhydrous CH_2Cl_2. The solution was then cooled to 0 °C, after which I_2 (2.6 equiv.) was added slowly. The reaction was stirred overnight at room temperature then quenched with sat. $Na_2S_2O_3$ solution. The organic layer was separated and the aqueous layer was extracted with CH_2Cl_2 (3x). The combined organic layers were washed with brine, dried over Na_2SO_4 and filtered, then concentrated under reduced pressure. The crude product was then purified using silica gel column chromatography (10:1 hexanes/EtOAc). 15 g of 2,2'-(ethylenedioxy)diethanol was used and 34.9 g of 1,2-bis(2-iodoethoxy)ethane was obtained as a yellow oil (95% yield). Spectral data matched reported literature data.[8] ^{1}H NMR (500 MHz, $CDCl_3$) δ 3.77 (t, J = 6.8 Hz, 4H), 3.67 (s, 4H), 3.27 (t, J = 6.8 Hz, 4H).

Synthesis of triethylene glycol bis(acetophenone)

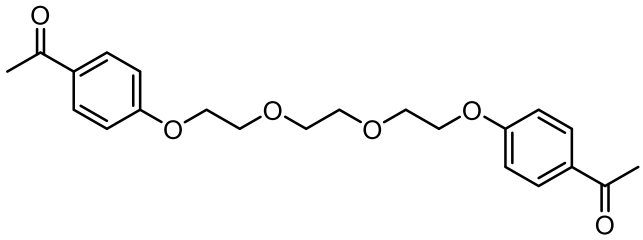

To a round bottom charged with DMF, the 1,2-bis(2-iodoethoxy)ethane (1equiv.), 4-hydroxyacetophenone (3 equiv.) and K_2CO_3 (5 equiv.) were added and allowed to stir overnight at 70 °C. After cooling to room temperature, EtOAc was added, and the organic phase was washed with 1 M NaOH solution followed by brine. The organic layer was then dried over Na_2SO_4 and filtered, the concentrated under reduced pressure. The crude product was then purified using silica gel column chromatography (4:1 → 1:1 hexanes/EtOAc). 34.9 g of 1,2-bis(2-iodoethoxy)ethane was used and 34.5 g of the product was obtained as a white solid (94% yield). Spectral data matched reported literature data.[9] ^{1}H NMR (400 MHz, $CDCl_3$) δ 7.94 – 7.88 (m, 4H), 6.97 – 6.89 (m, 4H), 4.18 (dd, J = 5.7, 3.9 Hz, 4H), 3.88 (dd, J = 5.7, 3.9 Hz, 4H), 3.76 (s, 4H), 2.54 (s, 6H)

Synthesis of diethyl 3,3'-((((ethane-1,2-diylbis(oxy))bis(ethane-2,1-diyl))bis(oxy))bis(4,1-phenylene))bis(3-oxopropanoate)

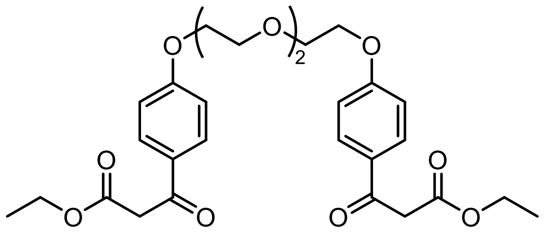

NaH (4 equiv.) was added to a round bottom flask charged with anhydrous THF (0.1 M in respect to triethylene glycol bis(acetophenone)). After, diethyl carbonate (4 equiv.) was added followed by the addition of triethylene glycol bis(acetophenone) (1 equiv.). The reaction was then refluxed until full conversion based on TLC. After, the reaction was cooled to 0 °C and quenched with 2 M aqueous HCl. The mixture was then extracted three times with EtOAc. The combined organic layers were then washed with sat. $NaHCO_3$ followed by a sat. brine solution. The organic layer was then dried over Na_2SO_4 and filtered, the concentrated under reduced pressure. The crude product was then purified using silica gel column chromatography (1:1 hexanes/EtOAc). 500 mg of triethylene glycol bis(acetophenone) was used and 559.3 mg of the product was obtained as a yellow oil (82% yield).[10] ^{1}H NMR (500 MHz, $CDCl_3$) δ 7.93 – 7.86 (m, 4H), 6.93 (dd, J = 9.4, 2.6 Hz, 4H), 4.22 – 4.14 (m, 8H), 3.92 (s, 4H), 3.89 – 3.85 (m, 4H), 3.74 (s, 4H), 1.24 (t, J = 7.1 Hz, 6H). ^{13}C NMR (101 MHz, $CDCl_3$) δ 191.09, 167.84, 163.29, 130.98, 129.37, 114.59, 71.03, 69.65, 67.77, 61.51, 45.91, 14.20. **Peaks selected are of ketoester tautomer, NMR contain enol tautomer as well* HRMS (ESI) calcd for $C_{28}H_{34}O_{10}$ $[M + H]^+$ m/z 531.2225, found 531.2264.

Synthesis of 3,3'-((((ethane-1,2-diylbis(oxy))bis(ethane-2,1-diyl))bis(oxy))bis(4,1-phenylene))bis(isoxazol-5(4H)-one)

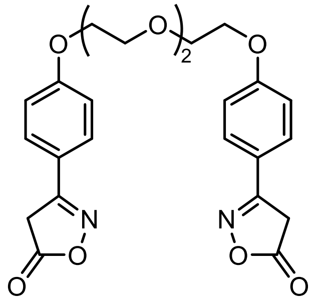

$HONH_2 \cdot HCl$ (4 equiv.) and NaOAc (4 equiv.) were added to a round bottom flask charged with EtOH (0.1 M in respect to di-ketoester) and allowed to stir for 5 minutes. After, di-ketoester (1 equiv.) was added and the mixture was heated to 50 °C and allowed to stir overnight. Upon completion, EtOH was removed under reduced pressure and the crude product was dissolved in CH_2Cl_2. The organic layer was then washed with sat. brine (3x). The organic layer was then dried over Na_2SO_4 and filtered, the concentrated under reduced pressure. 50 mg of the di-ketoester was used and 42.0 mg of product as a pale

pink solid (95% yield) was obtained and used in subsequent steps without further purification. ^{1}H NMR (400 MHz, $CDCl_3$) δ 7.63 – 7.55 (m, 4H), 7.01 – 6.93 (m, 4H), 4.21 – 4.14 (m, 4H), 3.92 – 3.85 (m, 4H), 3.76 (d, *J* = 2.7 Hz, 8H). ^{13}C NMR (101 MHz, $CDCl_3$) δ 175.03, 162.59, 161.97, 128.43, 120.40, 115.36, 71.09, 69.75, 67.81, 34.26. HRMS (ESI) calcd for $C_{24}H_{24}N_2O_8$ $[M + H]^+$ *m/z* 469.1605, found 469.1608.

General Procedure C: *Synthesis of Ditopic Michael Acceptor*
A round bottom was charged with diisoxazolone (1 equiv.), aldehyde (3 equiv.) and toluene (0.01 M in respect to diisoxazolone) and allowed to stir. Then, piperidinium acetate was added. The solution was heated to 80 °C and closely monitored by TLC until completion. The reaction mixture was then concentrated under reduced pressure and purified using silica gel column chromatography (4:1 hexanes/EtOAc → 100% EtOAc).

(Z)-3,3'-((((ethane-1,2-diylbis(oxy))bis(ethane-2,1-diyl))bis(oxy))bis(4,1-phenylene))bis(4-((Z)-4-methoxybenzylidene)isoxazol-5(4H)-one) (**3**$_{\mathbf{OMe}}$)

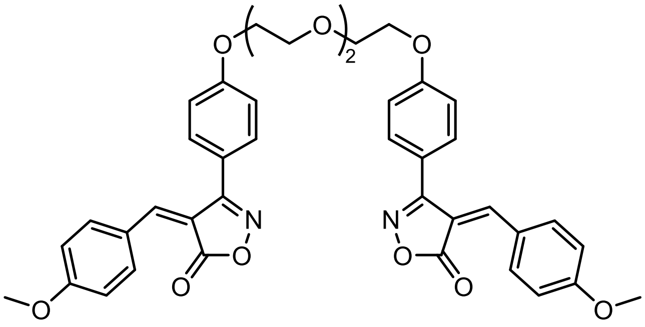

3$_{\mathbf{OMe}}$ was prepared according to general procedure **C**. 150 mg of di-isoxazolone was used and 192 mg of product was obtained as a yellow solid (85% yield). ^{1}H NMR (400 MHz, $CDCl_3$) δ 8.39 (d, *J* = 9.0 Hz, 4H), 7.54 – 7.47 (m, 6H), 7.08 (d, *J* = 8.7 Hz, 4H), 6.99 (d, *J* = 9.0 Hz, 4H), 4.22 (t, 4.6 Hz, 4H), 3.96 – 3.87 (m, 10H), 3.79 (s, 4H). ^{13}C NMR (101 MHz, $CDCl_3$) δ 169.18, 164.88, 164.00, 160.97, 152.10, 137.32, 130.32, 126.08, 120.34, 115.75, 115.47, 114.76, 71.12, 69.86, 67.81, 55.87. HRMS (ESI) calcd for $C_{40}H_{36}N_2O_{10}$ $[M + H]^+$ *m/z* 705.2443, found 705.2459.

(Z)-3,3'-((((ethane-1,2-diylbis(oxy))bis(ethane-2,1-diyl))bis(oxy))bis(4,1-phenylene))bis(4-((Z)-4-methylbenzylidene)isoxazol-5(4H)-one) (**3**$_{\mathbf{Me}}$)

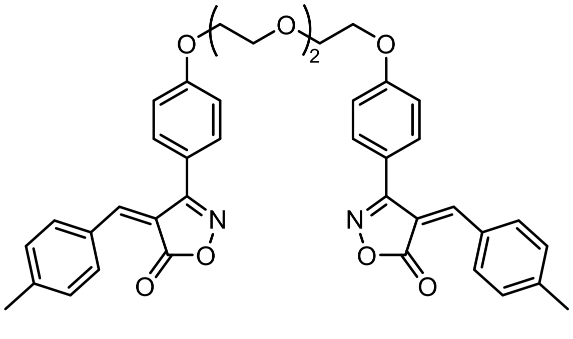

3$_{\mathbf{Me}}$ was prepared according to general procedure **C**. 150 mg of di-isoxazolone was used and 175 mg of product was obtained as a yellow solid (81% yield). ^{1}H NMR (400 MHz, $CDCl_3$) δ 8.23 (d, *J* = 8.3 Hz, 4H), 7.58 – 7.47 (m, 6H), 7.30 (d, *J* = 8.2 Hz, 4H), 7.08 (d, *J* = 8.8 Hz, 4H), 4.22 (dd, *J* = 5.8, 3.8 Hz, 4H), 3.92 (dd, *J* = 5.6, 3.9 Hz, 4H), 3.79 (s, 4H), 2.44 (s, 6H). ^{13}C NMR (101 MHz, $CDCl_3$) δ 168.57, 163.79, 161.06, 152.72, 145.97, 134.41, 130.29, 129.95, 129.31, 120.07, 117.88, 115.50, 71.10, 69.83, 67.81, 22.21. HRMS (ESI) calcd for $C_{40}H_{36}N_2O_8$ $[M + H]^+$ *m/z* 673.2544, found 673.2548.

(Z)-3,3'-((((ethane-1,2-diylbis(oxy))bis(ethane-2,1-diyl))bis(oxy))bis(4,1-phenylene))bis(4-((Z)-benzylidene)isoxazol-5(4H)-one) (**3**$_{\mathbf{H}}$)

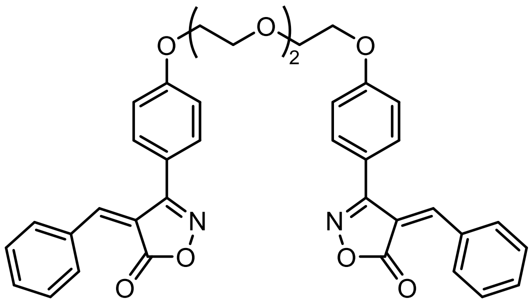

3$_{\mathbf{H}}$ was prepared according to general procedure **C**. 150 mg of di-isoxazolone was used and 144 mg of product was obtained as a yellow solid (70% yield). ^{1}H NMR (400 MHz, $CDCl_3$) δ 8.30 (dd, *J* = 8.2, 1.3 Hz, 4H), 7.63 – 7.46 (m, 12H), 7.09 (d, *J* = 8.8 Hz, 3H), 4.25 – 4.19 (m, 4H), 3.96 – 3.90 (m, 4H), 3.80 (s, 3H). ^{13}C NMR (101 MHz, $CDCl_3$) δ 168.23, 163.66, 161.14, 152.75, 134.21, 134.06, 132.50, 130.29, 129.11, 119.89, 119.15, 115.55, 71.11, 69.83, 67.82. HRMS (ESI) calcd for $C_{38}H_{32}N_2O_8$ [M + H]$^+$ *m/z* 645.2231, found 645.2237.

(Z)-3,3'-((((ethane-1,2-diylbis(oxy))bis(ethane-2,1-diyl))bis(oxy))bis(4,1-phenylene))bis(4-((Z)-4-chlorobenzylidene)isoxazol-5(4H)-one) (**3**$_{\mathbf{Cl}}$)

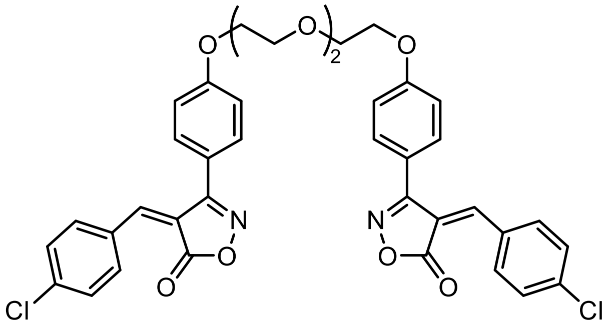

3$_{\mathbf{Cl}}$ was prepared according to general procedure **C**. 150 mg of di-isoxazolone was used and 154 mg of product was obtained as a yellow solid (67% yield). ^{1}H NMR (500 MHz, $CDCl_3$) δ 8.30 – 8.24 (m, 4H), 7.55 – 7.44 (m, 10H), 7.12 – 7.05 (m, 4H), 4.22 (dd, *J* = 5.7, 3.8 Hz, 4H), 3.95 – 3.87 (m, 4H), 3.79 (s, 4H). ^{13}C NMR (101 MHz, $CDCl_3$) δ 168.19, 163.54, 161.21, 150.88, 140.71, 135.26, 130.92, 130.27, 129.51, 119.71, 119.57, 115.60, 71.11, 69.84, 67.83. HRMS (ESI) calcd for $C_{38}H_{30}Cl_2N_2O_8$ [M + H]$^+$ *m/z* 713.1452, found 713.1433.

Triethylene glycol bis(benzalcyanoacetate) (3$_{\mathbf{BCA\text{-}H}}$)

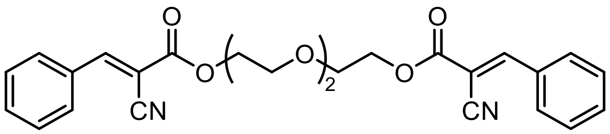

3$_{\mathbf{BCA\text{-}H}}$ was prepared according to a previously reported procedure.[7]

1D. Sample Preparation

Casting / Drying Bulk Dynamic Network Films – In a 20 mL glass vial, $\mathbf{3_R}$ was dissolved in chloroform (~1 mL per 50 mg $\mathbf{3_R}$). After, trimethylolpropane tris(3-mercaptopropionate) (2/3 equiv.) was added and the solution was shaken. The solution was then transferred to a Teflon® dish and covered with perforated aluminum foil. The sample was then air dried for 24 hours, dried in a vacuum oven at 50 °C for another 24 hours, and then dried at 150 °C for an additional thirty minutes.

Melt Pressing – To prepare / reprocess films, $\mathbf{4_R}$ samples were melt pressed at 5 tons of force for 15 minutes. $\mathbf{4_{OMe}}$ and $\mathbf{4_{Me}}$ were pressed at 100 °C, while $\mathbf{4_H}$ and $\mathbf{4_{Cl}}$ were pressed at 140 °C.

Dynamic Network AFM Samples – From the solution made in preparation of the bulk dynamic network films, ~10 µL was taken and drop-cast onto 12 mm round glass coverslips (**Note**: sample solution taken from glass vial prior to transferring to the Teflon® dish). After allowing to dry for 1 minute, the sample was transferred into a petri dish with a cracked lid and allowed to air dry for 24 hours. The sample was then dried in a vacuum oven at 50 °C for another 24 hours, and then dried at 150 °C for an additional thirty minutes.

2. CHARACTERIZATION

2A. NMR Studies

Dynamic Exchange Experiment of $\mathbf{1_{OMe,Cl}}$ – A stock solution (~1.5 mL) containing 50 mM each of $\mathbf{1_{OMe,Cl}}$ and 1-octanethiol prepared in DMSO-d_6 and was allowed to equilibrate at room temperature for ~24 hours. After measuring the extent of bond formation via ^{1}H NMR, 50 mM of benzyl mercaptan was added. After the addition of benzyl mercaptan, the sample was regularly monitored via NMR.

Competition Equilibrium Experiments of $\mathbf{1_{R1,R2}}$ and 1-octanethiol – Individual stock solutions (75 mM) of $\mathbf{1_{R1,R2}}$, 1-octanethiol, and competitor (mono-H when R_2 = -OMe or -Me and $\mathbf{1_{OMe,Me}}$ when R_2 = -H or -Cl) were prepared in DMSO-d_6 that was treated with molecular sieves. Equal volumes of each solution were added into a 1-dram vial (resulting in 25 mM concentrations of all species) and allowed to equilibrate overnight. Samples were then transferred to a NMR tube and ^{1}H spectra were taken. Representative peaks of interest and governing equations for determining the equilibrium constant are shown below in Figures S1–S2 and Equations S1–S2.

a.

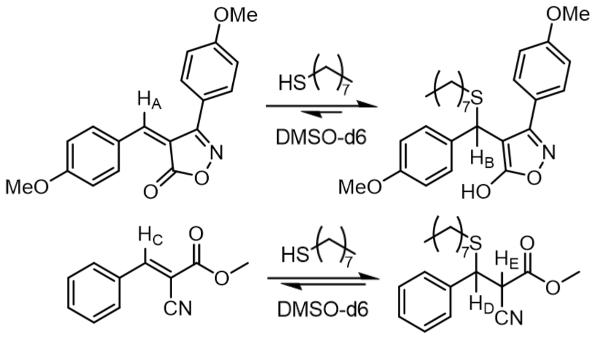

b.

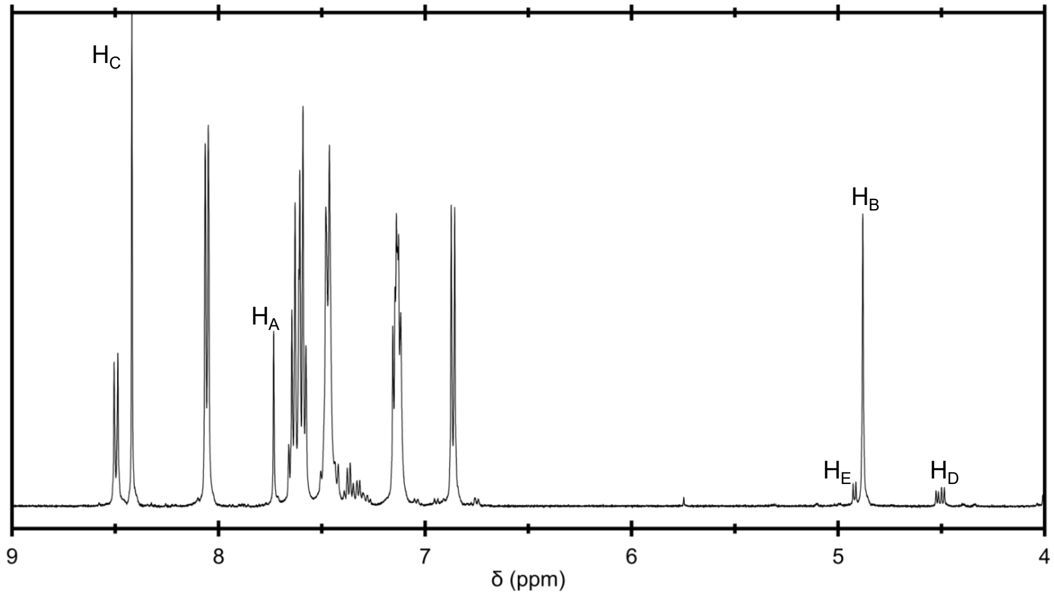

Figure S1. (a) Relevant equilibria in competition experiment between **$1_{OMe,OMe}$** and competitor **BCA-H** tM acceptors and (b) ^{1}H NMR of region of interest after overnight equilibration. (Note – the integration of peak H_D needs to be subtracted from the integration of peak H_B due to overlap with peak H_E)

$$K_{eq} = \frac{(\int(H_B+H_E)-\int H_D)*\int H_C}{\int H_A*\int H_D} * K_{eq,BCA-H} \qquad \text{(Eq. S1)}$$

Equation S1. Equation used to determine equilibrium constants from competition experiments using **BCA-H** as the competitor. (Note – the integration of peak H_D needs to be subtracted from the integration of peak H_B due to overlap with peak H_E)

a.

b.

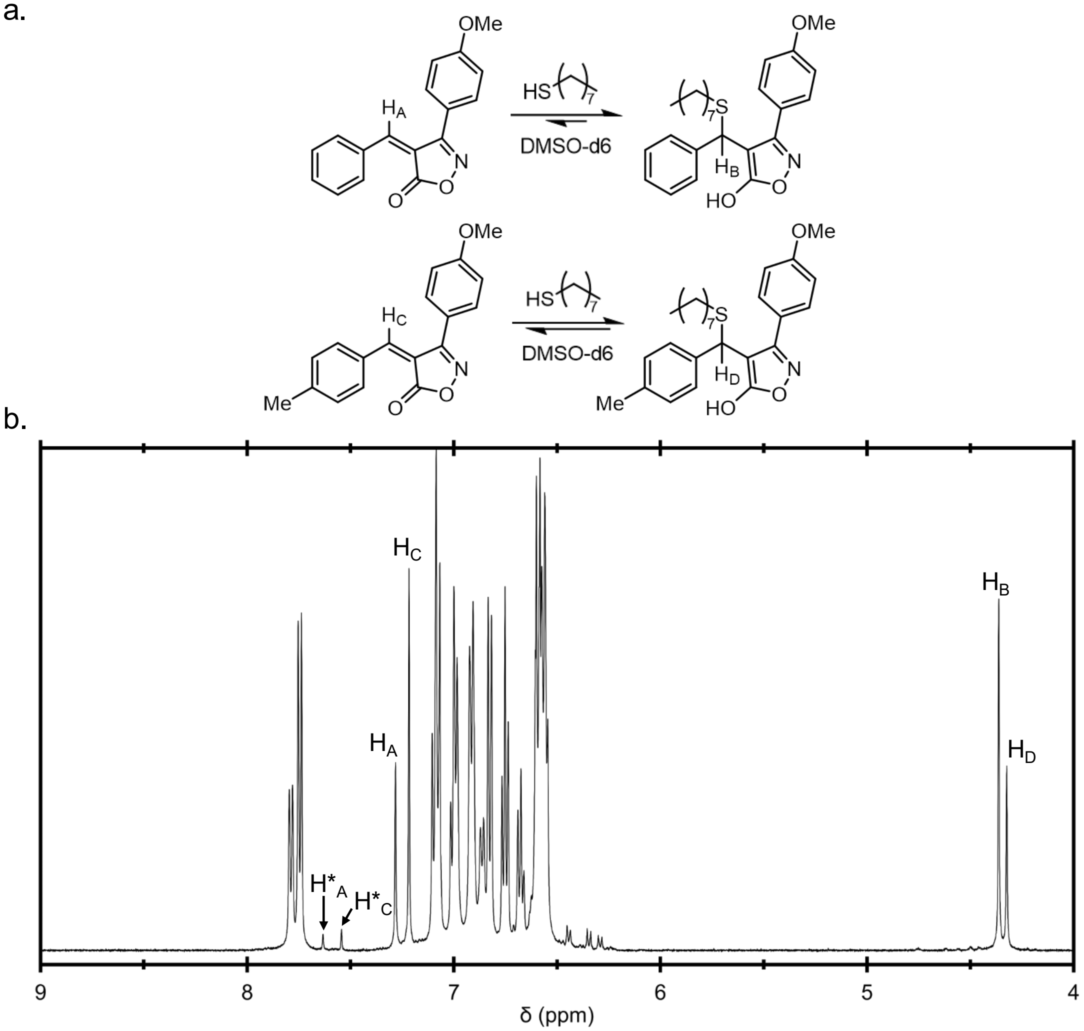

Figure S2. (a) Relevant equilibria in competition experiment between $\mathbf{1}_{\mathbf{OMe,H}}$ and competitor $\mathbf{1}_{\mathbf{OMe,Me}}$ tM acceptors and (b) ^{1}H NMR of region of interest after overnight equilibration.

$$K_{eq} = \frac{\int H_B * (\int H_C + \int H_C^*)}{(\int H_A + \int H_A^*) * \int H_D} * K_{eq,1OMe,Me} \qquad \text{(Eq. S2)}$$

Equation S2. Equation used to determine equilibrium constants from competition experiments using $\mathbf{1}_{\mathbf{OMe,Me}}$ as the competitor. (Note – unbound isomer peaks H^*_A and H^*_C need to be accounted for in the calculation of unbound species)

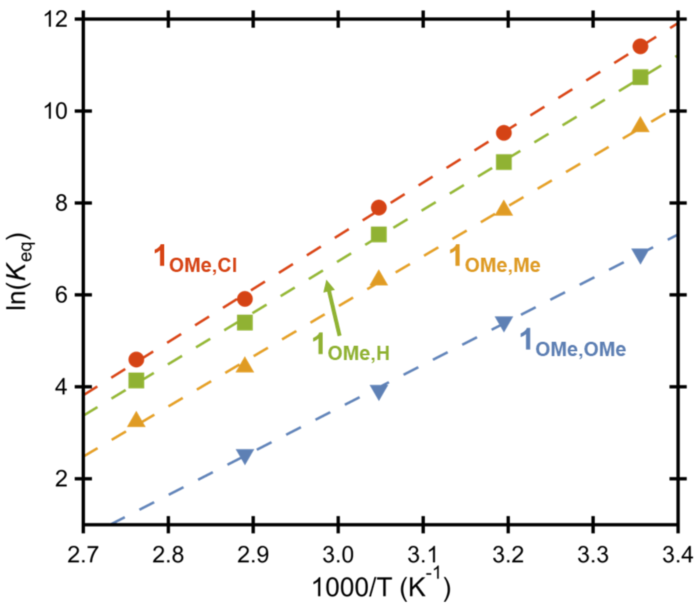

Figure S3. Van't Hoff plots of $\mathbf{1}_{\mathbf{OMe,Cl}}$ (red circles), $\mathbf{1}_{\mathbf{OMe,H}}$ (green squares), $\mathbf{1}_{\mathbf{OMe,Me}}$ (orange up arrows), $\mathbf{1}_{\mathbf{OMe,OMe}}$ (blue down arrows).

Table S1. Thermodynamic values extrapolated from linear fits displayed in Figure S3

Species	ΔH (kJ mol^{-1})	ΔS (J mol^{-1} K^{-1})
$\mathbf{1}_{\mathbf{OMe,Cl}}$	-96.2 ± 1.4	-228 ± 4
$\mathbf{1}_{\mathbf{OMe,H}}$	-93.1 ± 1.4	-223 ± 5
$\mathbf{1}_{\mathbf{OMe,Me}}$	-90.6 ± 1.7	-224 ± 5
$\mathbf{1}_{\mathbf{OMe,OMe}}$.	-78.5 ± 1.4	-206 ± 4

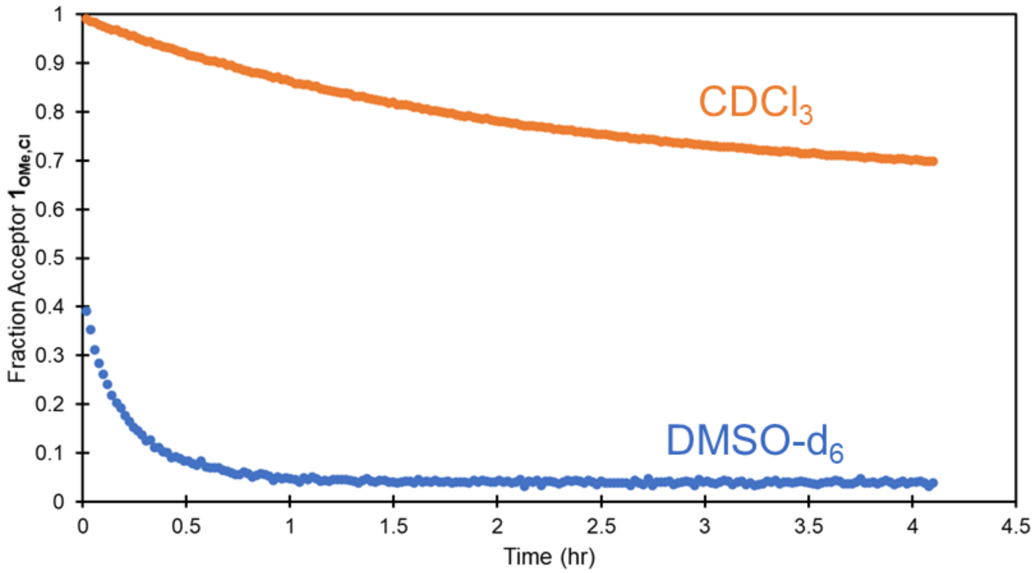

Figure S4. Fraction of free acceptor $\mathbf{1}_{\mathbf{OMe,Cl}}$ versus time during equilibration with octanethiol in DMSO-d_6 (blue circles) and $CDCl_3$ (orange circles).

2B. Thermal Properties

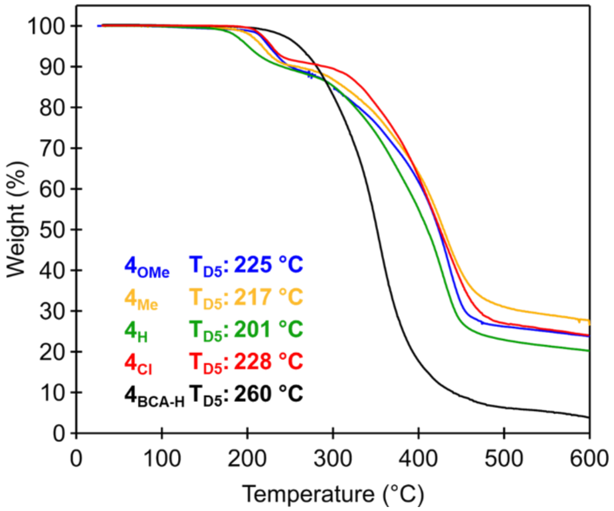

Figure S5. TGA data for **4**$_{OMe}$, **4**$_{Me}$, **4**$_{H}$, **4**$_{Cl}$, and **4**$_{BCA\text{-}H}$ (Heating rate = 10 °C/min).

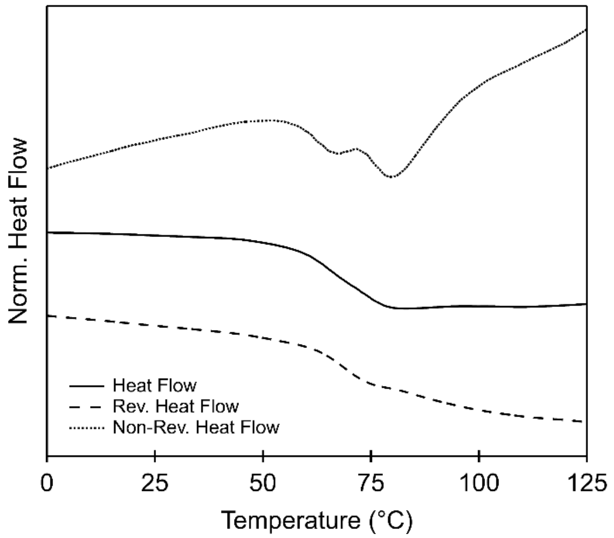

Figure S6. Modulated DSC data for **4**$_{H}$ shows two transitions in non-reversing heat flow, indicating convolution of T_g and T_{UT}.

2C. Atomic Force Microscopy

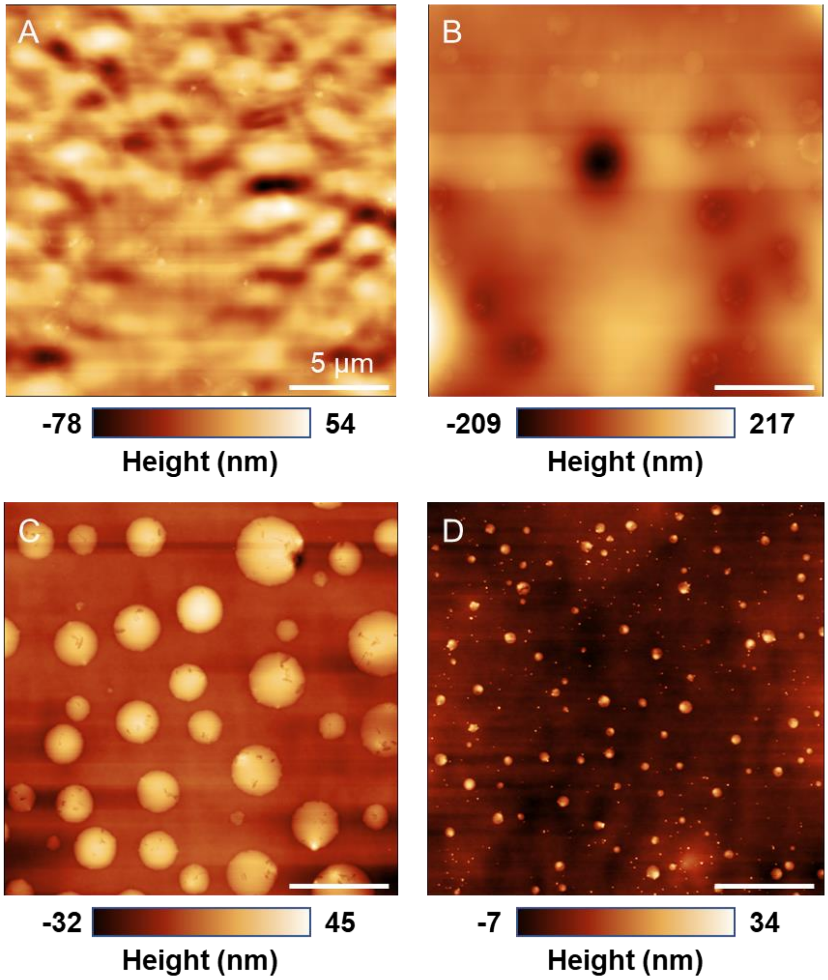

Figure S7. AFM height images corresponding to phase images shown in main text Figure 4c. (a) **4**$_{OMe}$, (b) **4**$_{Me}$, (c) **4**$_{H}$, and (d) **4**$_{Cl}$.

2D. Reprocessing Studies

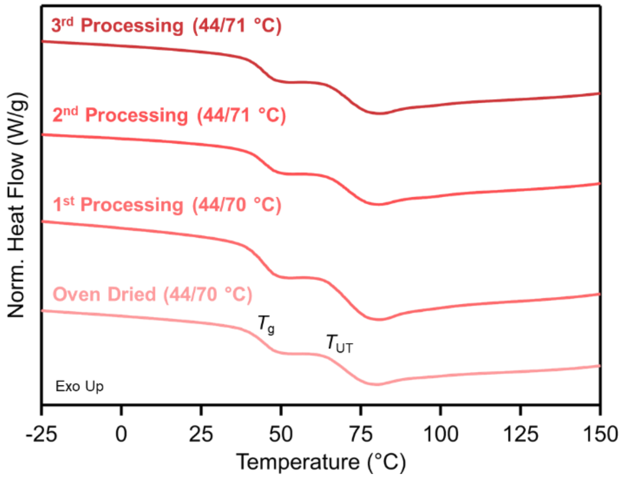

Figure S8. DSC data for $\mathbf{4}_{\mathbf{OMe}}$ after being oven dried and after three melt processing cycles showing T_g and T_{UT} remain constant across reprocessing cycles.

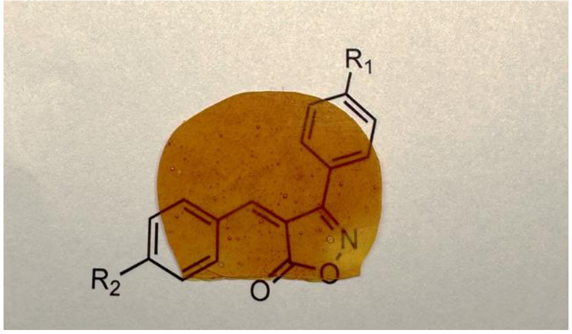

Figure S9. Photo of melt pressed dynamic network $\mathbf{4}_{\mathbf{OMe}}$ (third pressing cycle) over general BIOx schematic to demonstrate transparency of film.

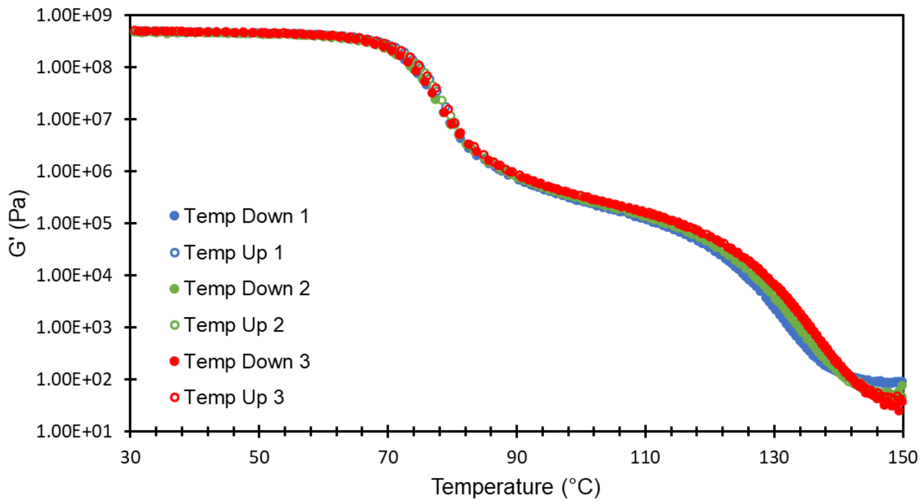

Figure S10. Shear rheology cooling and heating temperature ramps of **4**$_{\mathbf{H}}$ before and after applying high strains (4 full plate rotations) at 150 °C indicating stable thermomechanical properties after reprocessing.

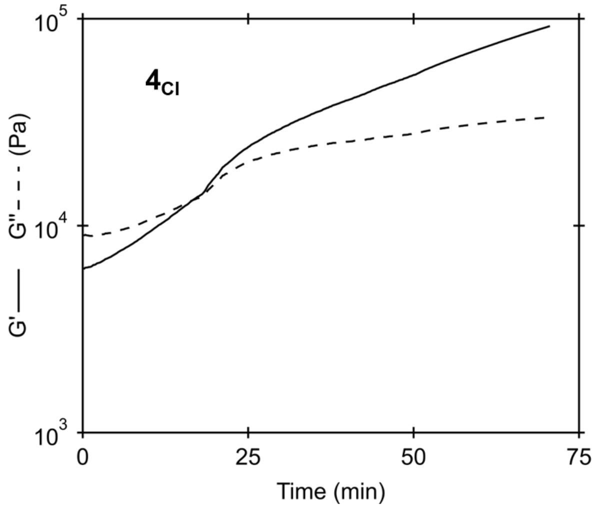

Figure S11. Storage and loss modulus versus time for **4**$_{\mathbf{Cl}}$ at 150 °C (frequency = 1 Hz, parallel plate geometries). The consistent growth seen indicates irreversible crosslinking reactions that can impact measured thermomechanical properties in as few as 10 minutes, as such, extended use of these materials at or above 150 °C should be limited.

2D. Stress Relaxation

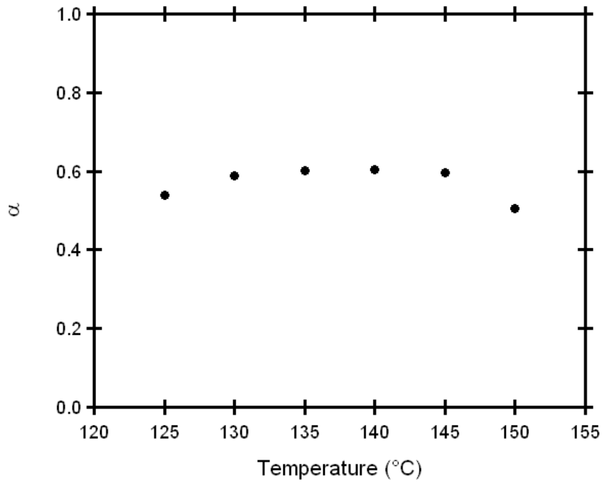

Figure S12. Stretching parameter from fits of stress relaxation data to main text Equation 1.

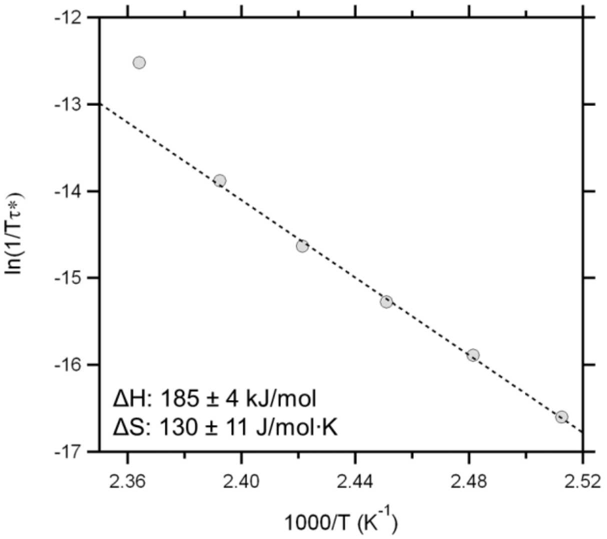

Figure S13. Temperature dependence of the rate of relaxation according to the adjusted Eyring equation. Note a deviation from linearity at elevated temperatures, in agreement with the Arrhenius analysis. Reported error corresponds to standard error of the linear fit

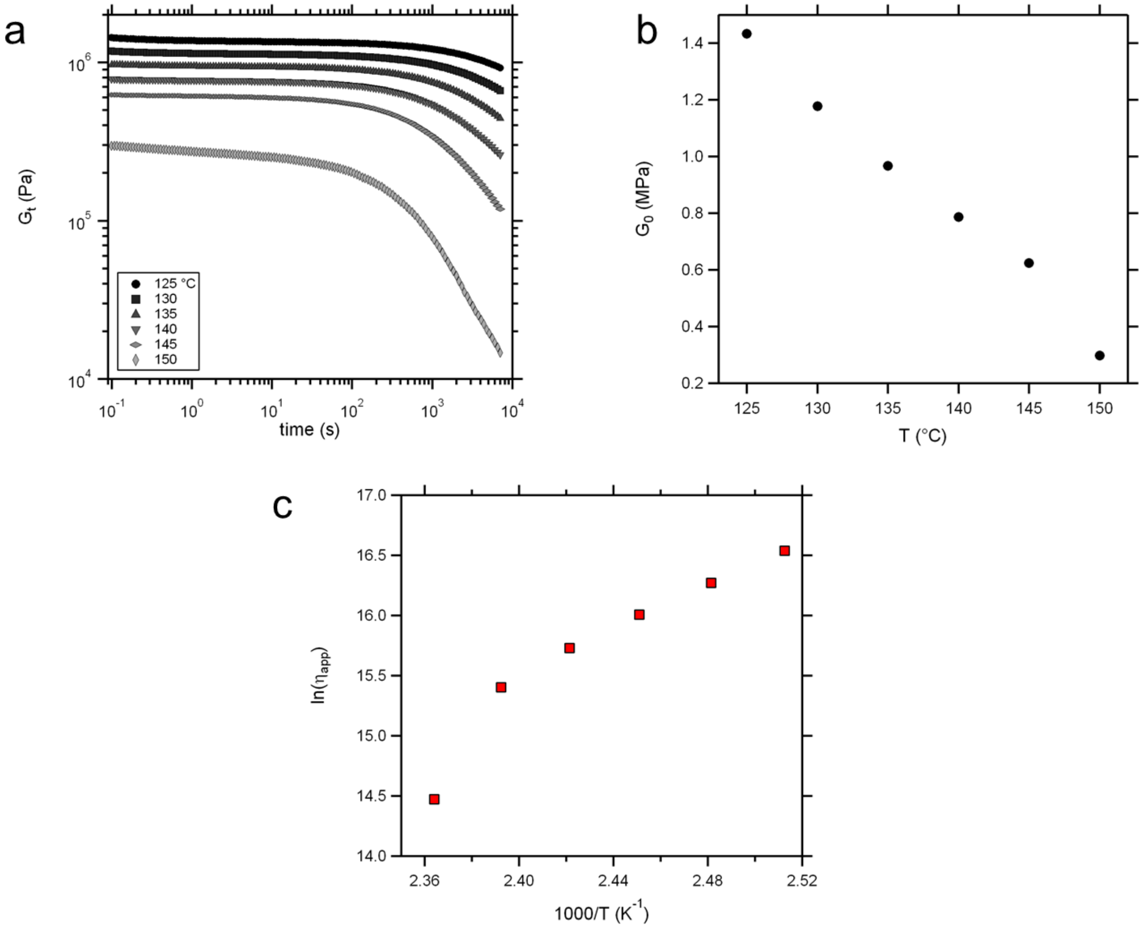

Figure S14. a) Non-normalized stress relaxation graphs corresponding to data shown in main text Figure 5b. b) Initial relaxation modulus plotted as a function of temperature. c) The apparent viscosity of the network calculated as $\eta_{app} = G_0 \cdot \tau^*$ as described in the literature.[11]

2F. Creep Measurements

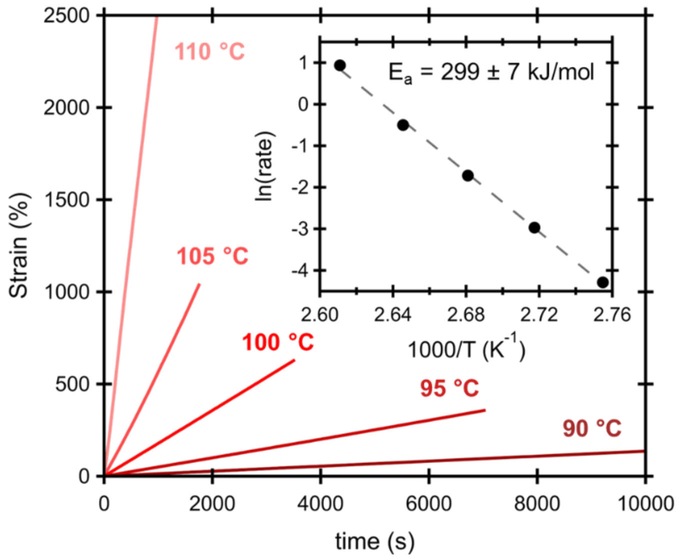

Figure S15. Creep test of $\mathbf{4_{Cl}}$ network at 90, 95, 100, 105, and 110 °C. Inset is of the Arrhenius plot of ln(rate) versus 1000/T and activation energy (299 ± 7 kJ/mol) extrapolated from the plot.

2G. Solution Viscometry

Critical polymerization concentrations (CPC) of the ditopic thia-Michael acceptors (**2_R**) were determined by determining the zero shear specific viscosities (η_{sp}) of linear polymer solutions of varying concentration. Figure S8 shows the viscosity versus shear rate plots for selected concentrations of **3_H**.

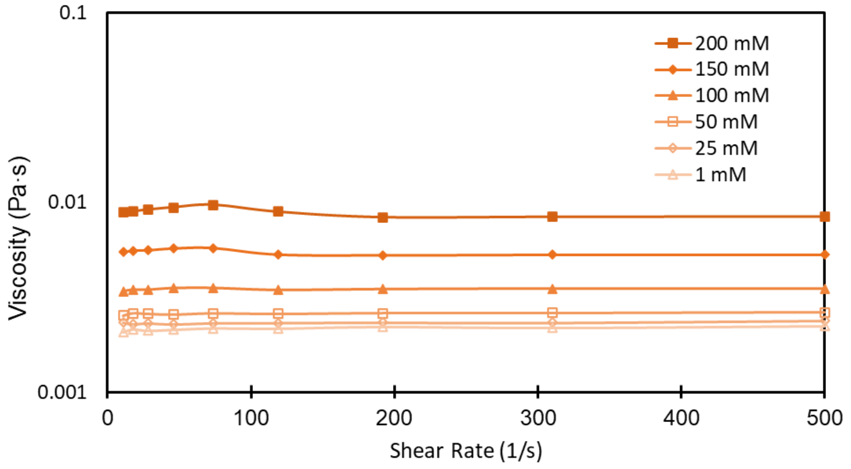

Figure S16. Representative plots of viscosity vs. shear rate for various concentrations of **3_H**.

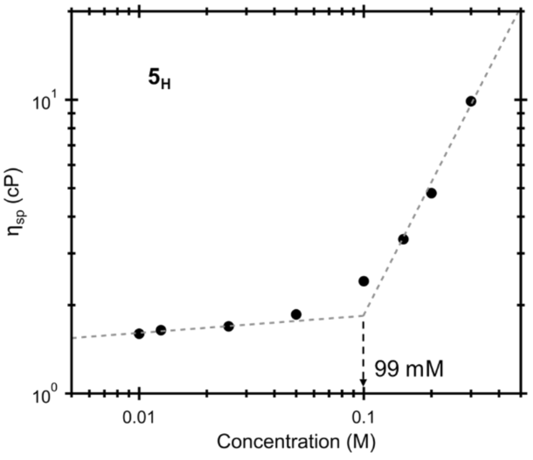

Figure S17. Log-log plot of zero-shear viscosity versus various concentrations of **3_H** at 50 °C.

REFERENCES

(1) Jurberg, I. D. An Aminocatalyzed Stereoselective Strategy for the Formal α-Propargylation of Ketones. *Chem. Eur. J.* **2017**, *23* (41), 9716–9720. https://doi.org/10.1002/chem.201701433.

(2) Hellmuth, T.; Frey, W.; Peters, R. Regioselective Catalytic Asymmetric C-Alkylation of Isoxazolinones by a Base-Free Palladacycle-Catalyzed Direct 1,4-Addition. *Angew. Chem. Int. Ed.* **2015**, *54*, 2788–2791. https://doi.org/10.1002/anie.201410933.

(3) Martinez-Pardo, P.; Lavios, A.; Sanz-Marco, A.; Vila, C.; Pedro, J. R.; Blay, G. Enantioselective Synthesis of Functionalized Diazaspirocycles from 4-Benzylidenisoxazol-5(4H)-One Derivatives and Isocyanoacetate Esters. *Adv. Synth. Catal.* **2020**, *362*, 3564. https://doi.org/10.1002/adsc.202000611.

(4) Clark, A. D.; Ha, U. T.; Prager, R. H.; Smith, J. A. High-Temperature Rearrangements of 2-Acylisoxazol-5(2H)-Ones and Related Oxazoles. *Aust. J. Chem.* **1999**, *52*, 1029–1033. https://doi.org/10.1071/CH99075.

(5) De Castro, P. P.; Dos Santos, J. A.; De Siqueira, M. M.; Batista, G. M. F.; Dos Santos, H. F.; Amarante, G. W. Quantum Chemical-Guided Steglich Rearrangement of Azlactones and Isoxazolones. *J. Org. Chem.* **2019**, *84* (19), 12573–12582. https://doi.org/10.1021/acs.joc.9b02099.

(6) Dias-Jurberg, I.; Gagosz, F.; Zard, S. Z. Unusual Approach to Branched 3-Alkynylamides and to 1,5-Dihydropyrrol-2- Ones. *Org. Lett.* **2010**, *12* (3), 416–419. https://doi.org/10.1021/ol902472r.

(7) Herbert, K. M.; Getty, P. T.; Dolinski, N. D.; Hertzog, J. E.; de Jong, D.; Lettow, J. H.; Romulus, J.; Onorato, J. W.; Foster, E. M.; Rowan, S. J. Dynamic Reaction-Induced Phase Separation in Tunable, Adaptive Covalent Networks. *Chem. Sci.* **2020**, *11* (19), 5028–5036. https://doi.org/10.1039/d0sc00605j.

(8) Zhang, X.; Wang, J.-H.; Tan, D.; Li, Q.; Li, M.; Gong, Z.; Tang, C.; Liu, Z.; Dong, M.; Lei, X. Carboxylate-Selective Chemical Cross-Linkers for Mass Spectrometric Analysis of Protein Structures. *Anal. Chem.* **2018**, *90*, 1195–1201. https://doi.org/10.1021/acs.analchem.7b03789.

(9) Lei, X.; Jones, A.; Dong, M.; Cao, Y.; Tan, H. Chemical Crosslinking Agent for Protein and Preparation Method and Application Thereof. CN 107628937 A, 2018.

(10) Jha, N.; Singh, R. P.; Saxena, P.; Kapur, M. Iridium(III)-Catalyzed C(3)-H Alkylation of Isoquinolines via Metal Carbene Migratory Insertion. *Org. Lett.* **2021**, *23* (22), 8694–8698. https://doi.org/10.1021/acs.orglett.1c03054.

(11) Van Lijsebetten, F.; De Bruycker, K.; Van Ruymbeke, E.; Winne, J. M.; Du Prez, F. E. Characterising Different Molecular Landscapes in Dynamic Covalent Networks. *Chem. Sci.* **2022**, *32* (9), 12865–12875. https://doi.org/10.1039/d2sc05528g.

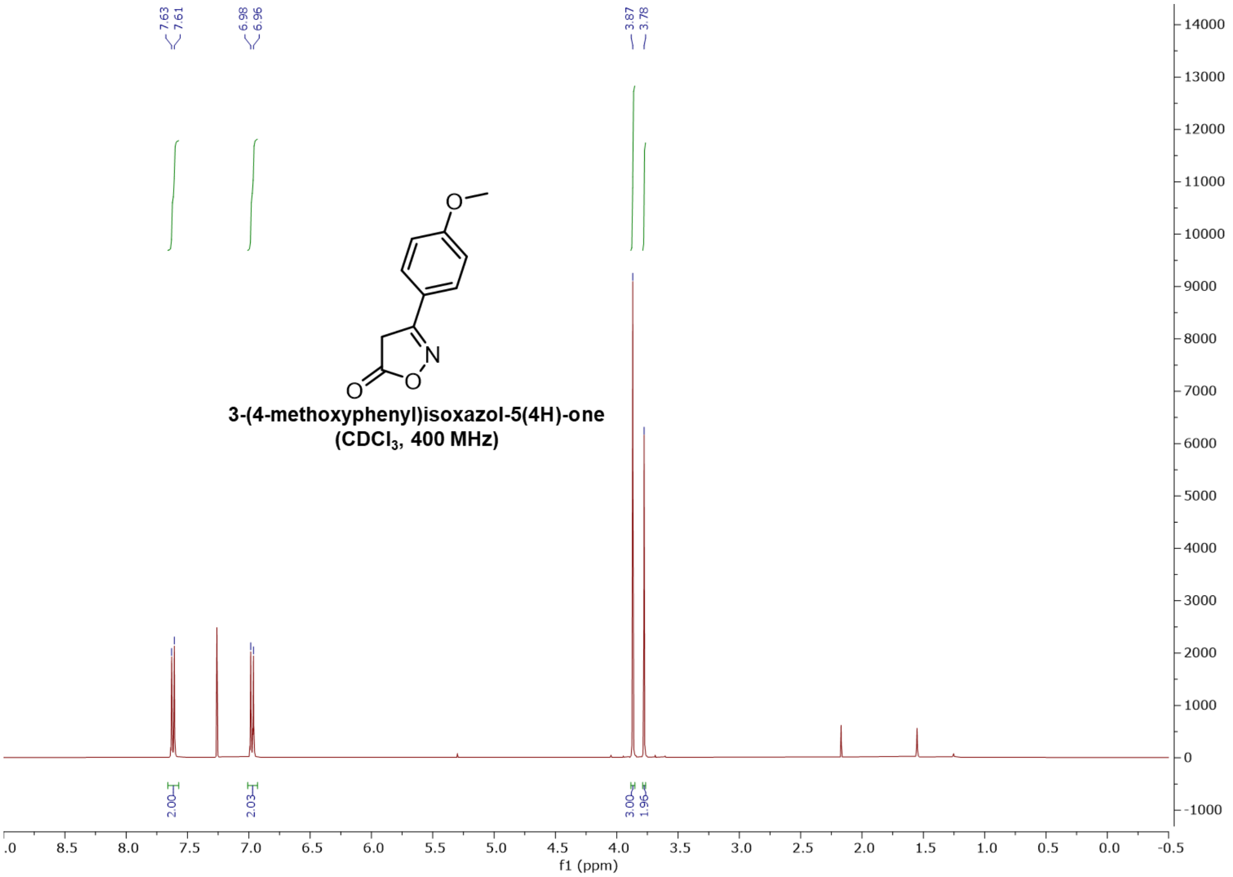

3-(4-methoxyphenyl)isoxazol-5(4H)-one
($CDCl_3$, 400 MHz)

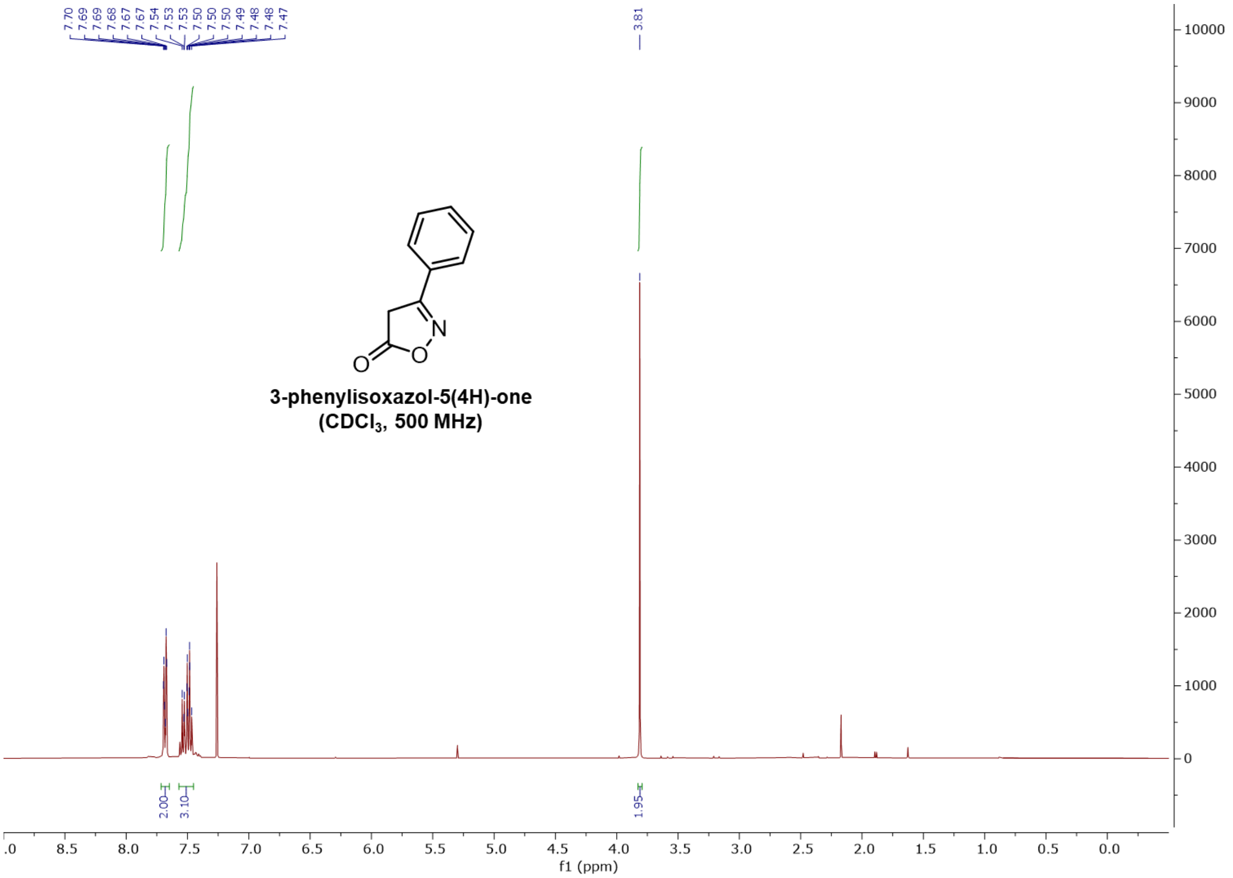
7.70
7.69
7.69
7.68
7.67
7.67
7.54
7.53
7.53
7.50
7.50
7.50
7.49
7.48
7.48
7.47
3.81
10000
9000
8000
7000
6000
5000
4000
3000
2000
1000
0
2.00
3.10
1.95
N
O
O
3-phenylisoxazol-5(4H)-one
(CDCl3, 500 MHz)
8.5
8.0
7.5
7.0
6.5
6.0
5.5
5.0
4.5
4.0
3.5
3.0
2.5
2.0
1.5
1.0
0.5
0.0
f1 (ppm)

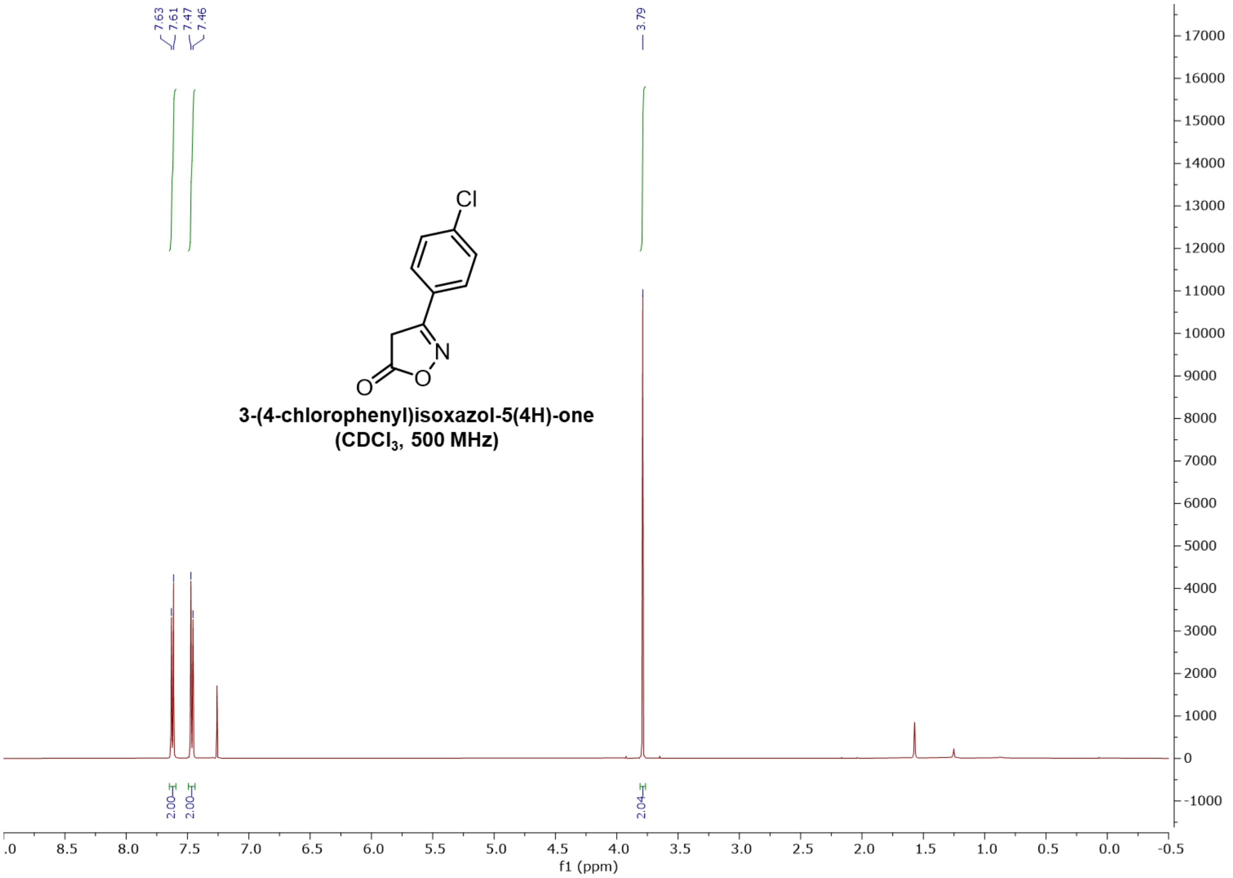

7.63
7.61
7.47
7.46
3.79
Cl
N
O
O
3-(4-chlorophenyl)isoxazol-5(4H)-one
(CDCl$_3$, 500 MHz)
2.00
2.00
2.04
f1 (ppm)

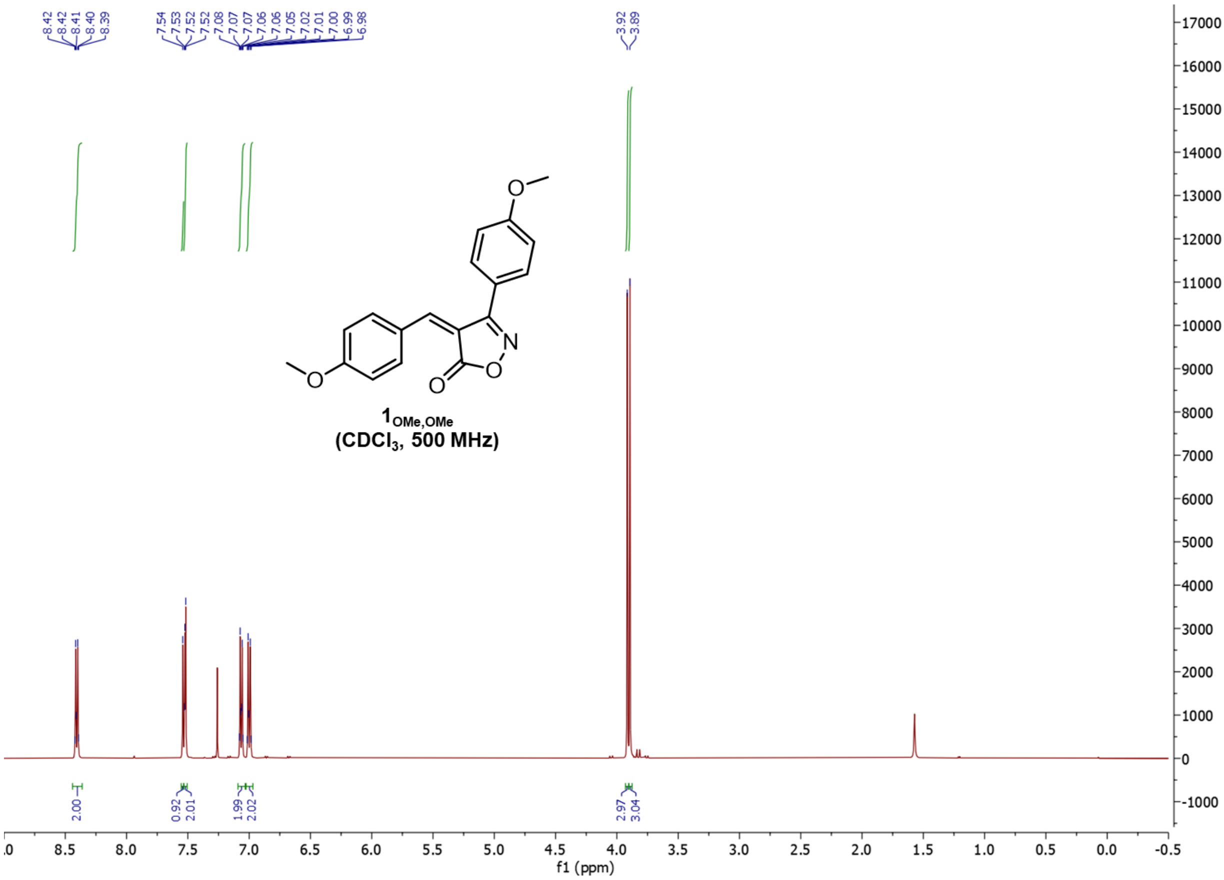
8.42
8.42
8.41
8.40
8.39
7.54
7.53
7.52
7.52
7.08
7.07
7.07
7.06
7.06
7.05
7.02
7.01
7.00
6.99
6.98
3.92
3.89
O
N
O
O
O
$1_{OMe,OMe}$
($CDCl_3$, 500 MHz)
2.00
0.92
2.01
1.99
2.02
2.97
3.04
f1 (ppm)

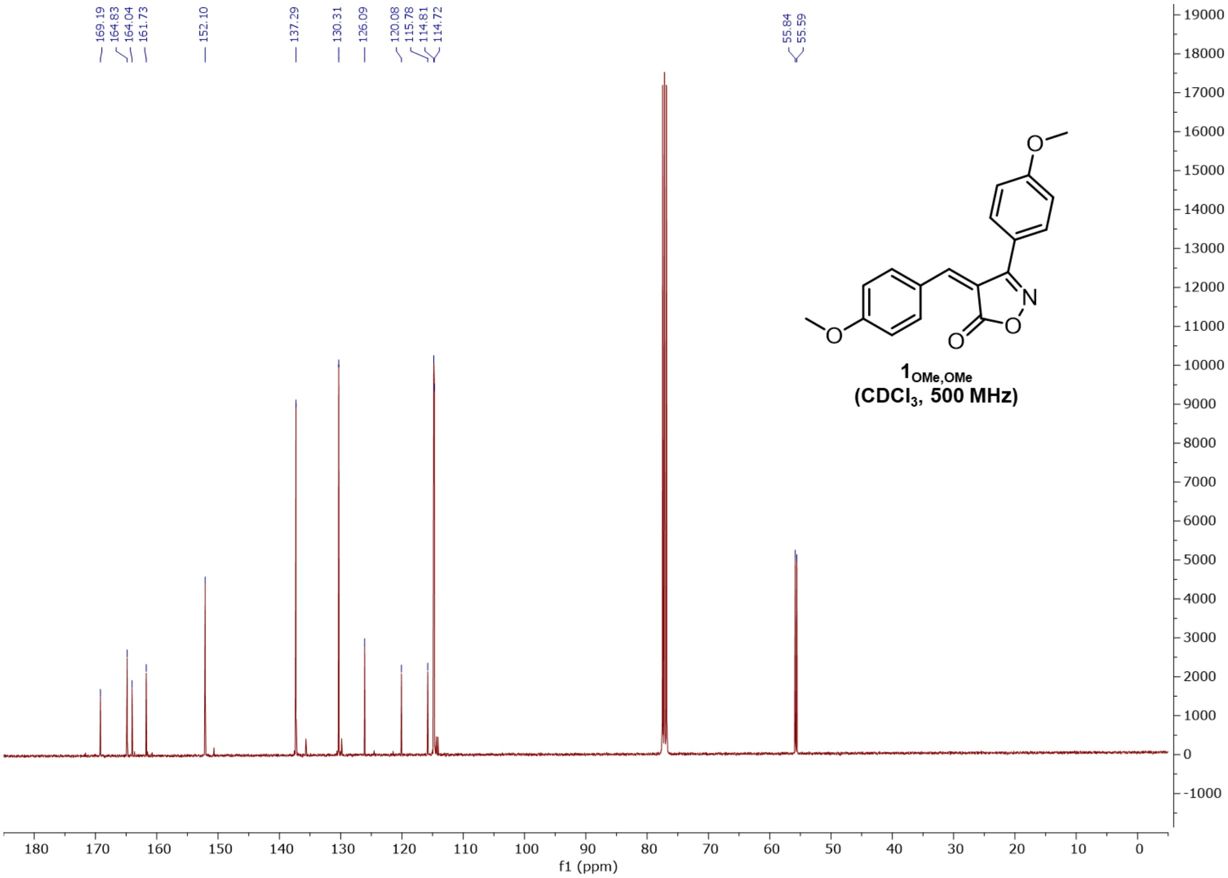
169.19
164.83
164.04
161.73
152.10
137.29
130.31
126.09
120.08
115.78
114.81
114.72
55.84
55.59
1OMe,OMe
(CDCl3, 500 MHz)
f1 (ppm)

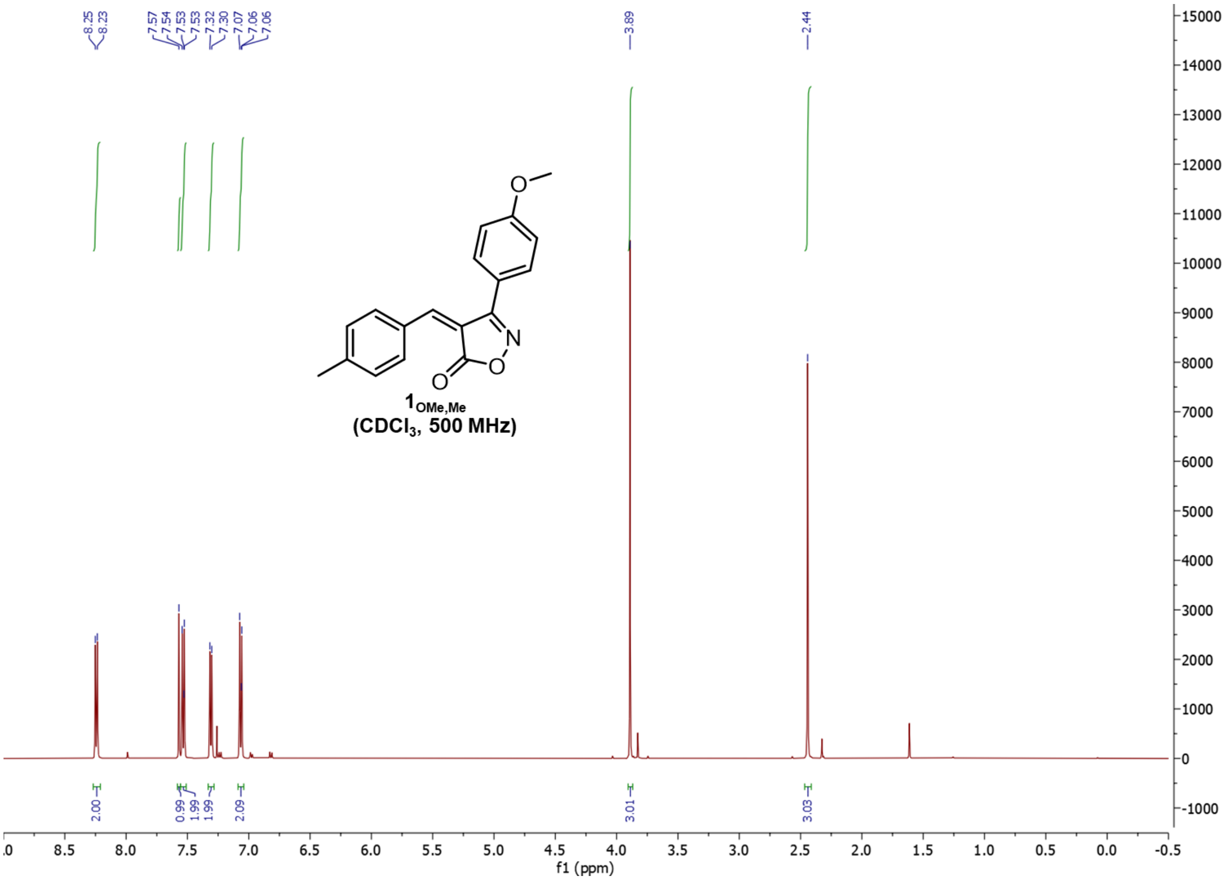
8.25
8.23
7.57
7.54
7.53
7.53
7.32
7.30
7.07
7.06
7.06
3.89
2.44
1OMe,Me
(CDCl3, 500 MHz)
2.00
0.99
1.99
1.99
2.09
3.01
3.03
f1 (ppm)

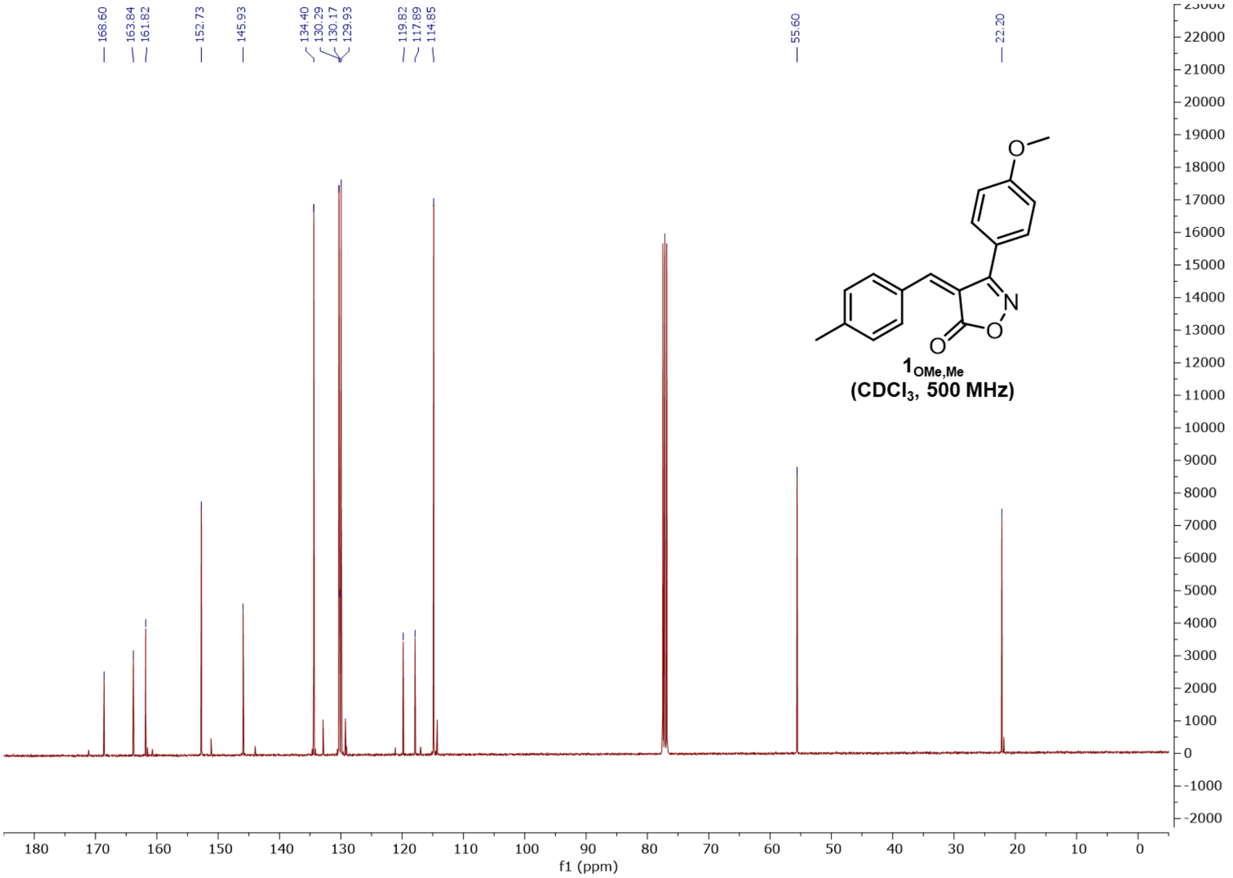
168.60
163.84
161.82
152.73
145.93
134.40
130.29
130.17
129.93
119.82
117.89
114.85
55.60
22.20
1OMe,Me
(CDCl3, 500 MHz)
f1 (ppm)

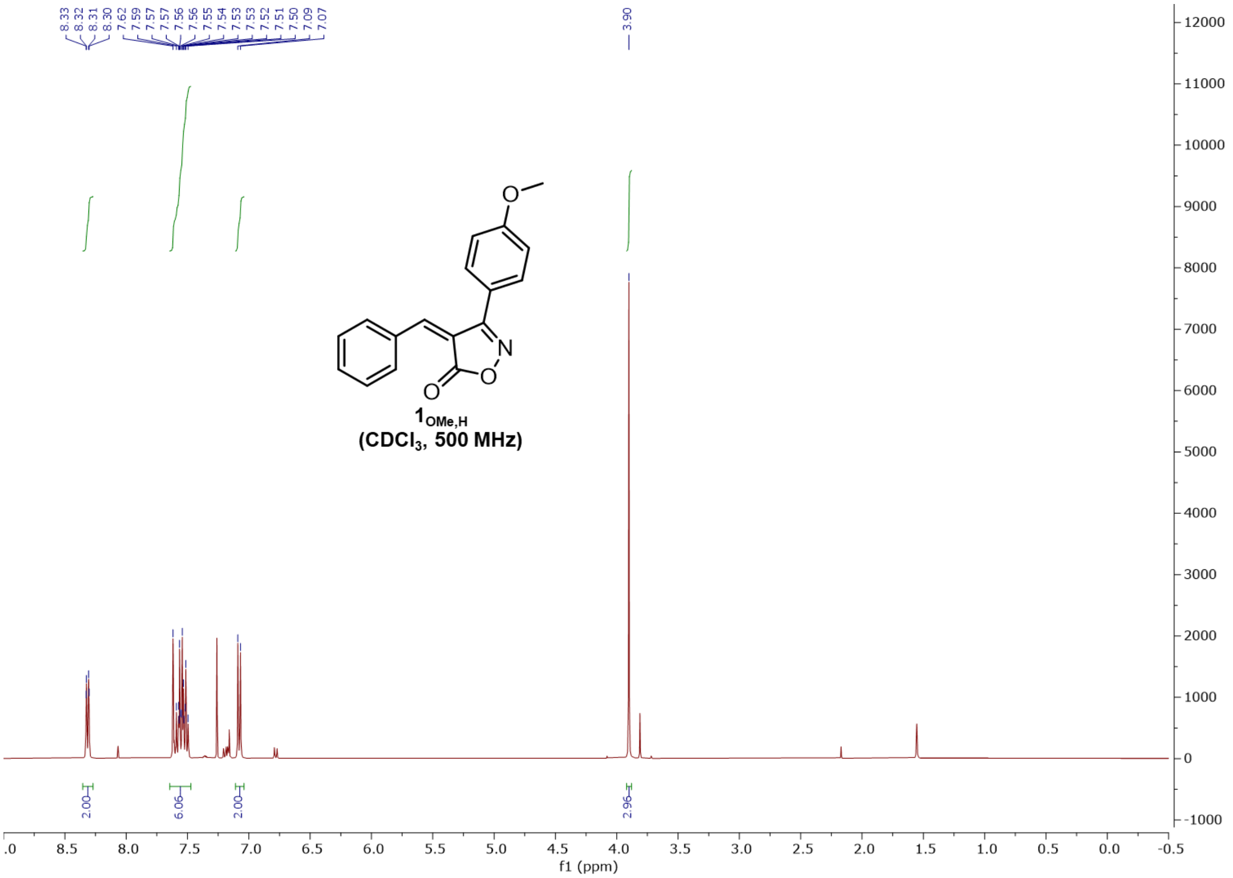
8.33
8.32
8.31
8.30
7.62
7.59
7.57
7.57
7.56
7.56
7.55
7.54
7.53
7.53
7.52
7.51
7.50
7.09
7.07
3.90
1OMe,H
(CDCl3, 500 MHz)
2.00
6.06
2.00
2.96
f1 (ppm)

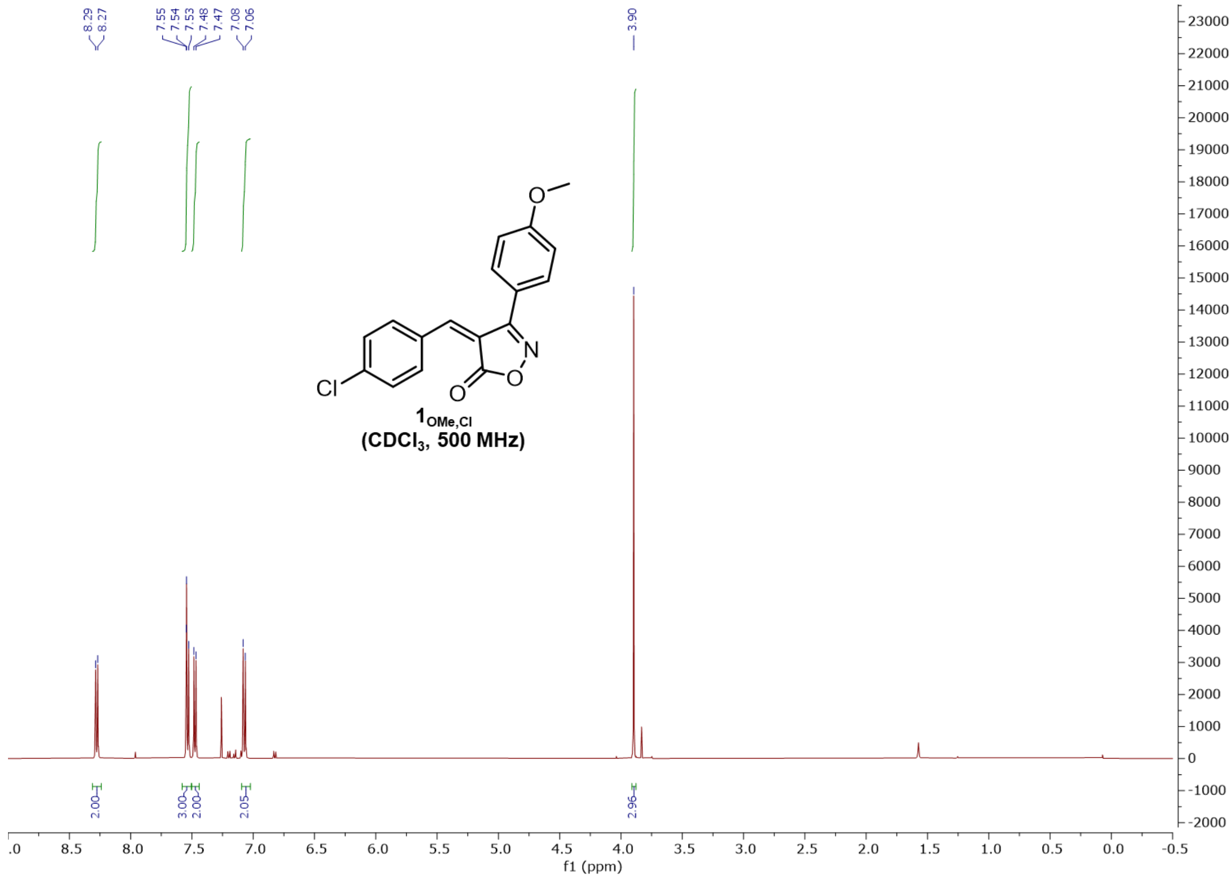
8.29
8.27
7.55
7.54
7.53
7.48
7.47
7.08
7.06
3.90
Cl
O
N
O
O
$1_{OMe,Cl}$
(CDCl$_3$, 500 MHz)
2.00
3.00
2.00
2.05
2.96
f1 (ppm)

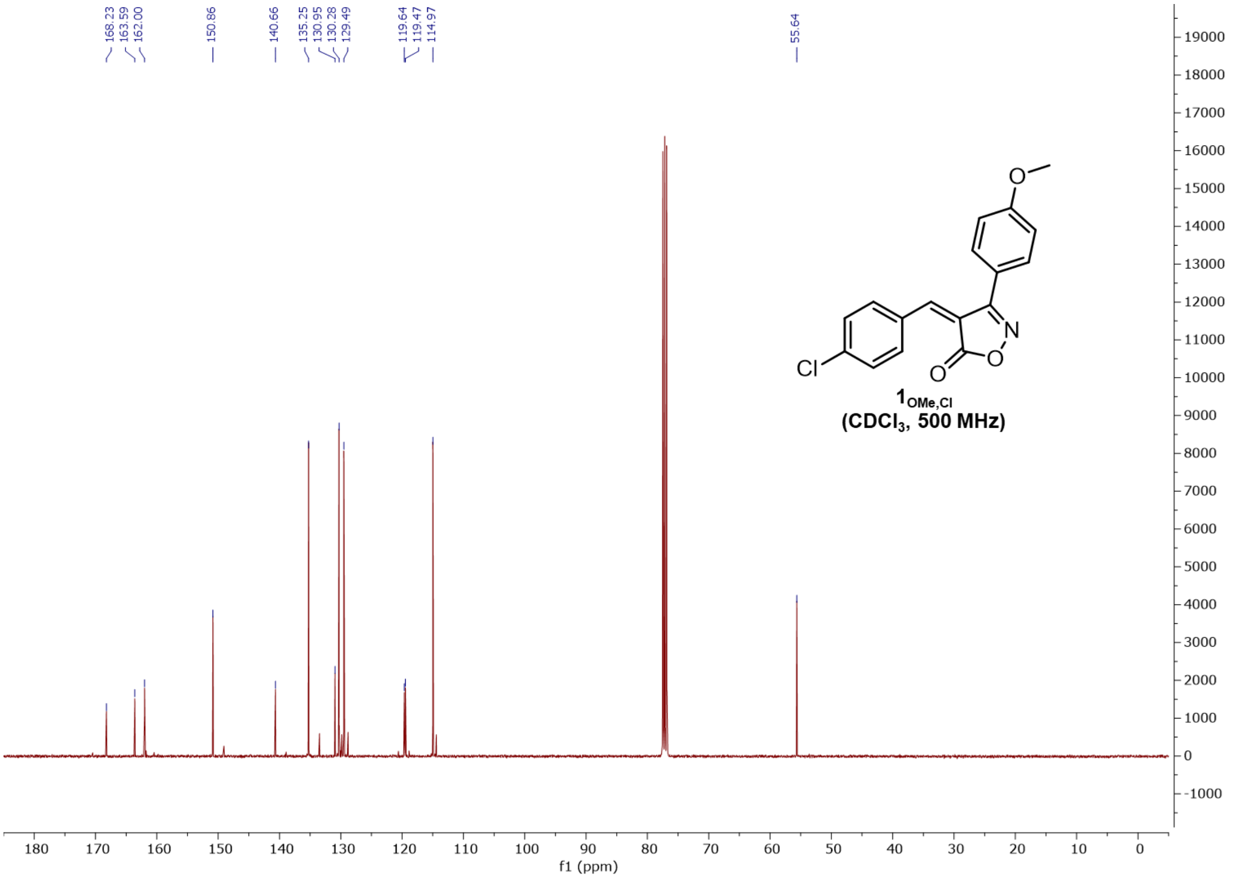
168.23
163.59
162.00
150.86
140.66
135.25
130.95
130.28
129.49
119.64
119.47
114.97
55.64
Cl
O
N
O
O
$1_{OMe,Cl}$
($CDCl_3$, 500 MHz)
f1 (ppm)

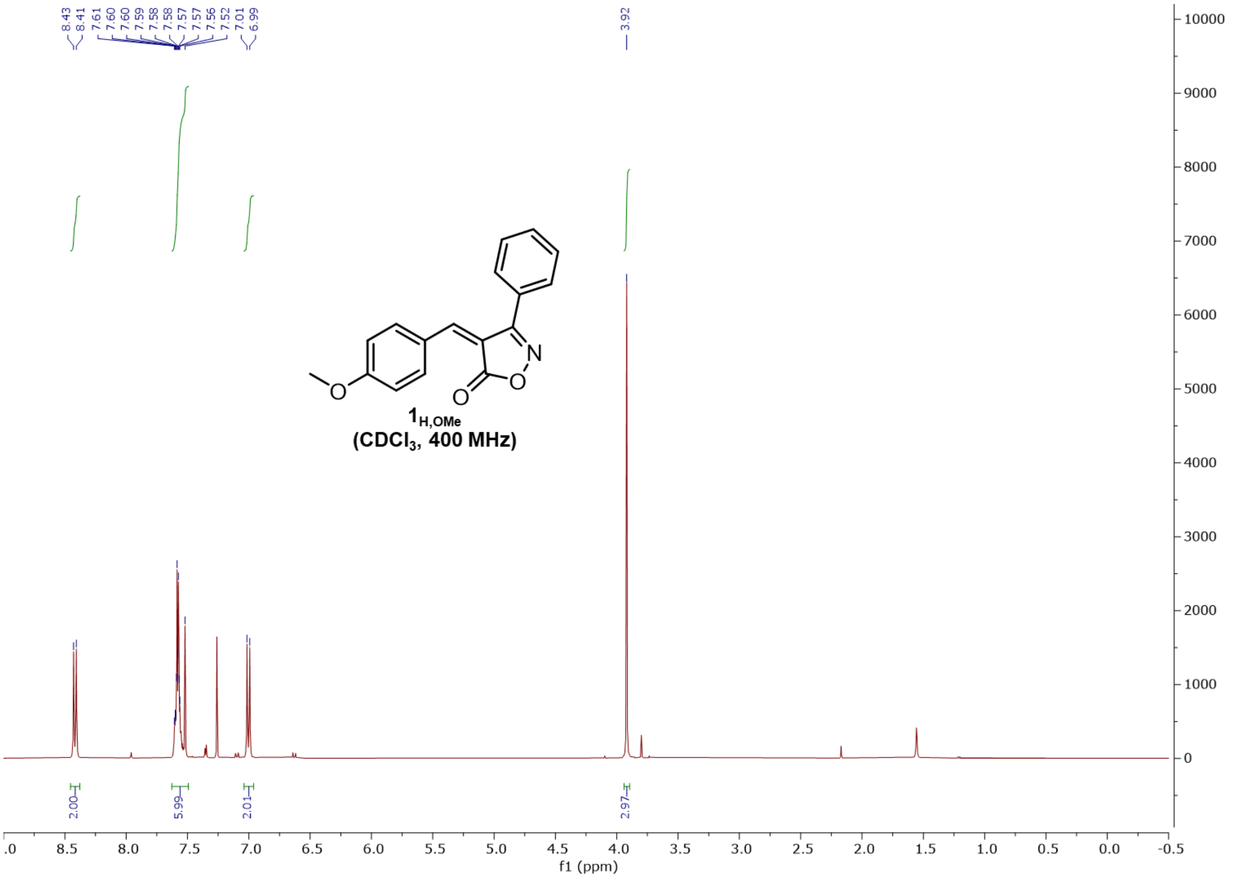
8.43
8.41
7.61
7.60
7.60
7.59
7.58
7.58
7.58
7.57
7.57
7.56
7.52
7.01
6.99
3.92
1H,OMe
(CDCl3, 400 MHz)
2.00
5.99
2.01
2.97
f1 (ppm)

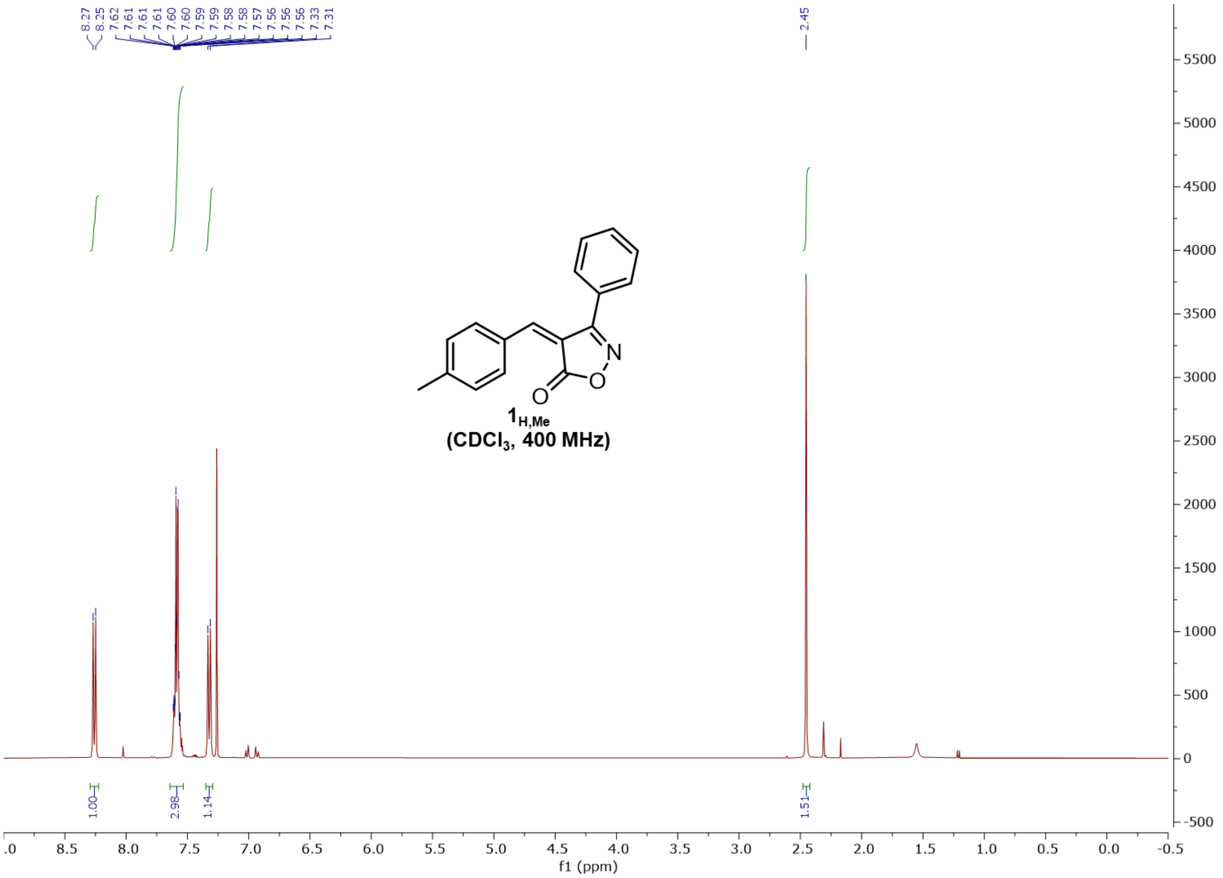

8.27
8.25
7.62
7.61
7.61
7.61
7.60
7.60
7.59
7.59
7.58
7.58
7.57
7.56
7.56
7.56
7.33
7.31
2.45
O
N
O
1H,Me
(CDCl3, 400 MHz)
1.00
2.98
1.14
1.51
f1 (ppm)

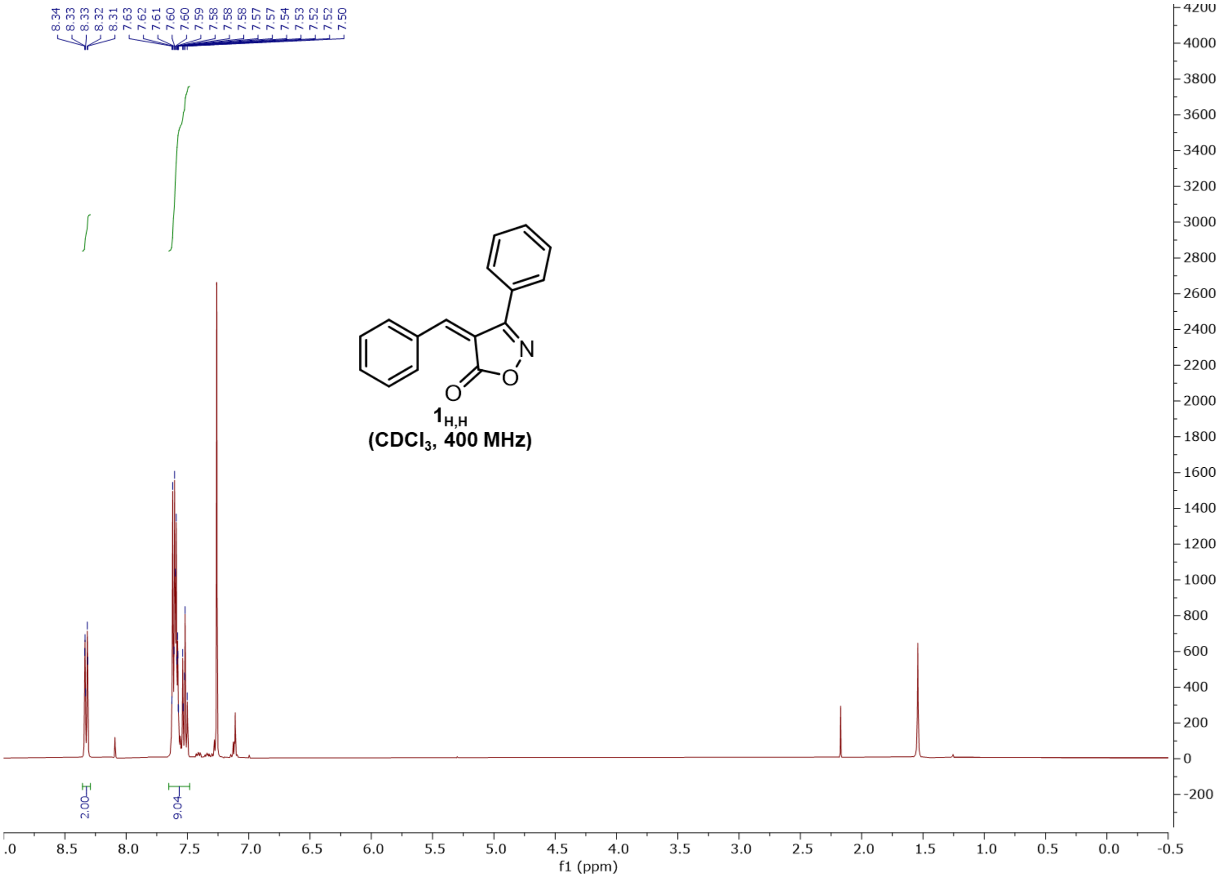
8.34
8.33
8.33
8.32
8.31
7.63
7.62
7.61
7.60
7.60
7.59
7.58
7.58
7.58
7.57
7.57
7.54
7.53
7.52
7.52
7.50
N
O
O
$1_{H,H}$
($CDCl_3$, 400 MHz)
2.00
9.04
f1 (ppm)

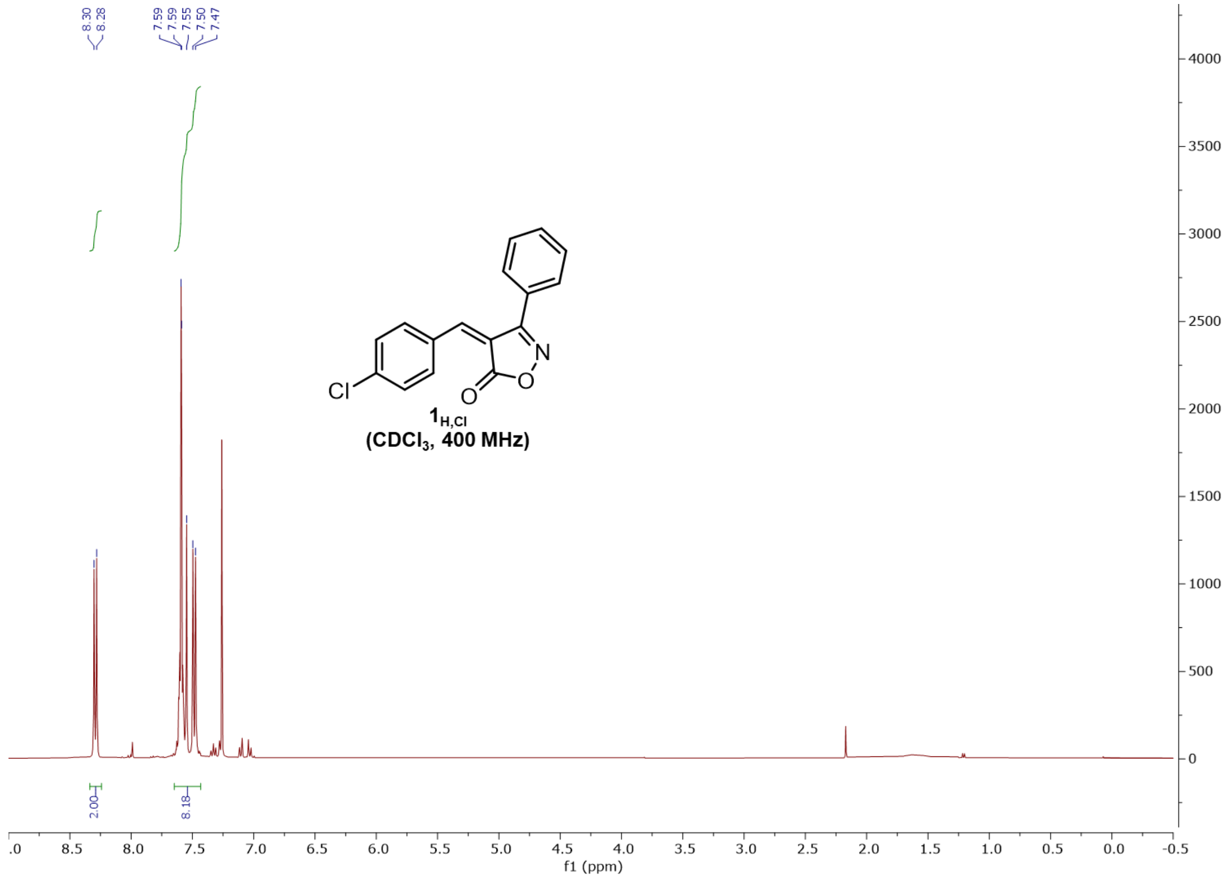
8.30
8.28
7.59
7.59
7.55
7.50
7.47
Cl
N
O
O
1H,Cl
(CDCl3, 400 MHz)
2.00
8.18
4000
3500
3000
2500
2000
1500
1000
500
0
f1 (ppm)

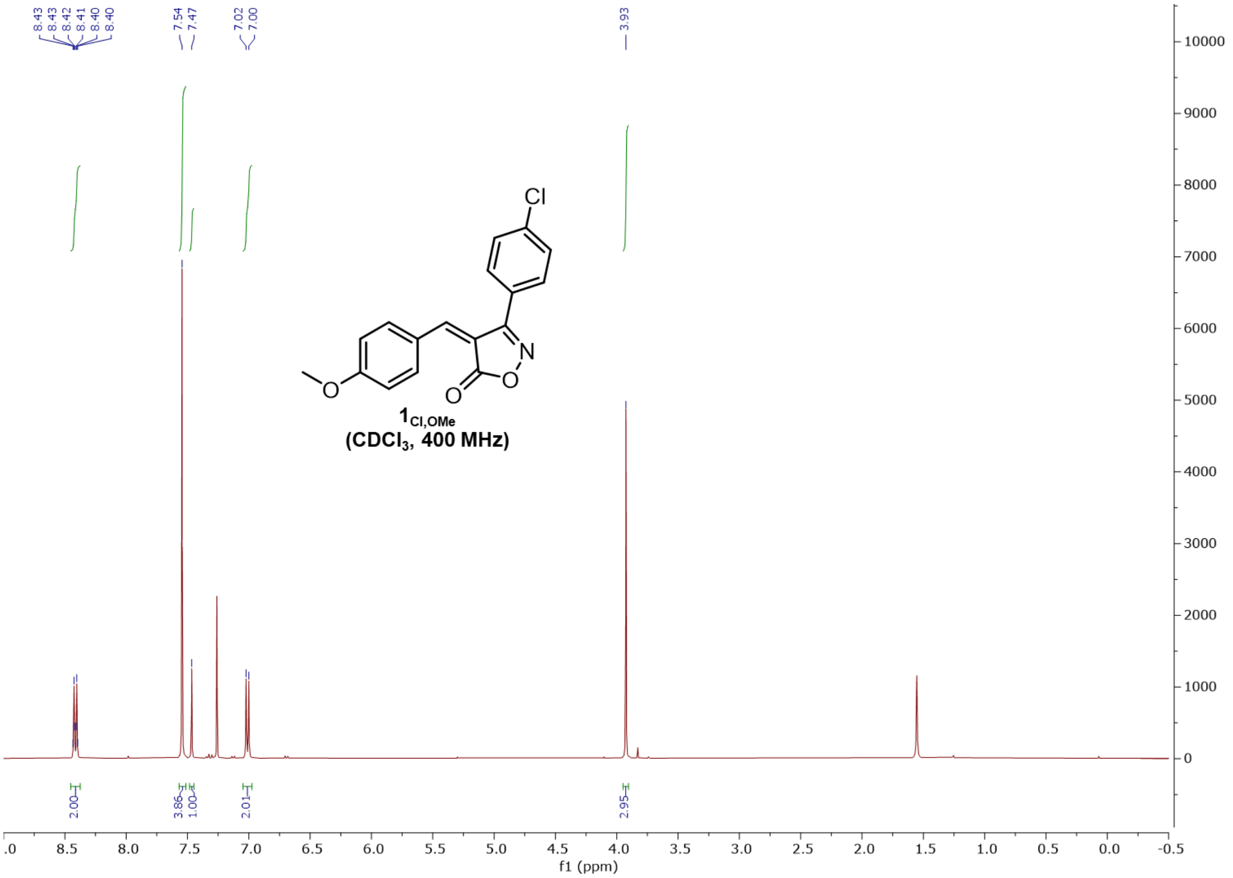
8.43
8.43
8.42
8.41
8.40
8.40
7.54
7.47
7.02
7.00
3.93
Cl
N
O
O
O
$1_{Cl,OMe}$
($CDCl_3$, 400 MHz)
2.00
3.86
1.00
2.01
2.95
f1 (ppm)

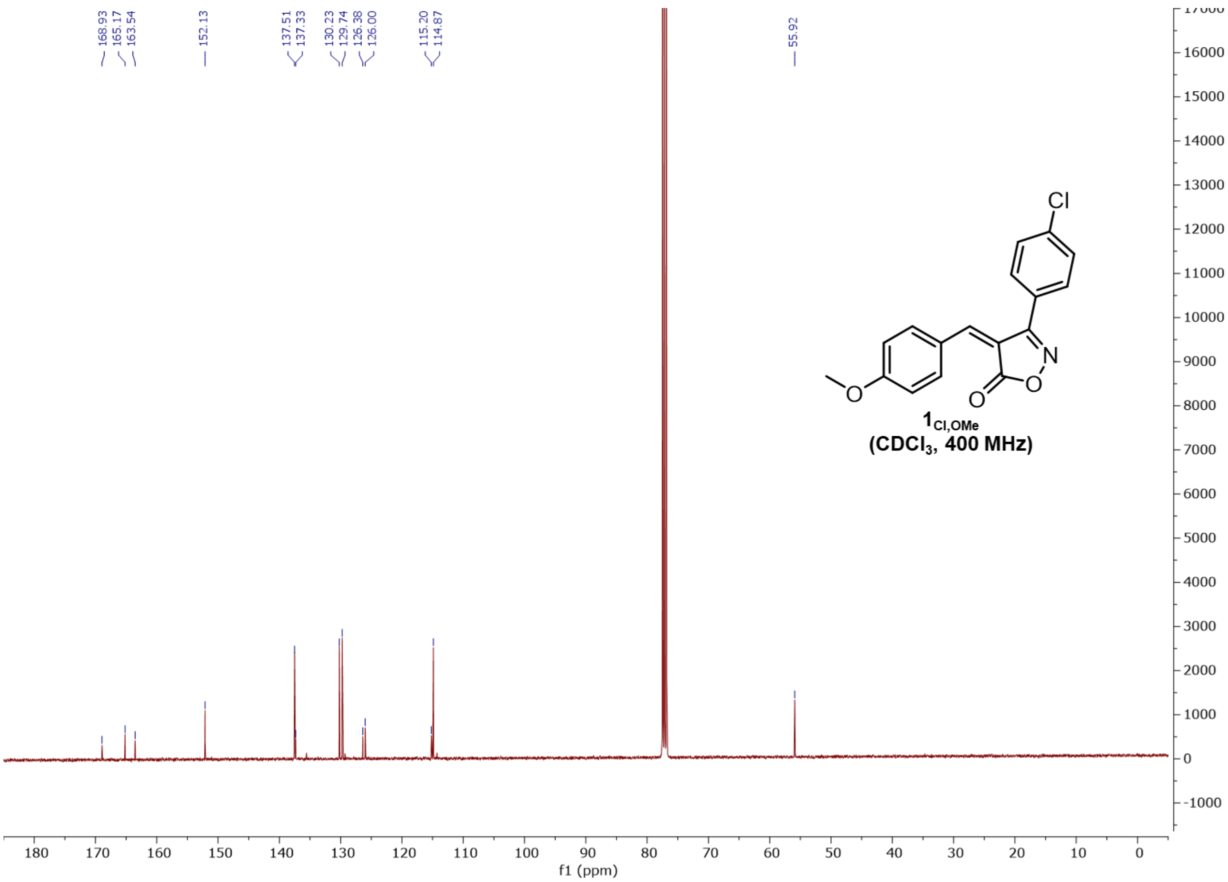
168.93
165.17
163.54
152.13
137.51
137.33
130.23
129.74
126.38
126.00
115.20
114.87
55.92
Cl
N
O
O
O
$1_{Cl,OMe}$
(CDCl$_3$, 400 MHz)
f1 (ppm)

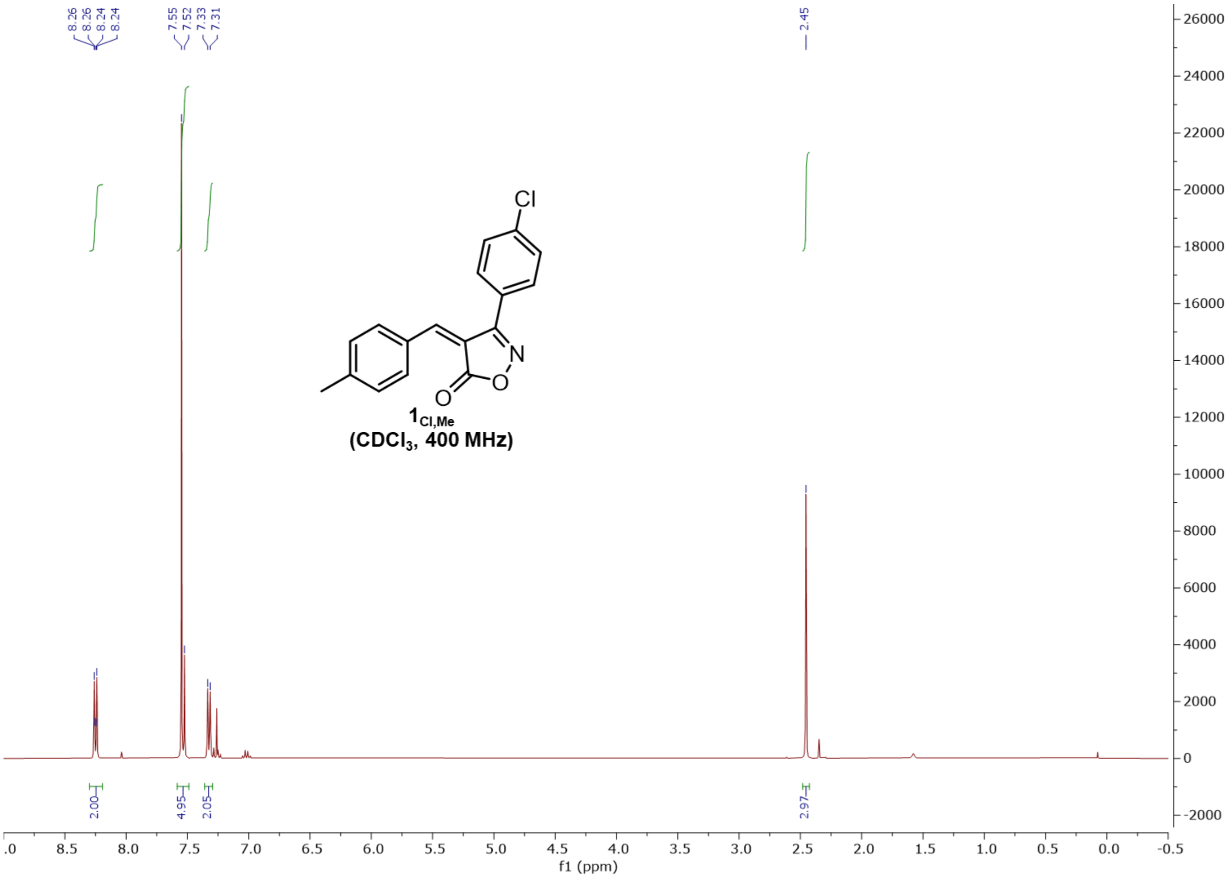
8.26
8.26
8.24
8.24
7.55
7.52
7.33
7.31
2.45
Cl
N
O
O
$1_{Cl,Me}$
($CDCl_3$, 400 MHz)
2.00
4.95
2.05
2.97
f1 (ppm)

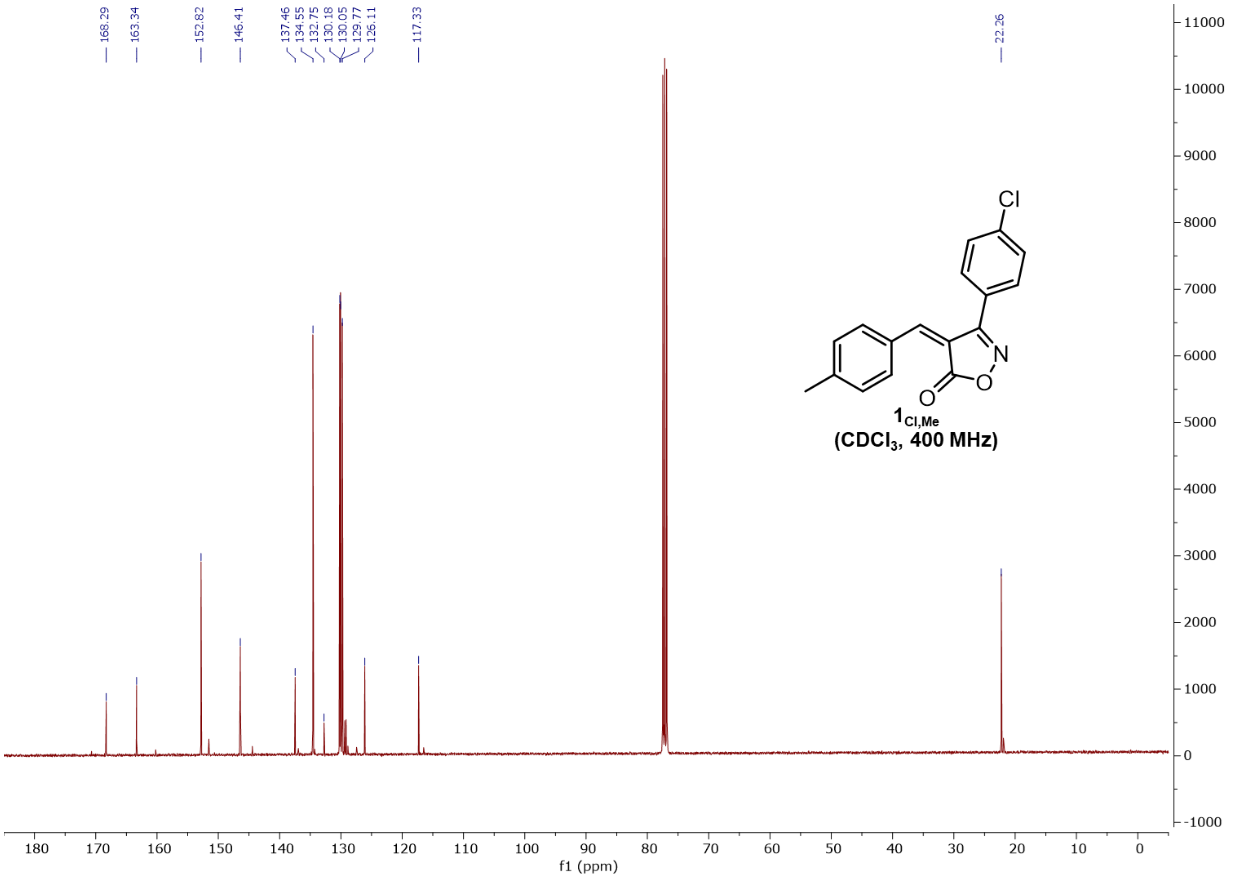

168.29
163.34
152.82
146.41
137.46
134.55
132.75
130.18
130.05
129.77
126.11
117.33
22.26
Cl
N
O
O
1Cl,Me
(CDCl3, 400 MHz)
f1 (ppm)

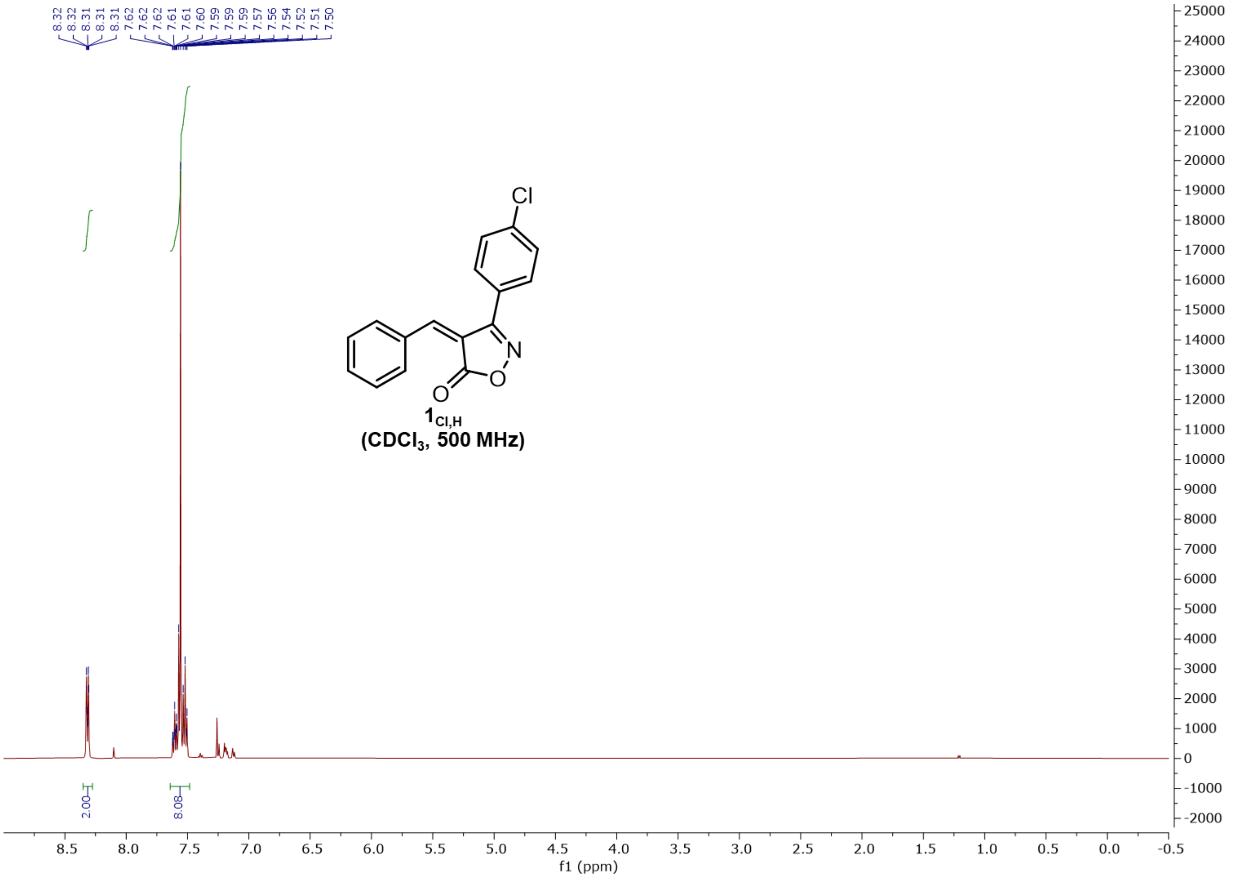

8.32
8.32
8.31
8.31
8.31
7.62
7.62
7.62
7.61
7.61
7.60
7.59
7.59
7.59
7.57
7.56
7.54
7.52
7.51
7.50
Cl
N
O
O
1Cl,H
(CDCl3, 500 MHz)
2.00
8.08
f1 (ppm)

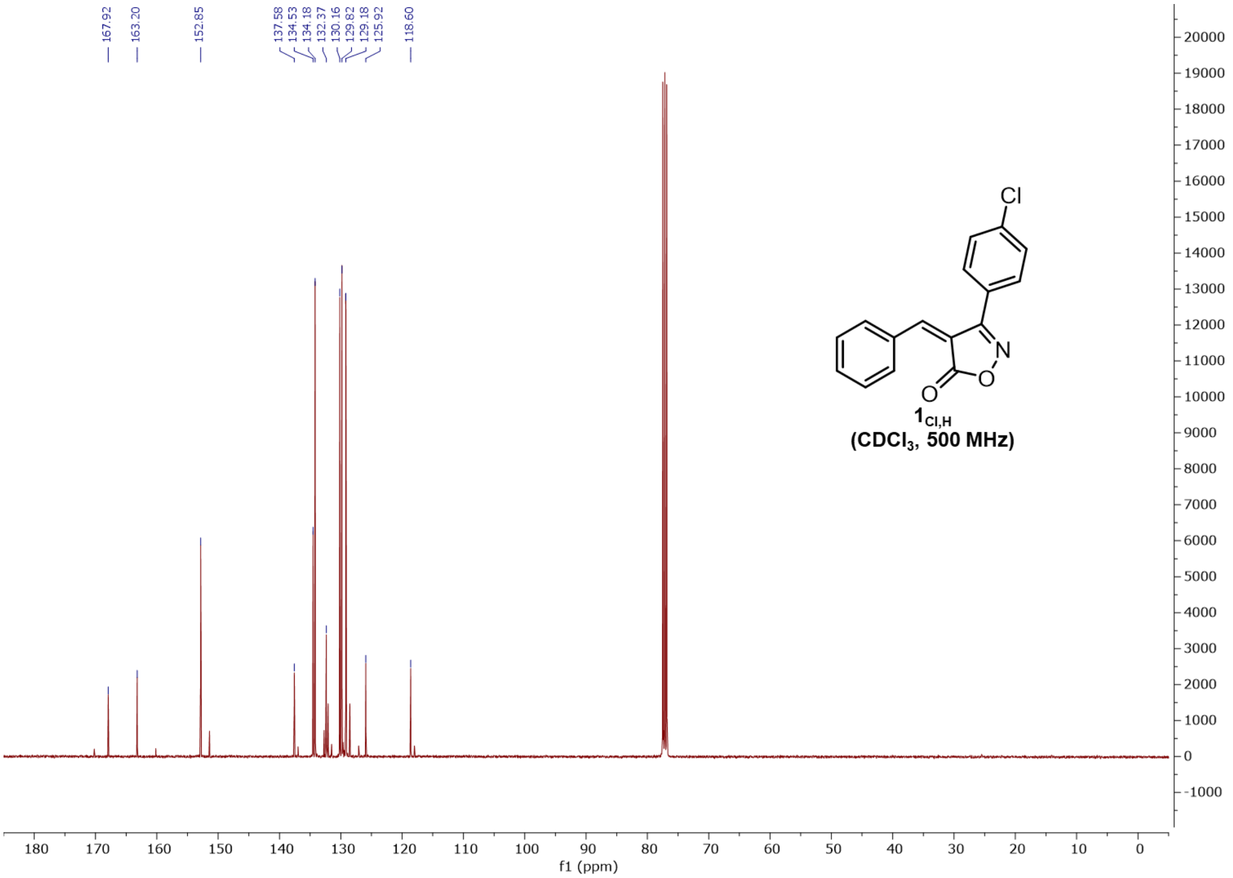

167.92
163.20
152.85
137.58
134.53
134.18
132.37
130.16
129.82
129.18
125.92
118.60
Cl
N
O
O
1Cl,H
(CDCl3, 500 MHz)
f1 (ppm)

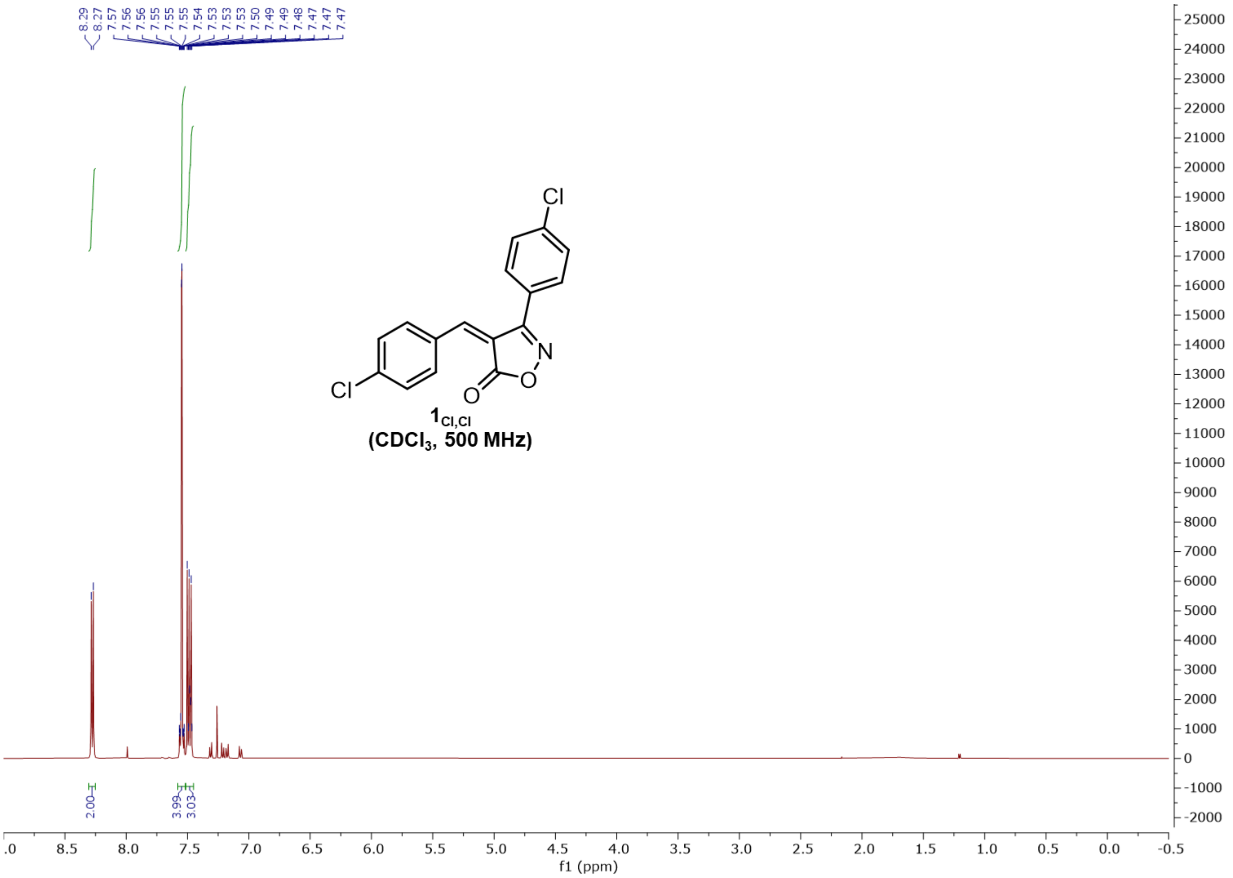
8.29
8.27
7.57
7.56
7.56
7.55
7.55
7.54
7.53
7.53
7.53
7.50
7.49
7.48
7.47
7.47
7.47
Cl
N
O
O
Cl
$1_{Cl,Cl}$
($CDCl_3$, 500 MHz)
2.00
3.99
3.03
f1 (ppm)

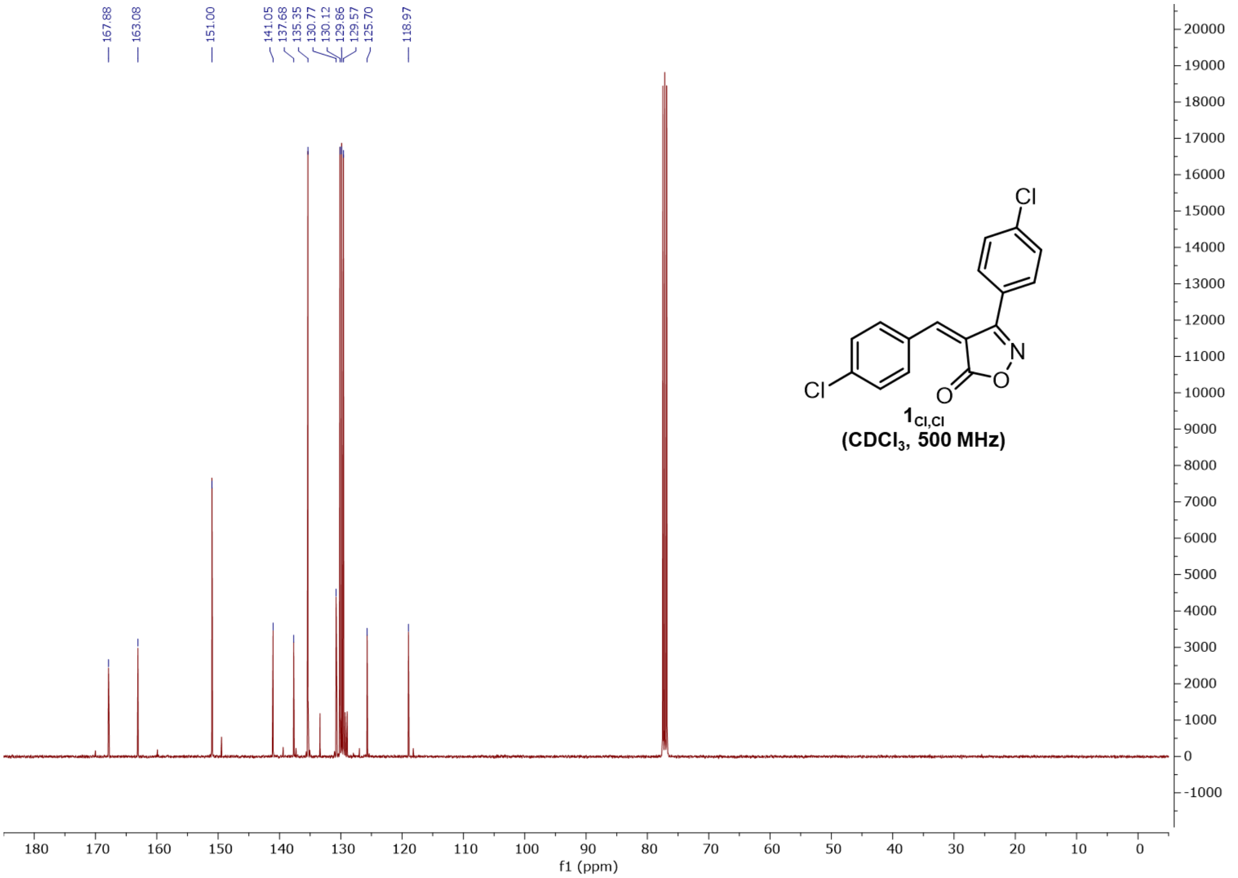

167.88
163.08
151.00
141.05
137.68
135.35
130.77
130.12
129.86
129.57
125.70
118.97
Cl
Cl
N
O
O
$1_{Cl,Cl}$
($CDCl_3$, 500 MHz)
f1 (ppm)

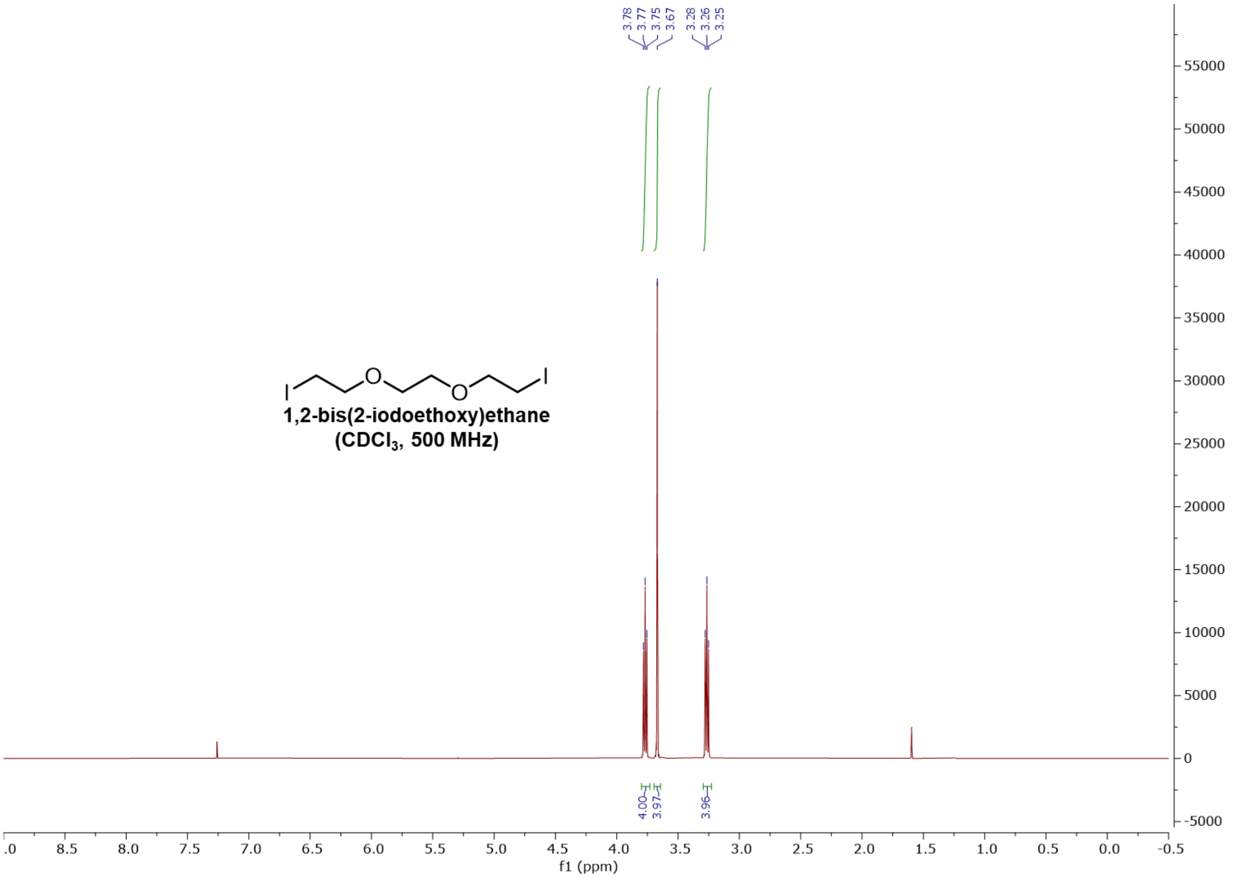
3.78
3.77
3.75
3.67
3.28
3.26
3.25
1,2-bis(2-iodoethoxy)ethane
(CDCl3, 500 MHz)
4.00
3.97
3.96
f1 (ppm)

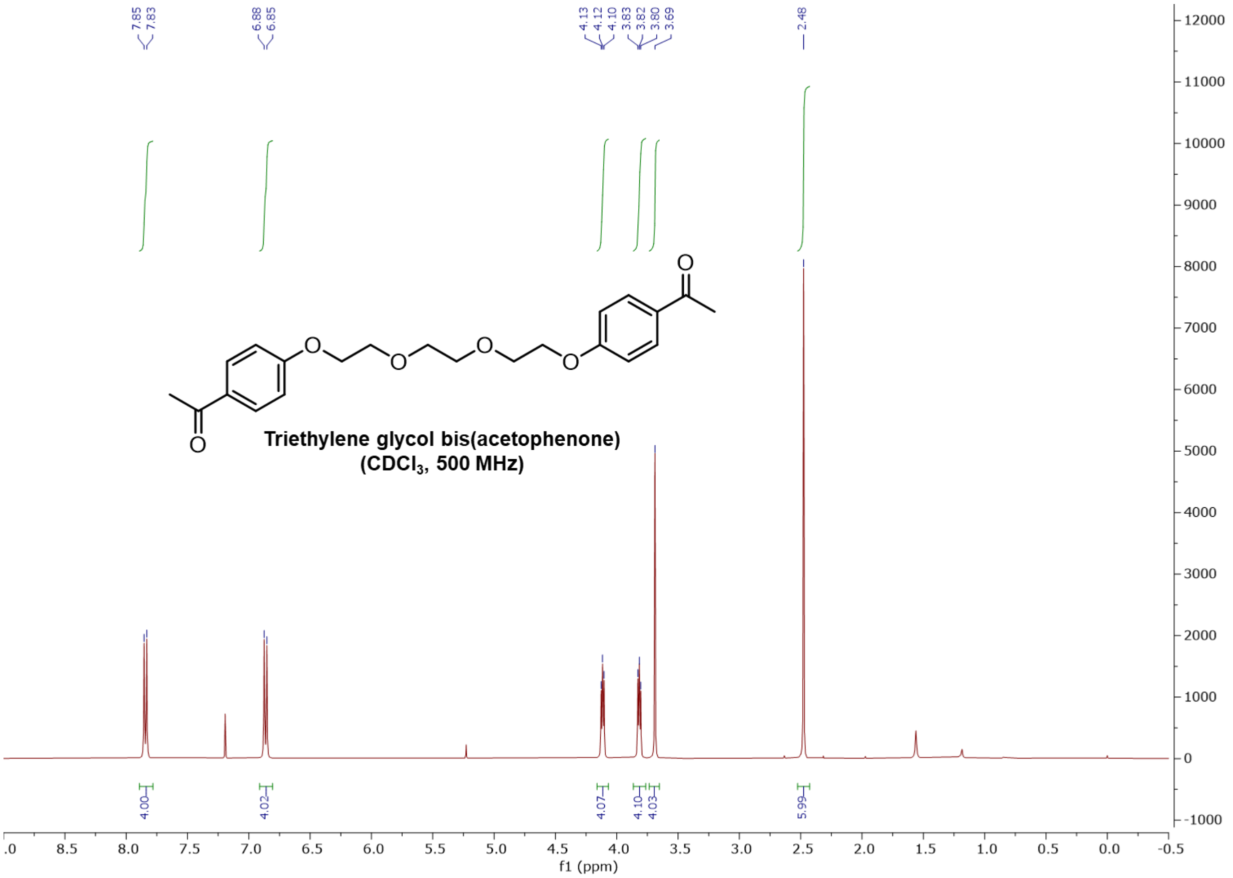

7.85
7.83
6.88
6.85
4.13
4.12
4.10
3.83
3.82
3.80
3.69
2.48
Triethylene glycol bis(acetophenone)
(CDCl3, 500 MHz)
4.00
4.02
4.07
4.10
4.03
5.99
f1 (ppm)

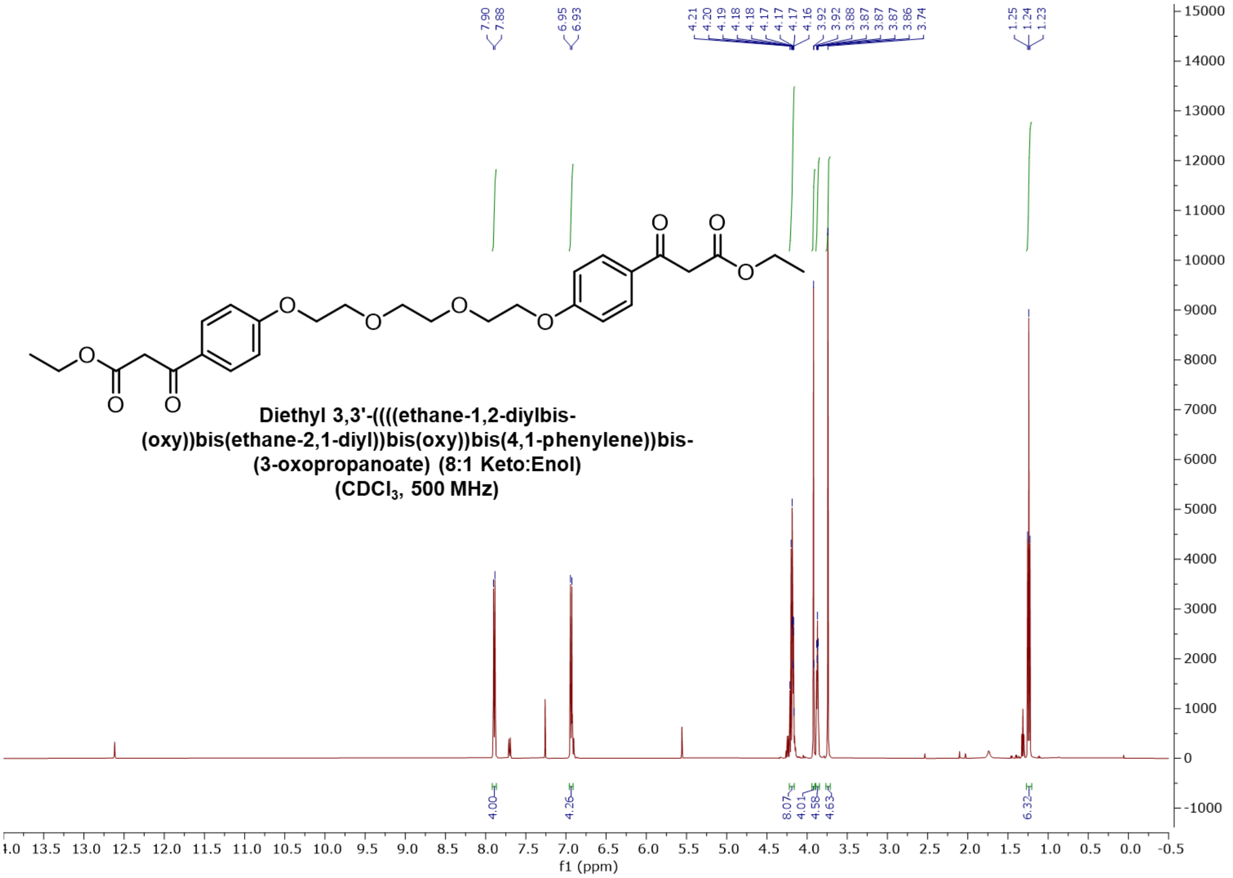
Diethyl 3,3'-((((ethane-1,2-diylbis-
(oxy))bis(ethane-2,1-diyl))bis(oxy))bis(4,1-phenylene))bis-
(3-oxopropanoate) (8:1 Keto:Enol)
($CDCl_3$, 500 MHz)
f1 (ppm)

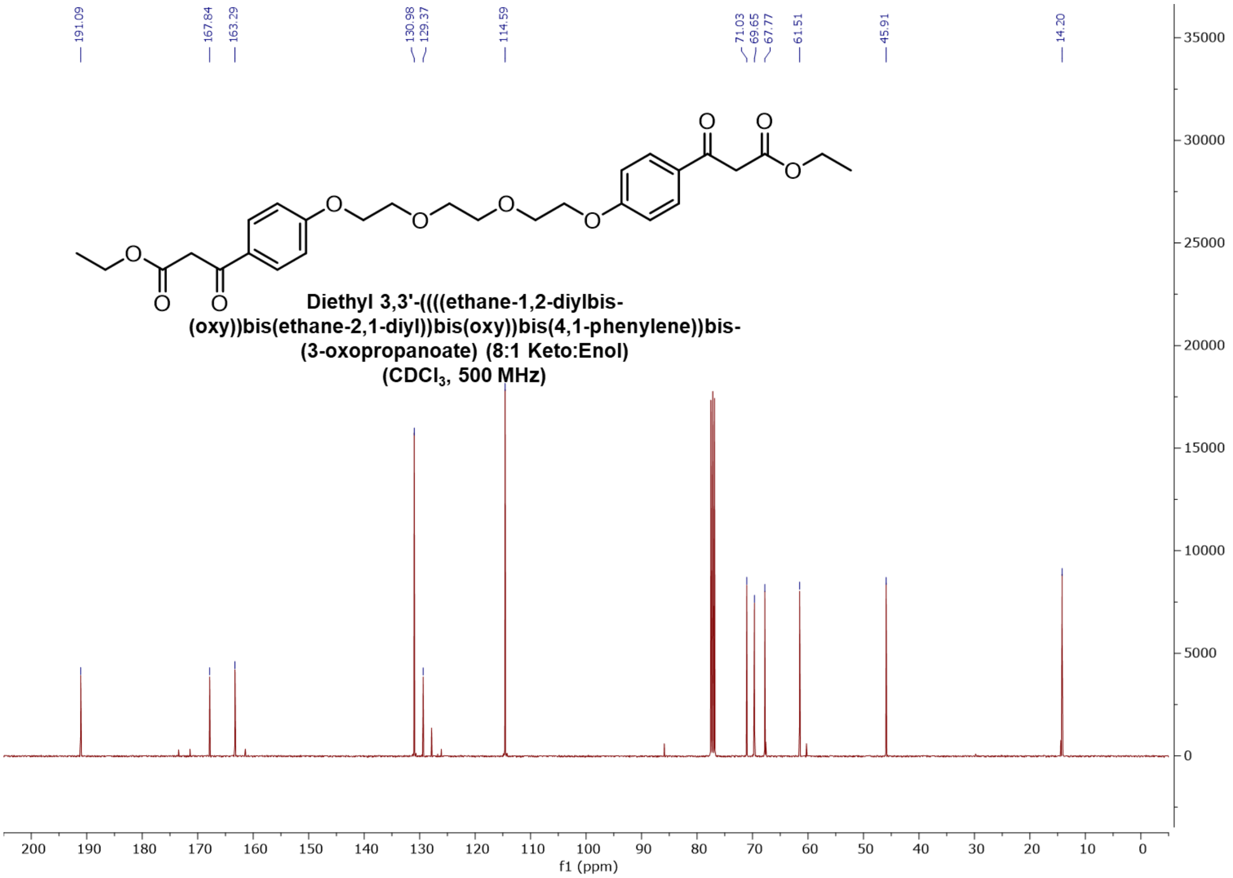

191.09
167.84
163.29
130.98
129.37
114.59
71.03
69.65
67.77
61.51
45.91
14.20
Diethyl 3,3'-((((ethane-1,2-diylbis-
(oxy))bis(ethane-2,1-diyl))bis(oxy))bis(4,1-phenylene))bis-
(3-oxopropanoate) (8:1 Keto:Enol)
(CDCl3, 500 MHz)
f1 (ppm)

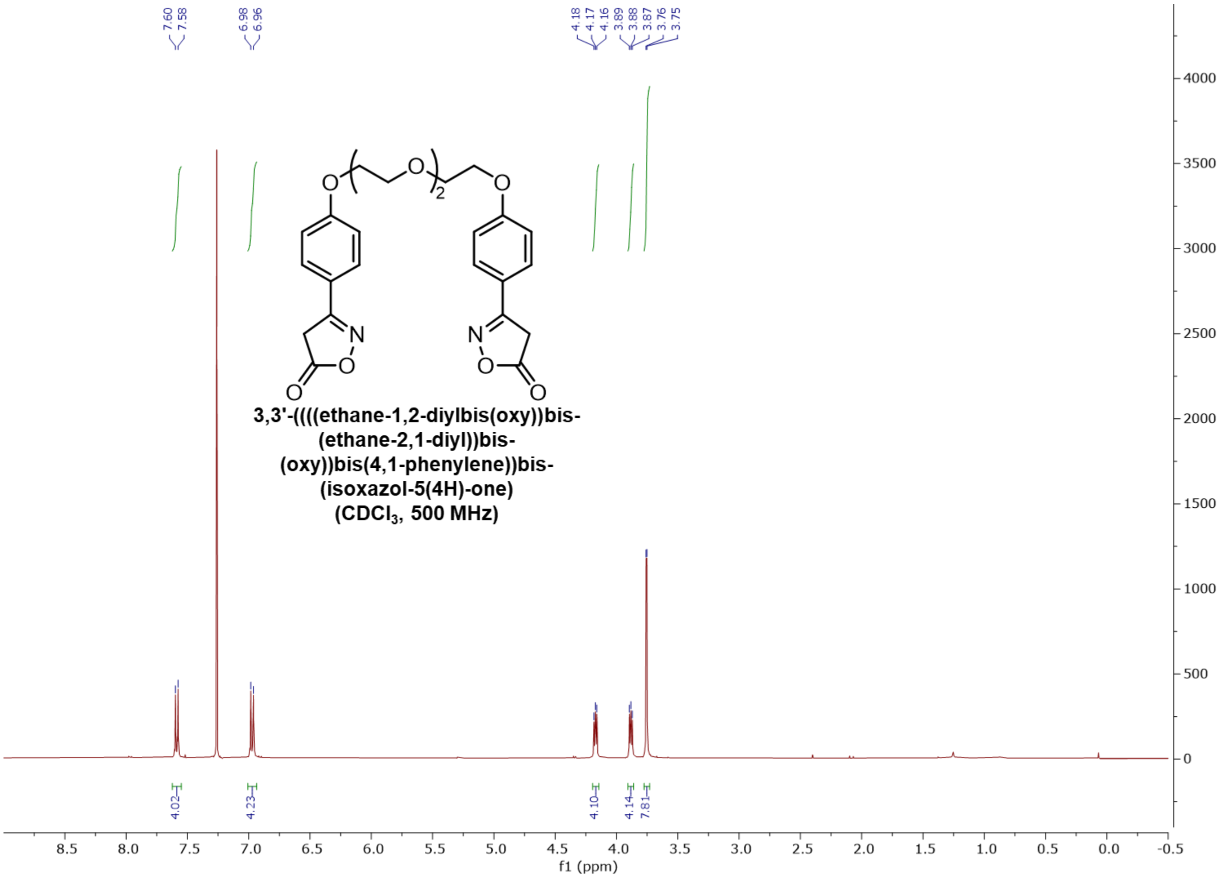

7.60
7.58
6.98
6.96
4.18
4.17
4.16
3.89
3.88
3.87
3.76
3.75
3,3'-((((ethane-1,2-diylbis(oxy))bis-
(ethane-2,1-diyl))bis-
(oxy))bis(4,1-phenylene))bis-
(isoxazol-5(4H)-one)
(CDCl3, 500 MHz)
4.02
4.23
4.10
4.14
7.81
f1 (ppm)

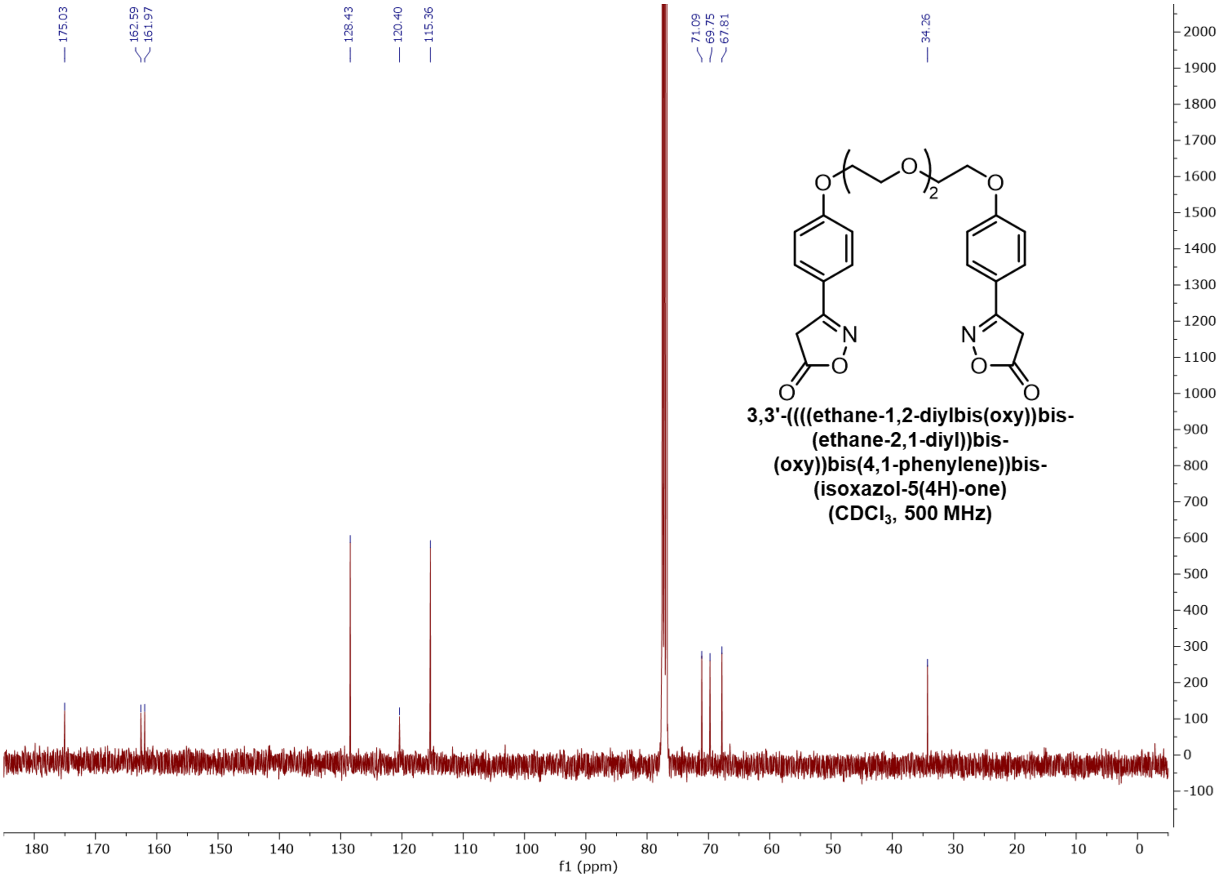
175.03
162.59
161.97
128.43
120.40
115.36
71.09
69.75
67.81
34.26
3,3'-((((ethane-1,2-diylbis(oxy))bis-(ethane-2,1-diyl))bis-(oxy))bis(4,1-phenylene))bis-(isoxazol-5(4H)-one)
(CDCl3, 500 MHz)
f1 (ppm)

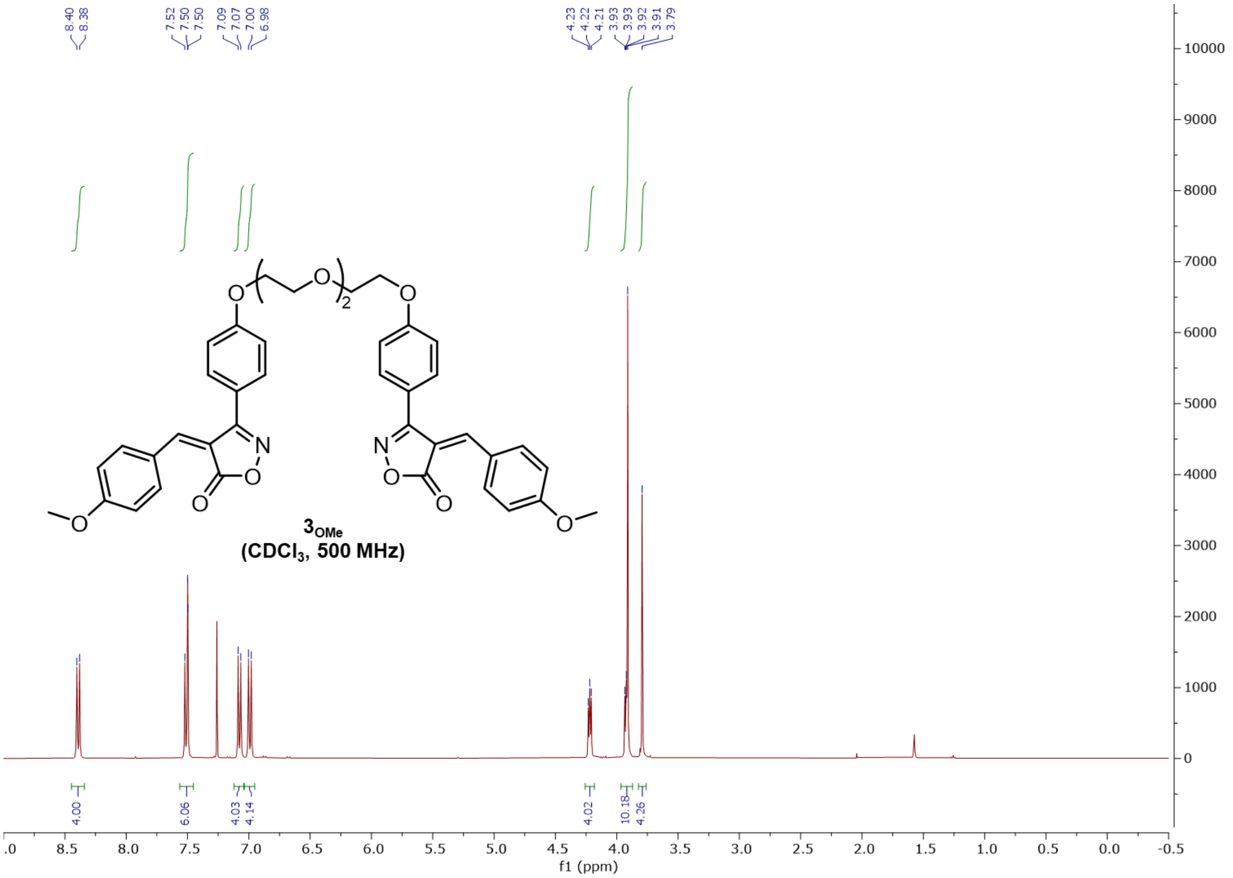
8.40
8.38
7.52
7.50
7.50
7.09
7.07
7.00
6.98
4.23
4.22
4.21
3.93
3.93
3.92
3.91
3.79
O
O
O
O
2
N
O
O
N
O
O
O
O
3OMe
(CDCl3, 500 MHz)
4.00
6.06
4.03
4.14
4.02
10.18
4.26
f1 (ppm)

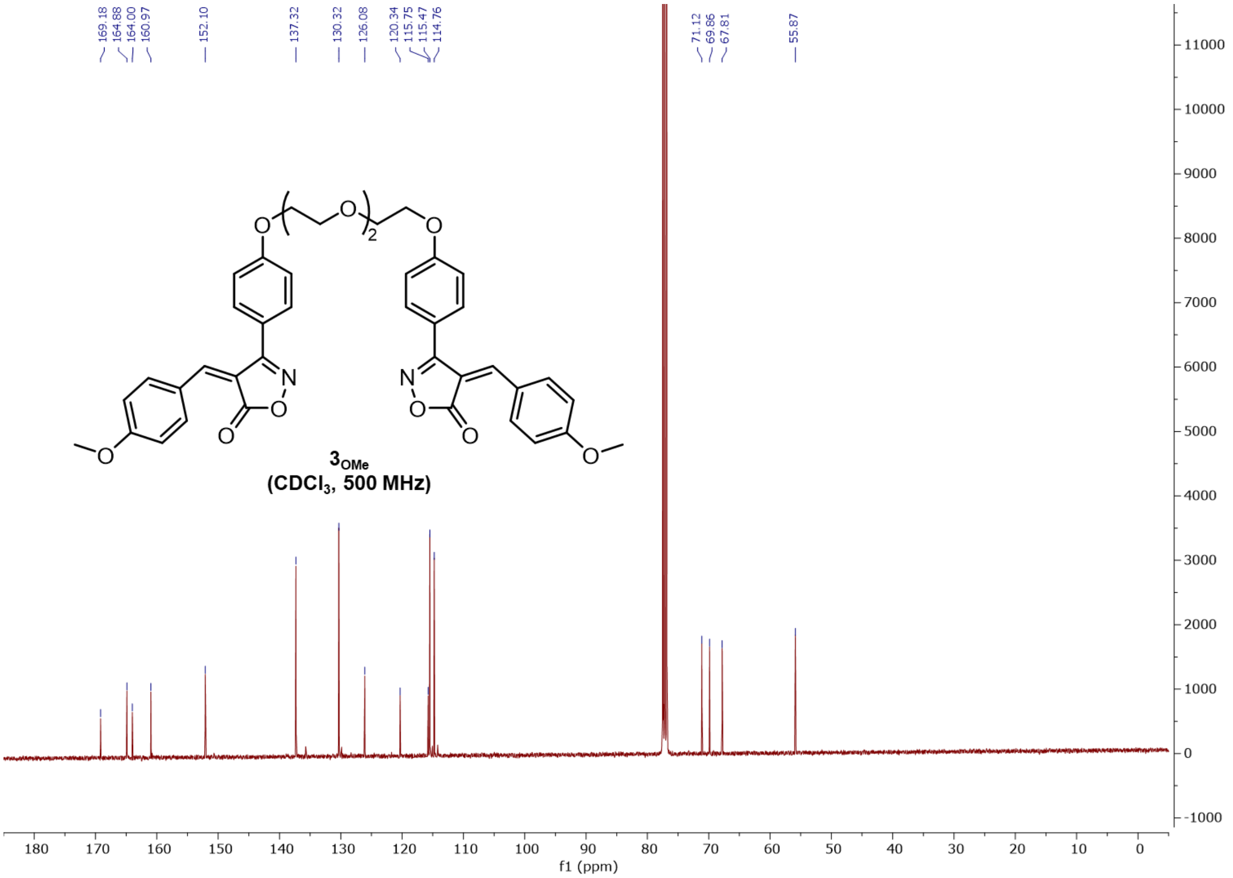
169.18
164.88
164.00
160.97
152.10
137.32
130.32
126.08
120.34
115.75
115.47
114.76
71.12
69.86
67.81
55.87
O
O
2
O
N
O
O
N
O
O
O
O
O
3OMe
(CDCl3, 500 MHz)
11000
10000
9000
8000
7000
6000
5000
4000
3000
2000
1000
0
-1000
180 170 160 150 140 130 120 110 100 90 80 70 60 50 40 30 20 10 0
f1 (ppm)

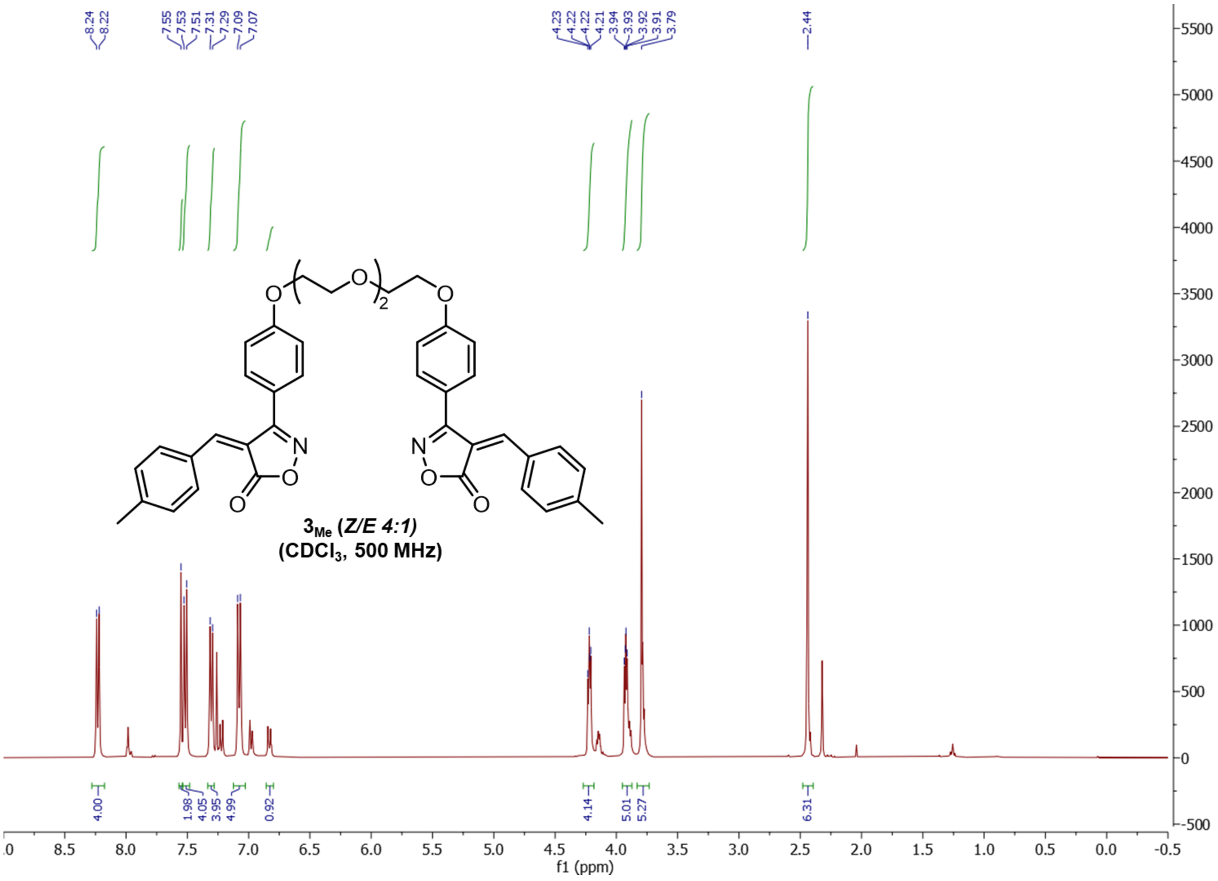
3Me (Z/E 4:1)
(CDCl3, 500 MHz)
f1 (ppm)

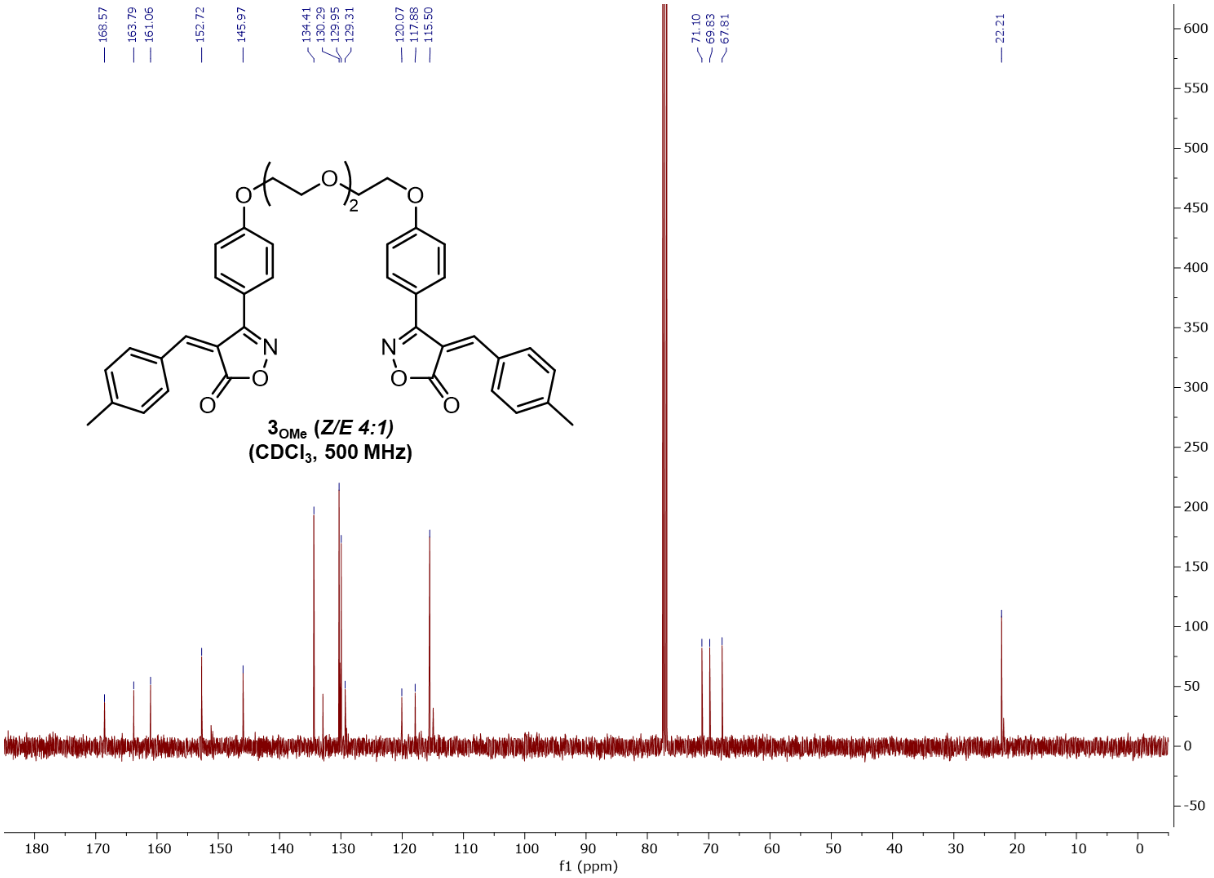
168.57
163.79
161.06
152.72
145.97
134.41
130.29
129.95
129.31
120.07
117.88
115.50
71.10
69.83
67.81
22.21
3OMe (Z/E 4:1)
(CDCl3, 500 MHz)
f1 (ppm)

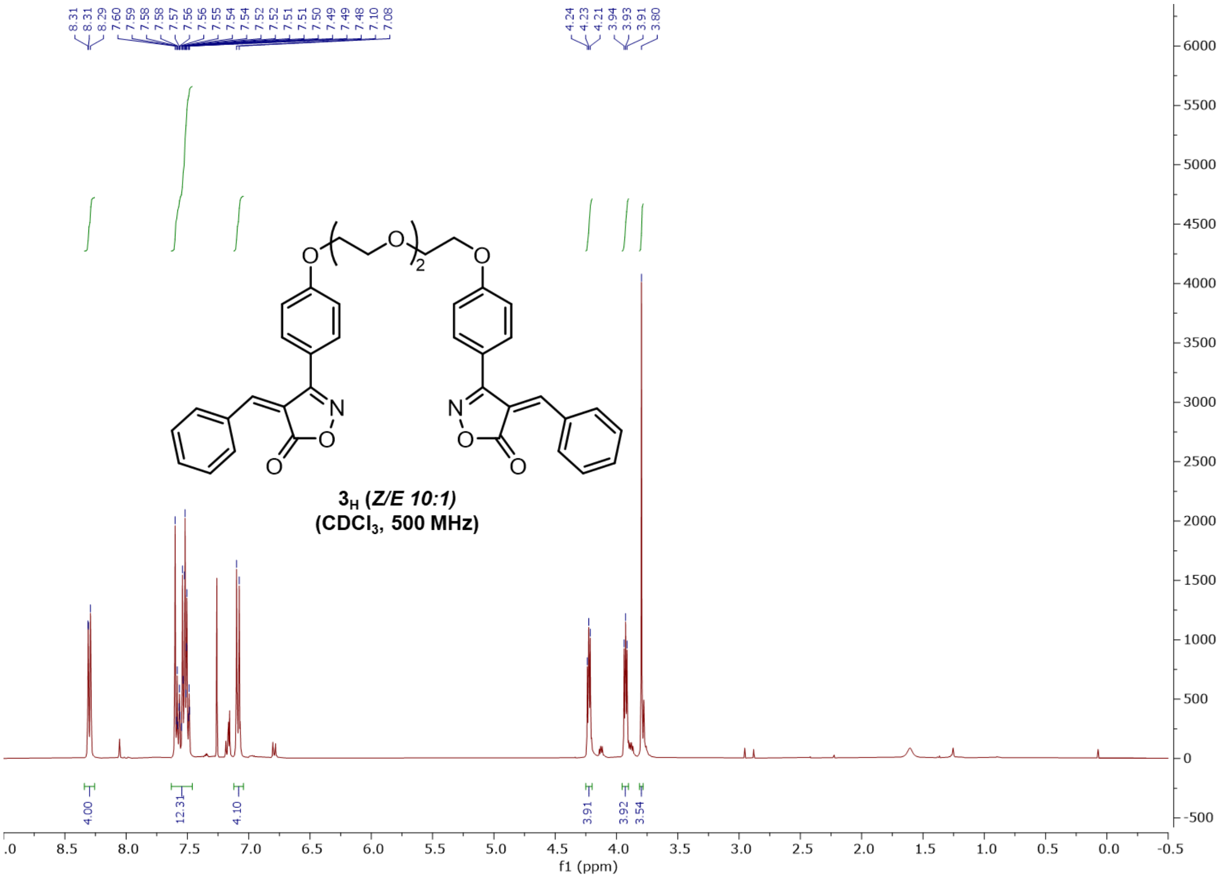

8.31
8.31
8.29
7.60
7.59
7.58
7.58
7.57
7.56
7.56
7.55
7.54
7.54
7.52
7.52
7.51
7.51
7.50
7.49
7.49
7.48
7.10
7.08
4.24
4.23
4.21
3.94
3.93
3.91
3.80
3_H (Z/E 10:1)
($CDCl_3$, 500 MHz)
4.00
12.31
4.10
3.91
3.92
3.54
f1 (ppm)

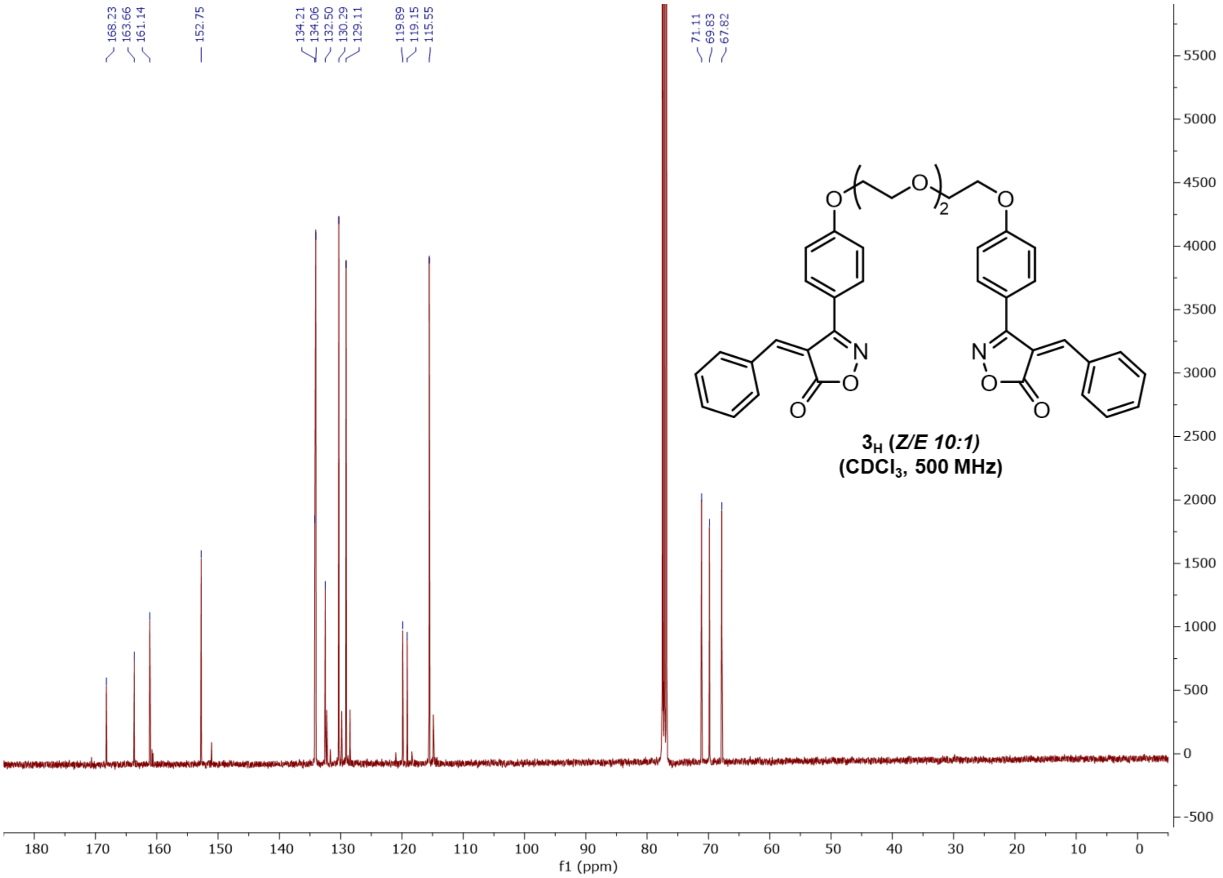
168.23
163.66
161.14
152.75
134.21
134.06
132.50
130.29
129.11
119.89
119.15
115.55
71.11
69.83
67.82
3H (Z/E 10:1)
(CDCl3, 500 MHz)
f1 (ppm)

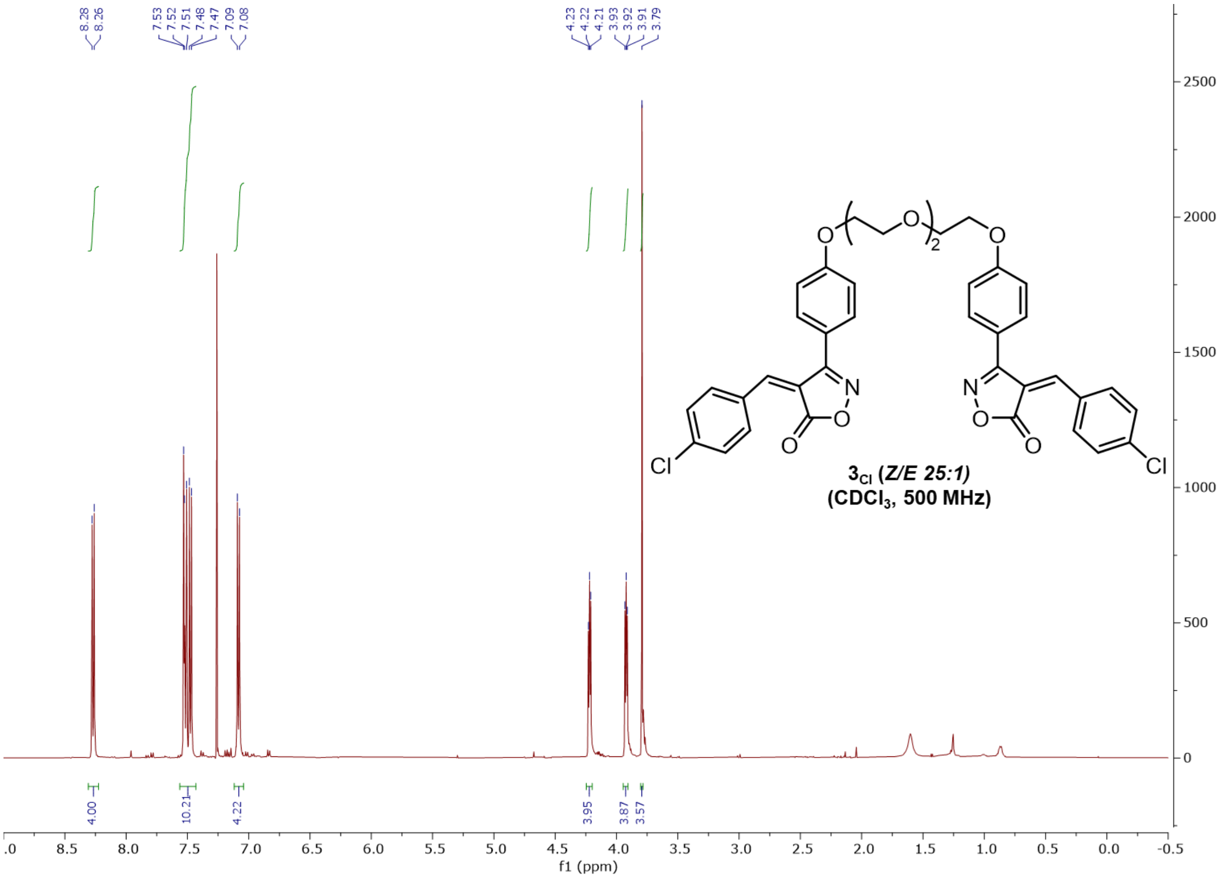
8.28
8.26
7.53
7.52
7.51
7.48
7.47
7.09
7.08
4.23
4.22
4.21
3.93
3.92
3.91
3.79
4.00
10.21
4.22
3.95
3.87
3.57
f1 (ppm)
3Cl (Z/E 25:1)
(CDCl3, 500 MHz)

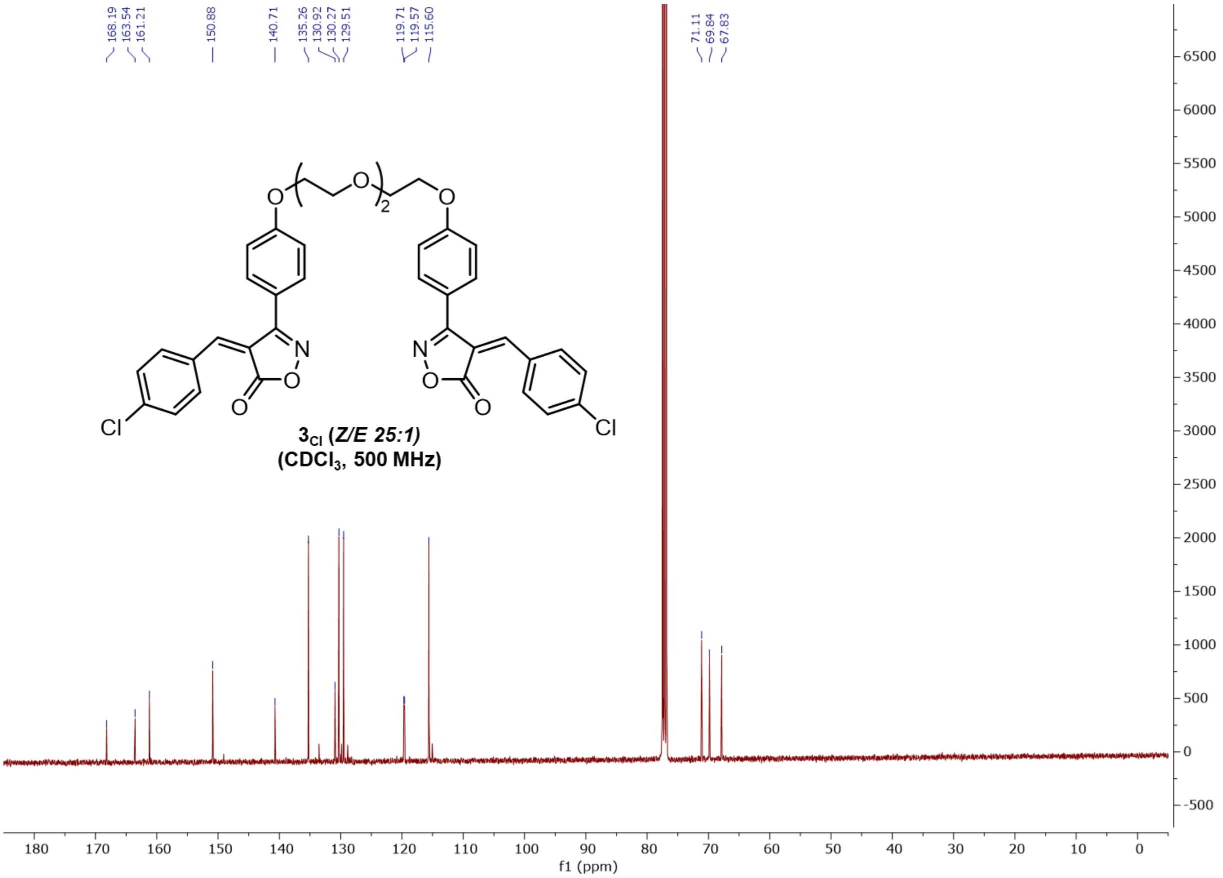

168.19
163.54
161.21
150.88
140.71
135.26
130.92
130.27
129.51
119.71
119.57
115.60
71.11
69.84
67.83
3_{Cl} (*Z/E 25:1*)
($CDCl_3$, 500 MHz)
f1 (ppm)

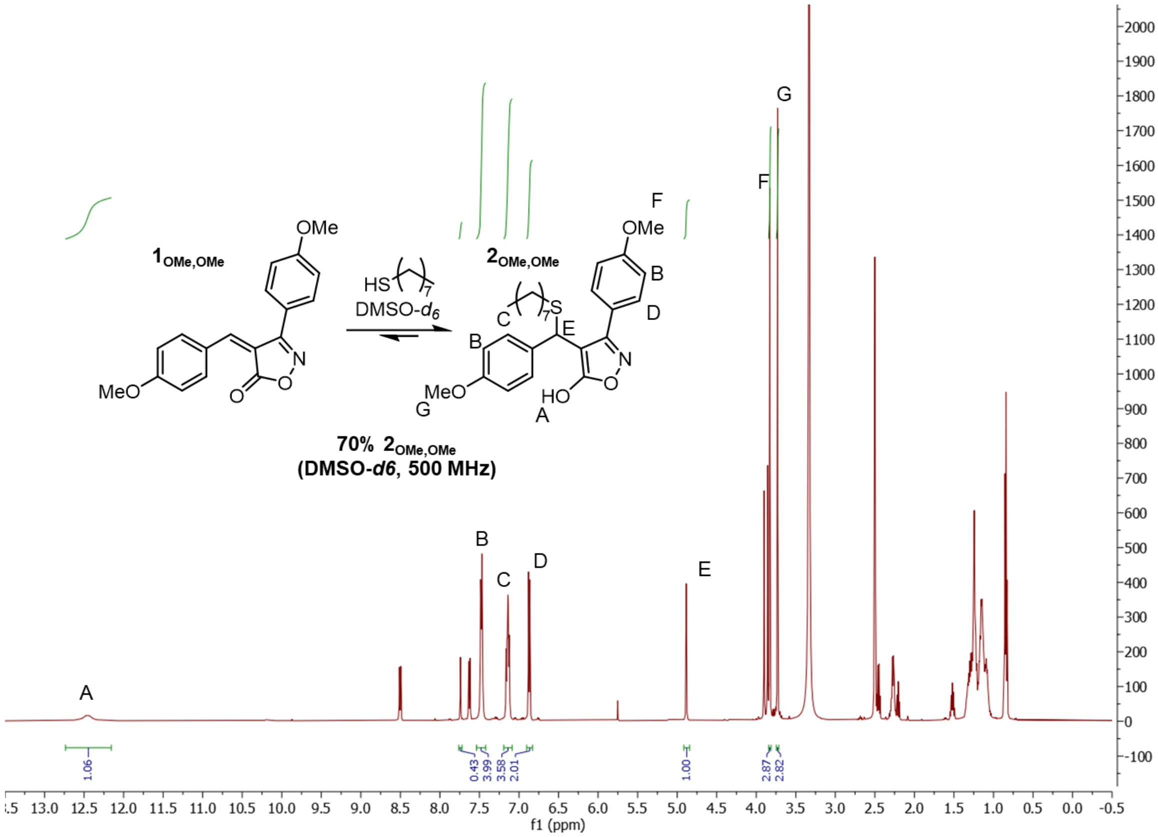
1OMe,OMe
OMe
MeO
HS
DMSO-d6
2OMe,OMe
F
OMe
B
D
C
S
E
B
MeO
G
HO
A
70% 2OMe,OMe
(DMSO-d6, 500 MHz)
A
B
C
D
E
F
G
1.06
0.43
3.99
3.58
2.01
1.00
2.87
2.82
f1 (ppm)

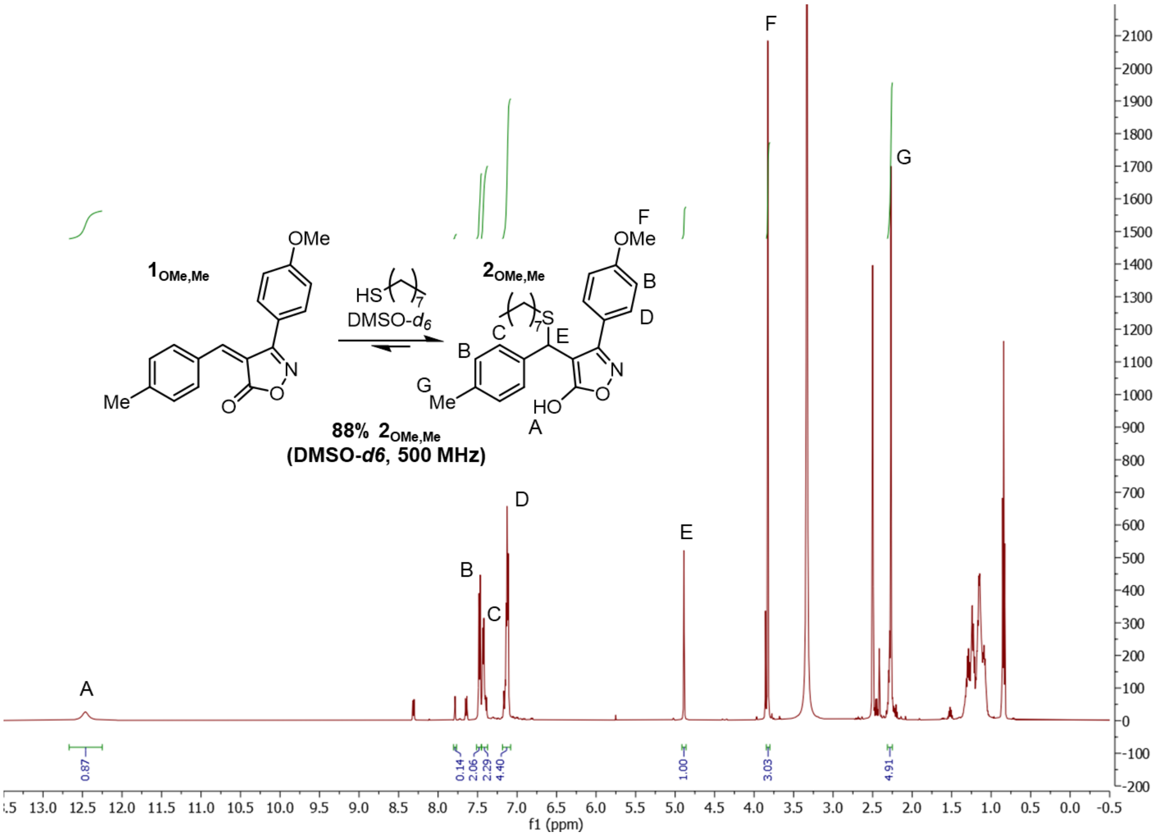

$1_{OMe,Me}$
OMe
Me
O
O
N
HS
7
DMSO-d_6
$2_{OMe,Me}$
F
OMe
B
D
C
S
E
B
G
Me
HO
A
N
O
88% $2_{OMe,Me}$
(DMSO-$d6$, 500 MHz)
A
B
C
D
E
F
G
0.87
0.14
2.06
2.29
4.40
1.00
3.03
4.91
f1 (ppm)

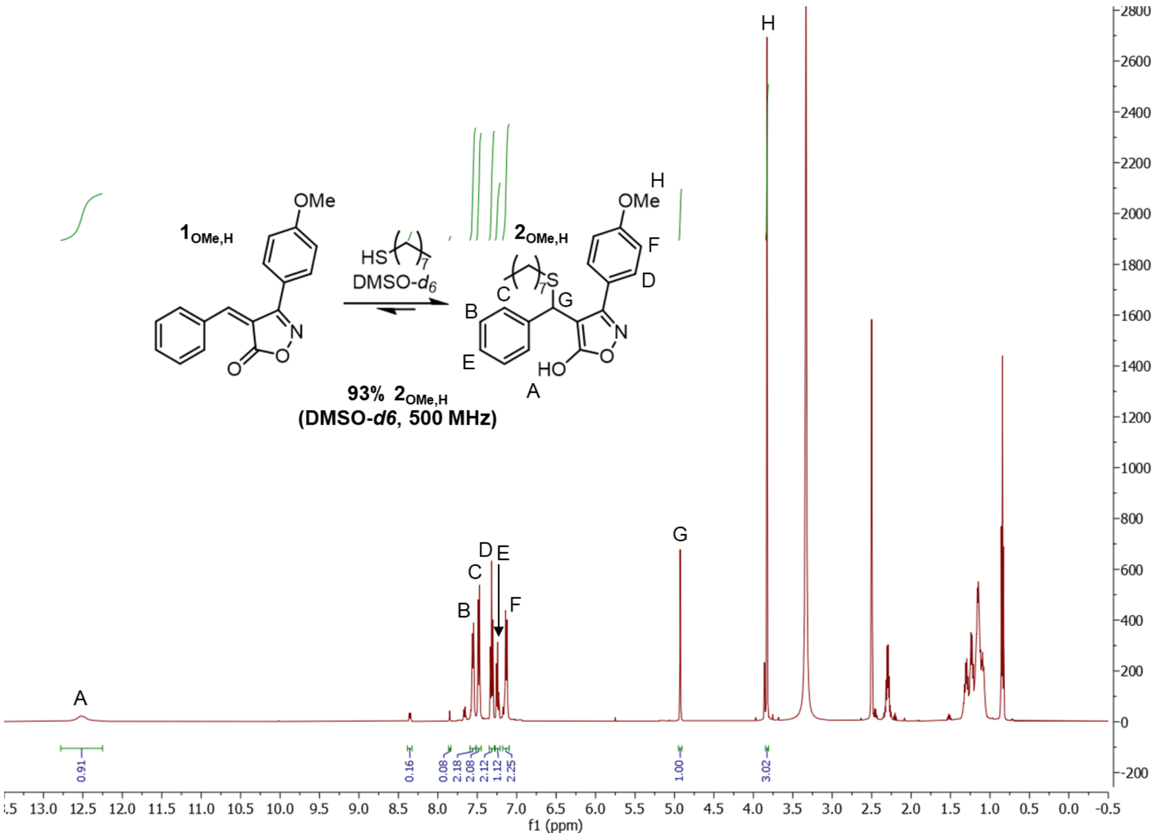

1OMe,H
OMe
HS
7
DMSO-d6
2OMe,H
H
OMe
F
D
C
7
S
G
B
N
O
E
HO
A
93% 2OMe,H
(DMSO-d6, 500 MHz)
H
G
D
E
C
F
B
A
0.91
0.16
0.08
2.18
2.08
2.12
1.12
2.25
1.00
3.02
f1 (ppm)

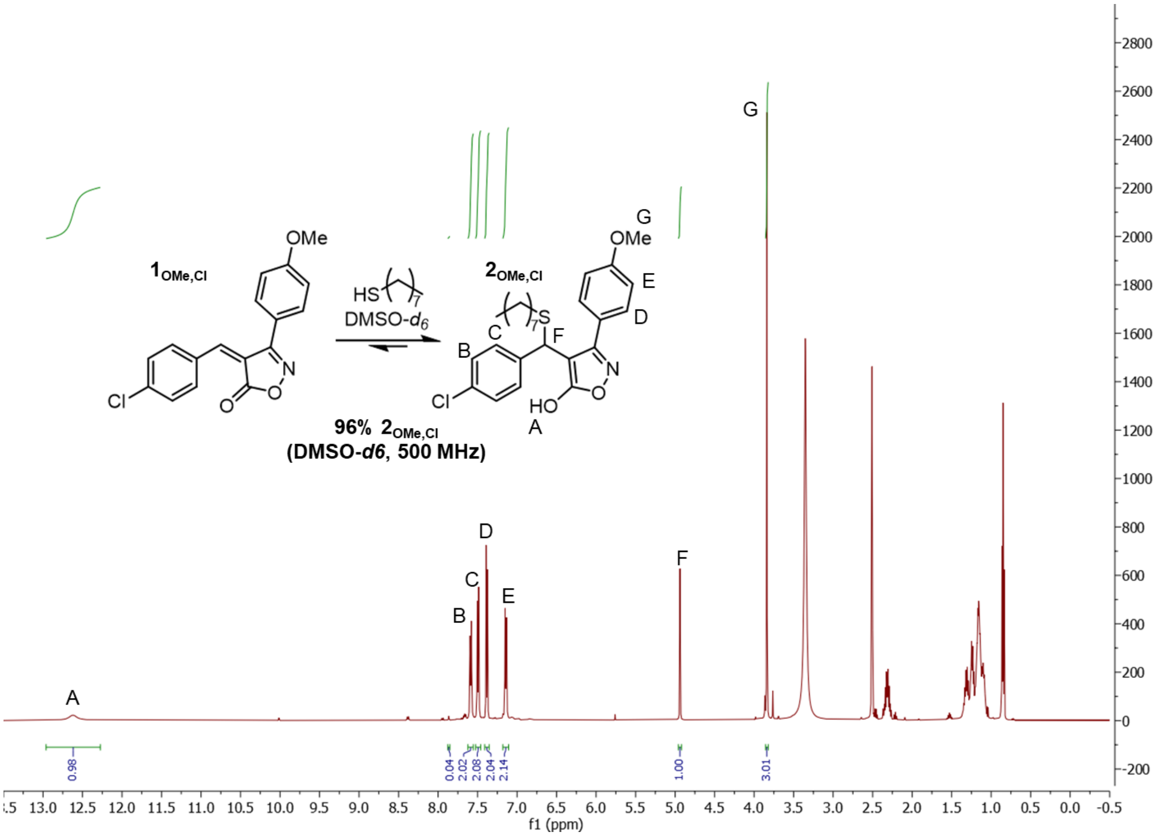

1OMe,Cl
OMe
HS
DMSO-d6
2OMe,Cl
G
OMe
E
D
C
S
F
B
N
Cl
O
HO
A
96% 2OMe,Cl
(DMSO-d6, 500 MHz)
A
B
C
D
E
F
G
0.98
0.04
2.02
2.08
2.04
2.14
1.00
3.01
f1 (ppm)
